# Benzylic C−H acylation by cooperative NHC and photoredox catalysis

**DOI:** 10.1038/s41467-021-22292-z

**Published:** 2021-04-06

**Authors:** Qing-Yuan Meng, Lena Lezius, Armido Studer

**Affiliations:** Organisch-Chemisches Institut, Westfälische Wilhelms-Universität, Münster, Germany

**Keywords:** Organocatalysis, Photocatalysis, Synthetic chemistry methodology

## Abstract

Methods that enable site selective acylation of sp^3^ C-H bonds in complex organic molecules are not well explored, particularly if compared with analogous transformations of aromatic and vinylic sp^2^ C-H bonds. We report herein a direct acylation of benzylic C-H bonds by merging N-heterocyclic carbene (NHC) and photoredox catalysis. The method allows the preparation of a diverse range of benzylic ketones with good functional group tolerance under mild conditions. The reaction can be used to install acyl groups on highly functionalized natural product derived compounds and the C-H functionalization works with excellent site selectivity. The combination of NHC and photoredox catalysis offers options in preparing benzyl aryl ketones.

## Abstract (web summary)

Direct acylation of sp3 C−H bonds in complex organic molecules is not well established compared to that of sp2 C−H bonds. Here, the authors report the combination of N-heterocyclic carbene (NHC) and photoredox catalysis for the direct and site-selective acylation of benzylic C−H bonds.

## Introduction

The Friedel-Crafts acylation (FCA) is a very powerful and established method for the introduction of an acyl group to an electron-rich arene via electrophilic aromatic substitution. It is apparent that the rapid development of transition metal catalyzed arene C–H functionalization has offered strategies to conduct formal Friedel-Crafts acylations. In these modern variants, a stoichiometric amount of a corrosive Lewis acid, generally required in the classical FCA, is not necessary. Moreover, electron-neutral and even electron-poor arenes have become eligible substrates^1^. Although significant progress has been achieved for the acylation of sp^2^ C–H bonds, the analogous transformation on sp^3^ C–H bonds still remains a challenge^2–4^

**Fig. 1:**
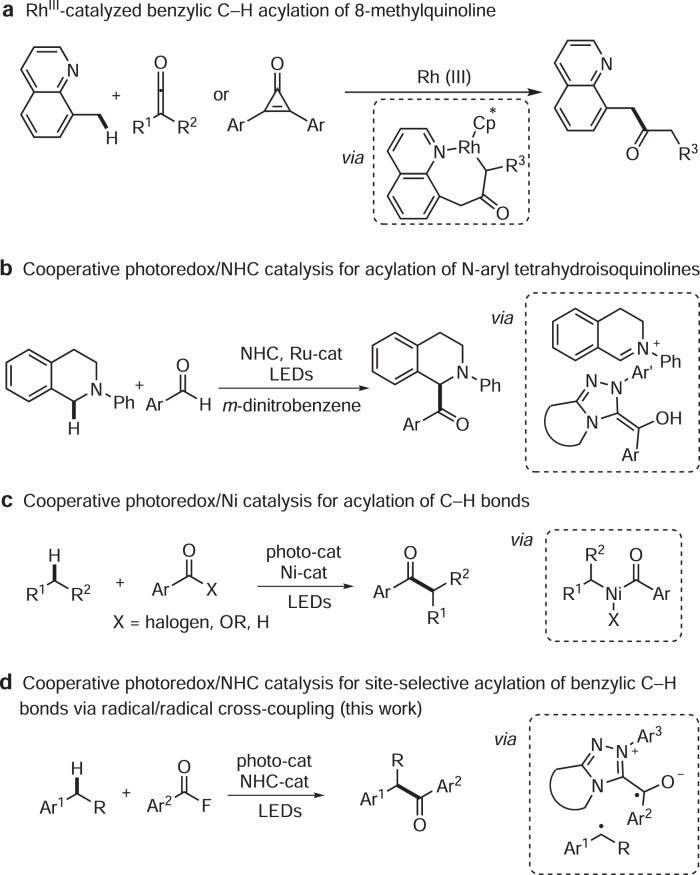
Direct acylation of sp^3^ C-H bonds. **a** Rh^III^-catalyzed benzylic C-H acylation of 8-methylquinoline. **b** Cooperative photoredox/NHC catalysis for acylation of *N*-aryl tetrahydroisoquinolines. **c** Cooperative photoredox/Ni catalysis for acylation of C–H bonds. **d** Cooperative photoredox/NHC catalysis for site-selective acylation of benzylic C–H bonds via radicals cross-coupling. NHC N-heterocyclic carbene, LEDs light-emitting diodes, Rh rhodium, Ru-cat ruthenium catalyst, Ni-cat nickel catalyst.

Benzylic C–H bonds occur in many bioactive compounds and ~25% of the top-selling 200 pharmaceuticals contain this structural motif^5,6^. Great efforts have been devoted to functionalize such C–H bonds and benzylic C–C^7–11^, C–N^12–14^, C–O^15–18^, and C–F bond^19,20^ formation among others^21,22^ have been realized. However, direct benzylic C–H bond acylation is not well explored. A problem is that the targeted aryl ketones can further react in a keto-directed sp^2^ C–H functionalization^23^. Moreover, site-selective acylation is challenging in cases where various benzylic C–H bonds are present. Li and co-workers reported rhodium-catalyzed acylation of 8-methylquinolines with ketenes or cyclopropenones to deliver the corresponding benzylic acylated products^24,25^. Coordinating directing groups were required to control the regioselectivity, thus limiting the applicability of this method (Fig. 1a). The merger of photocatalysis with other catalytic modes has opened avenues for acylation of sp^3^ C–H bonds^26,27^. In 2012, the Rovis group introduced cooperative NHC (N-heterocyclic carbene) and photocatalysis for the asymmetric acylation of activated C–H bonds in *N*-aryl tetrahydroisoquinolines^28^. The key C–C-bond forming step proceeds via an ionic trapping of an oxidatively generated iminium ion with a Breslow-intermediate (Fig. 1b). Doyle, Murakami, Shibasaki, Rueping and Hong employed cooperative nickel and photoredox catalysis to realize C–H acylation of *N*-alkyl-*N*-aryl amines, ethers, unactivated alkanes and benzylic C–H bonds^29–33^. Alkyl acyl Ni^III^ species were proposed as the key intermediates in these transformations and C–H activation was achieved via intermolecular hydrogen atom transfer (HAT; Fig. 1c).

Despite these achievements, the development of other catalytic modes to achieve direct acylation of benzylic C–H bonds with excellent regio- and diastereoselectivity is still highly desirable. Inspired by our previous work on radical acyltrifluoromethylation of alkenes via cooperative photoredox/NHC catalysis^34^ and recent NHC-catalyzed radical reactions^35–45^, we wondered whether ketyl radical cross-coupling with benzylic radicals could be applied for acylation of benzylic C–H bonds. Our strategy is based on the following reactions: single-electron oxidation of an arene will produce a radical cation, which is readily deprotonated to give the corresponding benzylic radical. On the other hand, a persistent ketyl radical can be generated via single-electron-transfer (SET) reduction of an in situ formed acylazolium ion^46^. The SET-oxidation and SET-reduction steps would qualify for an overall redox neutral transformation. The longer-lived ketyl radical and the benzylic radical should then dimerize in a radical/radical cross-coupling to afford after NHC fragmentation the targeted ketone (Fig. 1d).

## Results and discussion

### Reaction conditions development

**Fig. 2:**
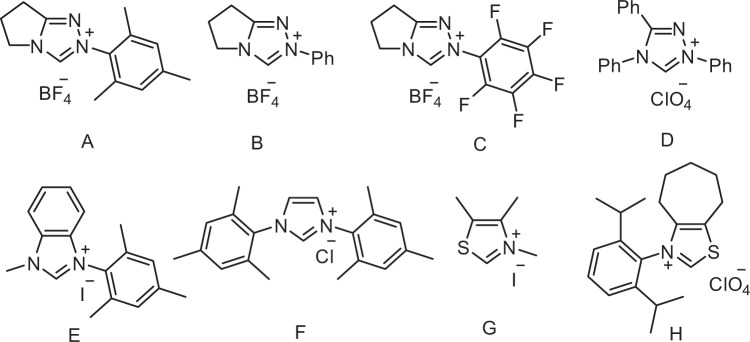
Different NHC catalysts tested. A–D are precursors leading to triazol-5-ylidene-type carbenes, E and F provide imidazole-2-ylidines, and G and H deliver thiazol-2-ylidenes. NHC N-heterocyclic carbene.

To test our hypothesis, we initiated the study by examining the cross-coupling between benzoyl fluoride (**1a**) and 4-ethyl anisole (**2a**) with the triazolium salt A as the catalyst, which has been shown to be efficient in multi component couplings^34^. [Ir(dF(CF_3_)ppy)_2_(dtbbpy)]PF_6_ was selected as a photocatalyst because its excited state is a strong oxidant (*E*_1/2_[Ir^*III/II^] = +1.21 V)^47^. Although 4-ethyl anisole shows a higher oxidation potential (*E*_1/2_ = +1.52 V)^48^, we speculated that the endothermic SET can be productive if the subsequent deprotonation is fast^6^. Indeed, after irradiation for 24 h in dichloromethane, a 15% yield of the targeted **3a** was obtained. However, conversion was low (Table 1, entry 1). Solvent optimization revealed that improved yields can be achieved in dimethylformamide and acetonitrile with the latter showing the best result (entries 2–8). The NHC-screening identified the triazolium salt A as the ideal precatalyst, and all other triazoliums, imidazoliums, and thiazolium salts B-H tested, provided lower yields. In two cases (E and G), no conversion was noted (entries 9–15, Fig. 2). Yield and conversion could be further improved upon running the cascade at higher concentration (entries 16–18). It is notable that the donor–acceptor dye 2,4,5,6-tetra(carbazol-9-yl)isophthalonitrile (4CzIPN)^49^ showed the same catalytic efficiency as the Ir-based photocatalyst (entry 19). Decreasing the amount of base, NHC precatalyst, or photocatalyst led to lower yields (SI, Supplementary Table 1). Finally, control experiments validated the necessity of carbene and the photocatalyst for successful C–H benzoylation of **2a**. No product was observed in the absence of light, photocatalyst, or carbene (entries 20–22). Importantly, the same two substrates that qualify for classical Friedel-Crafts acylation using a Lewis acid as an activator will provide a different product upon switching to the NHC/photocatalyst activation mode in a chemodivergent manner. For example, the reaction of benzoyl fluoride (**1a**) with 4-ethyl anisole (**2a**) in the presence of a stoichiometric amount of AlCl_3_ afforded the Friedel-Crafts product in 72% yield. Acylation occurred with excellent regioselectivity at the *ortho*-position of the activating methoxy group (see SI for details).

**Table 1:** Optimization of the reaction conditions^a^.


Entry	Solvent	NHC precatalyst	2a, (M)	Conversion (%)^b^	Yield (%)^c^
1	CH_2_Cl_2_	A	0.05	17	15
2	CHCl_3_	A	0.05	24	22
3	DMSO	A	0.05	20	20
4	DMF	A	0.05	43	42
5	1,4-Dioxane	A	0.05	5	5
6	Toluene	A	0.05	5	4
7	CH_3_CN	A	0.05	60	59
8	Ethyl acetate	A	0.05	4	4
9	CH_3_CN	B	0.05	43	42
10	CH_3_CN	C	0.05	3	3
11	CH_3_CN	D	0.05	7	6
12	CH_3_CN	E	0.05	0	0
13	CH_3_CN	F	0.05	3	3
14	CH_3_CN	G	0.05	0	0
15	CH_3_CN	H	0.05	2	0
16	CH_3_CN	A	0.07	77	77
17	CH_3_CN	A	0.10	86	85
18	CH_3_CN	A	0.17	92	88 (83)
19^d^	CH_3_CN	A	0.17	–	83
20^e^	CH_3_CN	A	0.17	0	0
21^f^	CH_3_CN	A	0.17	0	0
22^g^	CH_3_CN		0.17	0	0

^a^Reaction conditions: unless otherwise noted, all the reactions were carried out with benzoyl fluoride (0.4 mmol), 4-ethyl anisole (0.1 mmol), NHC catalyst (0.02 mmol), Cs_2_CO_3_ (0.2 mmol), and [Ir(dF(CF_3_)ppy)_2_(dtbbpy)]PF_6_ (0.002 mmol) in anhydrous CH_3_CN (2 mL), irradiation with blue LEDs at room temperature for 24 h.

^b^GC-FID conversion using 1,3,5-trimethoxybenzene as an internal standard.

^c1^H NMR yield using 1,3,5-trimethoxybenzene as an internal standard and yield of isolated product is given in parentheses.

^d^4CzIPN (0.002 mmol) was used instead of [Ir(dF(CF_3_)ppy)_2_(dtbbpy)]PF_6_ as the photocatalyst.

^e^The reaction was carried out in the dark.

^f^No photocatalyst was added.

^g^No NHC catalyst was added. 4CzIPN, 2,4,5,6-tetra(carbazol-9-yl)isophthalonitrile. rt, room temperature. NHC, *N*-heterocyclic carbene. LEDs, light-emitting diodes.

### Substrate scope

**Fig. 3:**
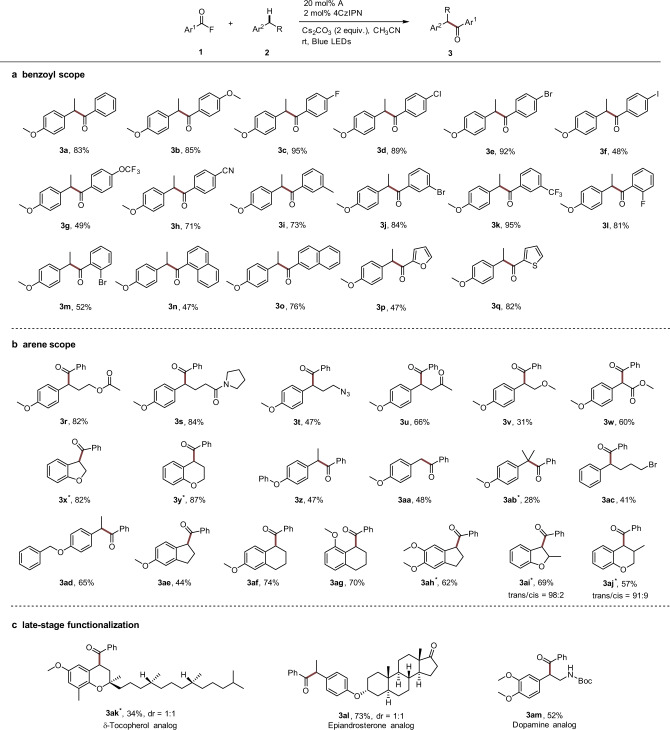
The reaction scope. **a** Benzoyl scope. **b** Arene scope. **c** Late-stage functionalization. Reaction conditions: all the reactions were carried out with benzoyl fluoride (0.4 mmol), arene (0.1 mmol), NHC precatalyst A (0.02 mmol), Cs_2_CO_3_ (0.2 mmol), and 4CzIPN (0.002 mmol) in anhydrous CH_3_CN (0.6 mL), irradiation with blue LEDs at room temperature for 24 h. Isolated yields were given. ^*^[Ir(dF(CF_3_)ppy)_2_(dtbbpy)]PF_6_ (0.002 mmol) was used instead of 4CzIPN. See SI for more details. 4CzIPN, 2,4,5,6-tetra(carbazol-9-yl)isophthalonitrile. LEDs light-emitting diodes.

With the optimized conditions established, we examined the reaction scope with respect to the acyl fluoride first (Fig. 3a). A wide range of benzoyl fluorides bearing electron-donating or electron-withdrawing substituents could be used for the C–H aroylation of 4-ethyl anisole, affording the desired products in moderate to excellent yields (**3b**–**3m**, 48–95%). As is evident from these results, aryl halides, most of which would be incompatible with Ni catalysis^29–33^, engage in this cascade, albeit a moderate yield was obtained for the iodo-congener (**3f**, 48%). α-Naphthoyl and β-Naphthoyl fluoride were successfully used in the direct aroylation of **2a** (**3n**, 47%; **3o**, 76%). The latter was more reactive due to lower steric hindrance during formation of the β-naphthoyl azolium intermediate. Furthermore, heteroaroyl fluorides containing the furan and thiophene moieties were also amenable to the coupling with **2a** to afford **3p** and **3q** in 47% and 82% yields, respectively.

The scope of the reaction with respect to the benzylic component was explored next using benzoyl fluoride **1a** as the acyl donor (Fig. 3b). A variety of functional groups are tolerated, giving rise to products bearing ester (**3r**, 82%; **3w**, 60%), amide (**3s**, 84%), azide (**3t**, 47%), ketone (**3u**, 66%), and ether (**3v**, 31%) functionalities. It is important to note that the 1,2-dihydrobenzfuran and chroman substructures can be found in pharmaceutical drugs such as Darifenacin and Nibivolol^50,51^. With this in mind, we tested them as radical coupling partners and both systems worked well to afford the ketones **3x** and **3y** in 82% and 87% yields, respectively. Benzoylation in α-position to the O-atom was not observed, clearly showing that C-radical formation occurs with complete regioselectivity at the benzylic position. Compared with 4-ethyl anisole, 1-ethyl-4-phenoxybenzene showed a lower efficiency (**3z**, 47%). Primary benzylic C–H bonds could also be acylated to give the desired products (**3aa**, 48%). However, reaction was sluggish and 48% of starting material was recovered. Owing to the steric hindrance of tertiary C–H bonds, lower yields were obtained for **3ab** (28%). Importantly, an activating O-substituent decreasing the arene oxidation potential is not required and (4-bromobutyl)benzene could be benzoylated with **1a**, albeit with moderate yield (**3ac**, 41%).

We next turned our attention to examine the site selectivity of the direct C-H benzoylation. Even in the presence of weaker benzylic C–H bonds, exclusive functionalization of the ethyl moiety in *para*-position to the alkoxy group was noted (**3ad**, 65% yield). Along these lines, methoxy-substituted dihydroindene and tetrahydronaphthalenes reacted with excellent site selectivity at the benzylic position that is located *ortho* or *para* to the activating methoxy group (**3ae**, 44%; **3af**, 74%; **3ag**, 70%). Likely, intermolecular C–H abstraction with an electrophilic radical would be not regioselective in these cases, showing that the deprotonation of an arene radical cation under our conditions is a highly regioselective process. It is noteworthy that monobenzoylation of 5,6-dimethoxy-2,3-dihydro-1H-indene was achieved (**3ah**, 62% yield). 1,2-Dihydrobenzfuran and chroman bearing a methyl substituent adjacent to the reactive benzylic site delivered the targeted ketones with high diastereoselectivities in good yields (**3ai**, 69%, trans/cis = 98:2; **3aj**, 57%, trans/cis = 91:9). Finally, to further demonstrate the potential of our method, we applied the radical/radical cross-coupling reaction to the late-stage benzylic benzoylation of biologically interesting compounds (Fig. 3c). Highly regioselective benzoylation of the methylene moiety of a δ-tocopherol analog was achieved and benzoylation of the methyl group did not occur (**3ak**, 34%, dr = 1:1). Additionally, both epiandrosterone and dopamine analogs could be regioselectively functionalized at the benzylic positions, with no byproduct observed derived from reactions adjacent to the ketone or amide moieties (**3al**, 73%; **3am**, 52%).

### Control experiments

**Fig. 4:**
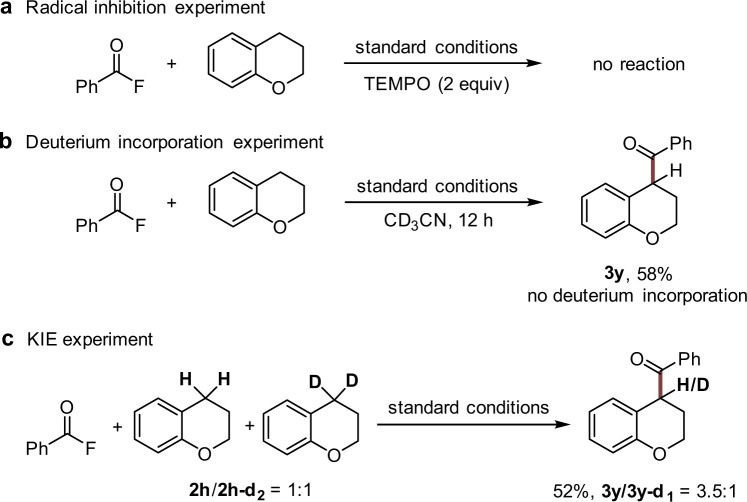
Control experiments. **a** Radical inhibition experiment. **b** Deuterium incorporation experiment. **c** KIE experiment. TEMPO 2,2,6,6-tetramethyl-piperidin-1-oxyl, KIE kinetic isotope effect.

To gain insights into the reaction mechanism, control experiments were performed. C-H-benzoylation of chroman with **1a** was fully suppressed in the presence of 2,2,6,6-tetramethyl-piperidin-1-oxyl (TEMPO) (Fig. 4a). When CD_3_CN was used in place of CH_3_CN, there was no deuterium incorporation into the product, as well as in the recycled starting chroman, which indicates that H-atoms or protons of the solvent do not participate in the cascade (Fig. 4b). Moreover, a kinetic isotope effect (KIE) was observed in the intermolecular competition experiment, demonstrating that the deprotonation might be involved in the rate-determining step (Fig. 4c) ^52^.

### Reaction mechanism

**Fig. 5:**
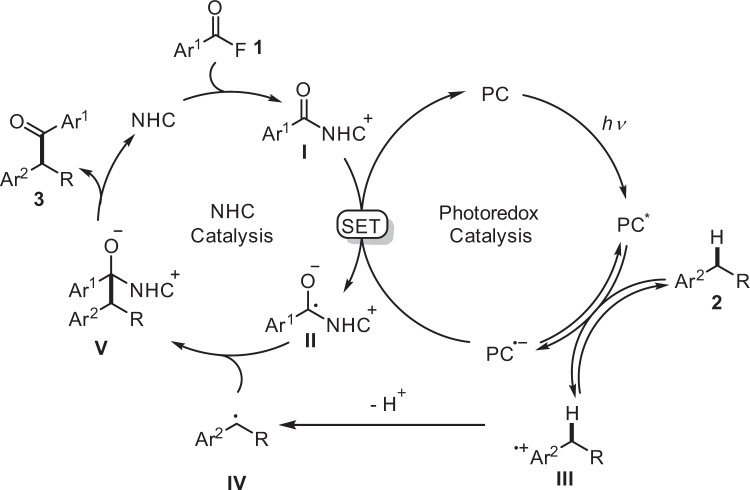
Plausible reaction mechanism. Cooperative NHC/photoredox catalysis where the acylazolium ion **I** is involved in both catalytic cycles. PC photocatalyst, NHC N-heterocyclic carbene, SET single-electron-transfer.

According to the above results and previous reports, a possible mechanism is suggested in Fig. 5. Under blue LEDs irradiation, [Ir(dF(CF_3_)ppy)_2_(dtbbpy)]PF_6_ will be excited and the excited state will be reductively quenched by the electron-rich arene **2**, leading to an arene radical cation **III** with concomitant formation of a radical anion of [Ir(dF(CF_3_)ppy)_2_(dtbbpy)]PF_6_ (*E*_1/2_(P/P^•-^) = − 1.37 V vs SCE) or 4CzIPN (*E*_1/2_(P/P^•-^) = − 1.21 V vs SCE)^47,49^. The radical cation **III** will be deprotonated (Cs_2_CO_3_) at the benzylic position to generate a transient benzylic radical **IV**. Based on the KIE studies, the initial electron transfer from **2** to PC* is reversible. The reduced photocatalyst will then transfer an electron to the acyl azolium salt **I** (*E*_1/2_ = −1.29 V *vs* SCE)^40^ itself formed in situ from the acyl fluoride **1** and the NHC catalyst to generate a persistent ketyl type radical **II**, closing the photoredox catalysis cycle. Radical/radical cross coupling of the transient radical **IV** with the persistent ketyl radical **II** steered by the persistent radical effect^53^ will lead to intermediate **V**. Fragmentation of the NHC will eventually provide the product ketone **3** thereby liberating the NHC catalyst.

In this work, we have developed a strategy to accomplish acylation of benzylic C–H bonds via cooperative NHC and photocatalysis. The key step of the cascade is a radical/radical cross-coupling. The acylation occurs with excellent site selectivity and broad functional group compatibility. The protocol is amenable to functionalize important structural motifs with good to excellent diastereoselectivities, as well as to the late-stage benzoylation of more complex natural product derived compounds. The method will open avenues in the area of direct C–H bond acylation and also complements existing transition metal catalyzed C–H bond functionalization methodologies.

## Methods

### General procedure for the cross-coupling between an acyl fluoride and a benzylic component

To a Schlenk tube the carbene precatalyst A (6.3 mg, 0.02 mmol), 4CzIPN (1.6 mg, 0.002 mmol) or [Ir(dF(CF_3_)ppy)_2_(dtbbpy)]PF_6_ (2.2 mg, 0.002), and Cs_2_CO_3_ (65.2 mg, 0.2 mmol) were added. Then the reaction tube was evacuated and backfilled with argon two times. Subsequently, a benzylic component (0.10 mmol) and an acyl fluoride (0.40 mmol) (if solid, they should be added at the beginning) and CH_3_CN (0.6 mL) were added. The resulting mixture was degassed under vacuum two times and then the mixture was irradiated with blue LEDs at room temperature for 24 h. After that, the residue was purified by silica gel chromatography using a mixture of n-pentane and ethyl acetate or pentane and diethylether as an eluent to get the desired product. Each reaction was carried out twice and the average value was used as the final yield.

## Supplementary information

Supplementary Information

## Data Availability

Supplementary information is available in the online version of the paper. Data supporting the findings of this work are available within this paper or its Supplementary Information and also from the corresponding author upon reasonable request.
