# Supplementary material for: Benzylic C−H acylation by cooperative NHC and photoredox catalysis
Source: Nat Commun. 2021 Apr 6;12:2068. doi: 10.1038/s41467-021-22292-z (PMC8024347; doi:10.1038/s41467-021-22292-z)
Supplement: Supplementary file 1 — Supplementary Information [file 41467_2021_22292_MOESM1_ESM.pdf]

Supplementary Information for

Benzylic C-H Acylation by Cooperative NHC and Photoredox Catalysis

Studer *et al.*

Correspondence to: studer@uni-muenster.de

This PDF file includes:

Supplementary Methods

Supplementary Table 1

Synthesis of substrates and characterization data

Characterization data for products

^{1}H, ^{13}C and ^{19}F NMR spectra

Supplementary references

Supplementary Methods

General information: All reactions involving air- or moisture-sensitive reagents or intermediates were carried out in pre-heated glassware under an argon atmosphere using standard *Schlenk* techniques. All styrene derivatives were distilled prior to use, other commercially available reagents were purchased from TCI, Sigma-Aldrich, Alfa Aesar, Acros, ABCR or BLD pharm in the highest purity grade and used directly without further purification. Thin layer chromatography (TLC) was performed on Merck silica gel 60 F-254 plates and visualized by fluorescence quenching under UV light or staining with the standard solution of $KMnO_4$. Column chromatography was performed on Merck or Fluka silica gel 60 (40-63 μm) using a forced flow of 0.5 bar. ^{1}H NMR, ^{13}C NMR and ^{19}F NMR spectra were recorded on *DPX* 300, *AV 400 or 600* at 300 K. Chemical shifts were expressed in parts per million (ppm) with respect to the residual solvent peak. Coupling constants were reported as Hertz (Hz), signal shapes and splitting patterns were indicated as follows: s, singlet; brs, broad singlet; d, doublet; t, triplet; q, quartet; m, multiplet. Mass spectra were recorded on a *Finnigan MAT 4200S*, a *Bruker Daltonics Micro Tof,* a *Waters-Micromass Quatro LCZ* (ESI); peaks are given in *m/z* (% of basis peak). Reactions were performed with Blue LEDs (3 W). IR spectra were recorded on a Digilab 3100 FT-IR Excalibur Series spectrometer, signal intensities are categorized in strong (s), middle (m) and weak (w). Melting points were measured on a Stuart SMP10 and are uncorrected. Substrates that are not commercially available were synthesized according to the reported literatures, shown below. The triazolium salt A,[1,2] 2,4,5,6-tetra(carbazol-9-yl)isophthalonitrile (4CzIPN)[3] and $[Ir(dF(CF_3)ppy)_2(dtbbpy)]PF_6$[4] were synthesized according to the reported procedures.

General procedure for the benzylic C-H acylation

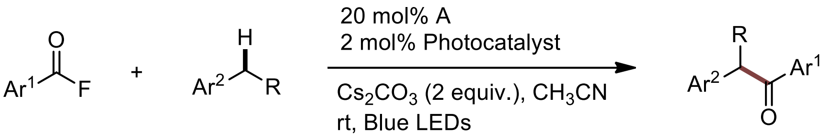

1 2 3

To a Schlenk tube were added carbene catalyst A (6.3 mg, 0.02 mmol), 4CzIPN (1.6 mg, 0.002 mmol) or $[Ir(dF(CF_3)ppy)_2(dtbbpy)]PF_6$ (2.2 mg, 0.002) and Cs_2CO_3 (65.2 mg, 0.2 mmol). Then the reaction tube was evacuated and backfilled with argon two times. Subsequently, a benzylic component (0.10 mmol) and an acyl fluoride (0.40 mmol) (if solid, they should be added at the beginning) and CH_3CN (0.6 mL) were added. The resulting mixture was degassed under vacuum two times and then the mixture was irradiated with blue LEDs at room temperature for 24 h. After that, the residue was purified by silica gel chromatography using a mixture of n-pentane and ethyl acetate or pentane and diethylether as an eluent to get the desired product. Each reaction was carried out twice and the average value was used as the final yield.

Supplementary Table 1 Changing of the amount of reactants[a]

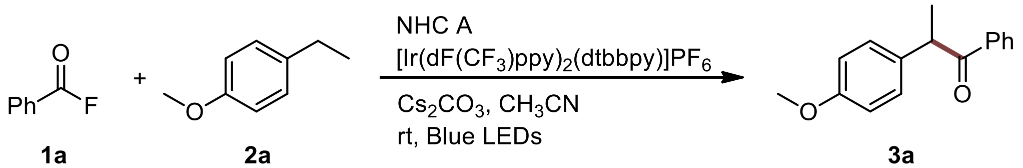

Entry	**1a** (mmol)	NHC A (mmol)	Photocatalyst (mmol)	Cs_2CO_3 (mmol)	Conversion (%)[b]	Product (%)[c]
1	0.3	0.02	0.002	0.2	63	63
2	0.4	0.02	0.001	0.2	76	73
3	0.4	0.01	0.002	0.2	63	61
4	0.4	0.02	0.002	0.15	60	59

[a] Unless otherwise noted, all the reactions were carried out with benzoyl fluoride, 4-ethyl anisole (0.1 mmol), NHC catalyst A, Cs_2CO_3, and $[Ir(dF(CF_3)ppy)_2(dtbbpy)]PF_6$ in anhydrous CH_3CN (2 mL), irradiation with blue LEDs at room temperature for 24 h. [b] GC-FID conversion using 1,3,5-trimethoxybenzene as an internal standard. [c] ^1H NMR yield using 1,3,5-trimethoxybenzene as an internal standard. NHC, N-heterocyclic carbene.

Synthesis of substrates and characterization data

Benzoyl substrates: **1a** and **1k** are commercially available. The characterization data for substrates **1b**, **1c**, **1d**, **1e**, **1f**, **1g**, **1h**, **1i**, **1j**, **1l**, **1m**, **1o**, **1p**, **1q** have been reported in our previous work.[5]

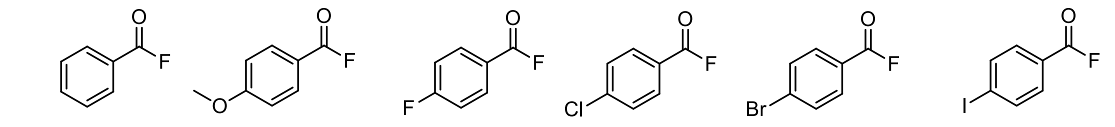

1a **1b** **1c** **1d** **1e** **1f**

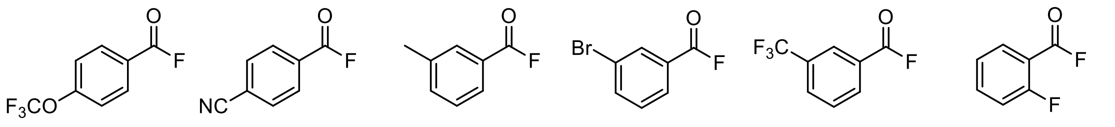

1g **1h** **1i** **1j** **1k** **1l**

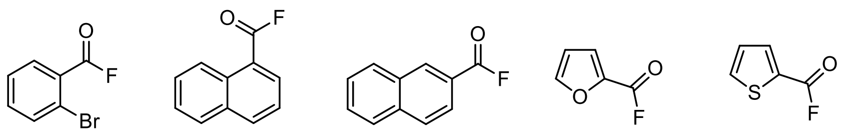

1m **1n** **1o** **1p** **1q**

1-Naphthoyl fluoride (1n)

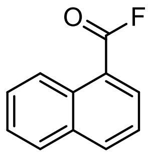

Following a reported procedure,[6] a 100-mL Schlenk tube with a magnetic stir bar was charged with 1-naphthoyl chloride (1.90 g, 10 mmol), 18-crown-6 (132 mg, 0.5 mmol), KF (5.8 g, 100 mmol) and THF (50 mL), stirring for 24 h. After purification by bulb-to-bulb distillation, 1-naphthoyl fluoride was obtained as a white solid (818 mg, 47% yield). ^{1}H NMR (300 MHz, $CDCl_3$) δ (ppm) 9.02 (dt, *J* = 8.7, 1.0 Hz, 1H), 8.36 (dd, *J* = 7.4, 1.3 Hz, 1H), 8.18 (dt, *J* = 8.2, 1.0 Hz, 1H), 7.94 (dd, *J* = 8.1, 1.4 Hz, 1H), 7.72 (ddd, *J* = 8.6, 6.9, 1.5 Hz, 1H), 7.67 – 7.49 (m, 2H). ^{13}C NMR (75 MHz, $CDCl_3$) δ (ppm) 156.5 (d, $J_{C\text{-}F}$ = 342.3 Hz), 136.7, 133.9 (d, $J_{C\text{-}F}$ = 4.0 Hz), 133.7 (d, $J_{C\text{-}F}$ = 2.1 Hz), 132.2 (d, $J_{C\text{-}F}$ = 7.2 Hz), 129.3, 129.1, 127.1, 125.3 (d, $J_{C\text{-}F}$ = 1.5 Hz), 124.6, 120.5 (d, $J_{C\text{-}F}$ = 55.6 Hz). Spectroscopic data are in accordance with those described in literature.[7]

Arene substrates: **2d**, **2f**, **2g**, **2j**, **2k**, **2l**, **2p** and **2s** are commercially available. Other substrates were synthesized according to the reported procedures, as shown below.

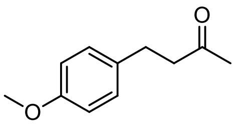

2d

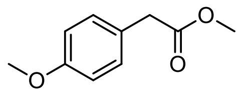

2f

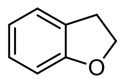

2g

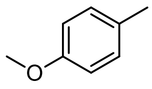

2j

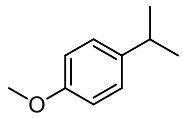

2k

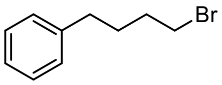

2l

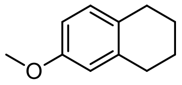

2p

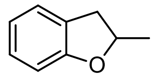

2s

3-(4-Methoxyphenyl)propyl acetate (2a)

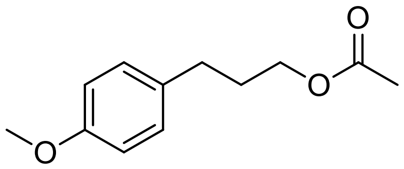

Following a reported procedure,[8] in a flame-dried 100 mL round-bottom flask at room temperature under nitrogen, 3-(4-methoxyphenyl)propan-1-ol (1.66 g, 10 mmol), pyridine (12 mL), 4-dimethylaminopyridine (61 mg, 0.5 mmol) and acetic anhydride (2.04 g, 20 mmol) were added. The reaction was stirred overnight at room temperature then slowly quenched with $NaHCO_3$ (sat) until bubbling ceased. The mixture was extracted with Et_2O (3 x 50 mL). The organic extracts were combined and washed with $CuSO_4$ (sat) (3 x 30 mL), H_2O (2 x 50 mL) then brine (1 x 50 mL). The organic layer was dried (anhydrous Na_2SO_4), filtered and the solvent removed using rotary evaporation. Purification of the desired compound was performed by flash chromatography. The desired product was obtained as a colorless oil (1.93, 93% yield). ^{1}H NMR (300 MHz, $CDCl_3$) δ (ppm) 7.15 – 7.00 (m, 2H), 6.91 – 6.72 (m, 2H), 4.07 (t, J = 6.6 Hz, 2H), 3.78 (s, 3H), 2.63 (dd, J = 8.6, 6.8 Hz, 2H), 2.05 (s, 3H), 1.98 – 1.84 (m, 2H). ^{13}C NMR (75 MHz, $CDCl_3$) (ppm) 171.2, 158.0, 133.3, 129.3, 113.9, 63.9, 55.3, 31.3, 30.5, 21.0. Spectroscopic data are in accordance with those described in literature.[9]

4-(4-Methoxyphenyl)-1-(pyrrolidin-1-yl)butan-1-one (2b)

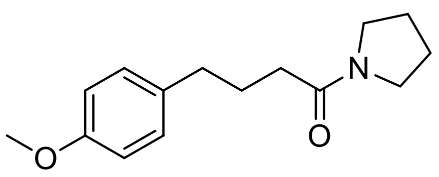

Following a reported procedure,[10] to a solution of the amine (355 mg, 5mmol), triethylamine (502 mg, 5 mmol), hydroxybenzotriazole (HOBt, 675 mg, 5 mmol) and 1-ethyl-3-(3-dimethylaminopropyl)carbodiimide hydrochloride (EDCI*HCl, 960 mg, 5mmol) in DCM (50 mL), 4-(4-methoxyphenyl)butanoic acid (970 mg, 5 mmol) were added and the resulting

solution was stirred at room temperature overnight (14 h). Then, the organic solution was extracted sequentially with 0.5 M aqueous hydrochloric acid (50 mL), saturated aqueous sodium bicarbonate and brine. The washed solution was dried over anhydrous Na_2SO_4, filtered and concentrated under reduced pressure. The resulting crude material was purified by flash column chromatography on silica gel (pentane/ethyl acetate) to afford the desired compound as a colorless oil (1.14 g, 92% yield). ^{1}H NMR (300 MHz, $CDCl_3$) δ (ppm) 7.16 – 6.96 (m, 2H), 6.91 – 6.63 (m, 2H), 3.75 (s, 3H), 3.42 (t, *J* = 6.7 Hz, 2H), 3.29 (t, *J* = 6.6 Hz, 2H), 2.59 (t, *J* = 7.5 Hz, 2H), 2.22 (t, *J* = 7.5 Hz, 2H), 2.01 – 1.66 (m, 6H). ^{13}C NMR (75 MHz, $CDCl_3$) (ppm) 171.4, 157.8, 133.9, 129.4, 113.7, 55.2, 46.5, 45.6, 34.4, 33.8, 26.5, 26.1, 24.4. HRMS (ESI) Calcd. for $C_{15}H_{21}NNaO_2$ $[M+Na]^+$: 270.1470. Found: 270.1462. IR (neat, cm^{-1}): 1634, 1511, 1431, 1342, 1300, 1243, 1176, 1108, 1033, 815, 750.

1-(3-Azidopropyl)-4-methoxybenzene (2c)

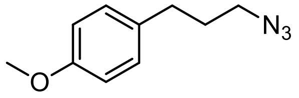

Step 1: following a reported procedure,[11] to a suspension of lithium aluminium hydride (2.85 g, 75 mmol) in dry diethyl ether (110 mL) at 0 °C, 3-(4-methoxyphenyl)propanoic acid (5.41 g, 30 mmol) was added in small portions. The reaction mixture was stirred at room temperature for 5 h, 1 M potassium hydroxide solution (10 mL) and water (15 mL) were then added slowly. The precipitate formed was removed by filtration and washed with diethyl ether. The solvent was removed under reduced pressure. Ethyl acetate (10 mL) was added, the organic phase was separated and dried over $MgSO_4$. The solvent was removed under reduced pressure and the product 3-(4-methoxyphenyl)propan-1-ol was obtained as a colourless liquid (4.37 g, 88% yield). ^{1}H NMR (300 MHz, $CDCl_3$): δ (ppm) 7.16 – 7.07 (m, 2H), 6.89 – 6.79 (m, 2H), 3.79 (s, 3H), 3.67 (h, *J* = 3.8 Hz, 2H), 2.65 (dd, *J* = 8.6, 6.7 Hz, 2H), 1.93 – 1.80 (m, 2H), 1.44 (s, 1H). ^{13}C NMR (76 MHz, $CDCl_3$) δ (ppm) 157.9, 134.0, 129.4, 113.9, 62.4, 55.4, 34.6, 31.3.

Step 2: following a reported procedure,[12] pyridine (1.5 mL) was added to a cooled solution (0 °C) of 3-(4-methoxyphenyl)propan-1-ol (**60**) (830 mg, 5.0 mmol) and methanesulfonyl chloride (0.503 mL, 6.50 mmol). Then, dry THF (7 mL) was added. The reaction mixture was stirred at room temperature for 22 h. After this time, the solvent was removed under reduced pressure, the residue was quenched with 1 M HCl (10 mL) and extracted with ethyl acetate (20 mL). The organic fraction was washed with saturated aqueous $NaHCO_3$ solution (20 mL) and water (20 mL) and dried over $MgSO_4$. The solvent was removed under reduced pressure and the product 3-(4-methoxyphenyl)propyl methanesulfonate was obtained as an orange liquid (927 mg, 76% yield). ^{1}H NMR (300 MHz, $CDCl_3$) δ (ppm) 7.19 – 6.97 (m, 2H), 6.96 – 6.64 (m, 2H), 4.22 (t, *J* = 6.3 Hz, 2H), 3.79 (s, 3H), 2.99 (s, 3H), 2.69 (dd, *J* = 8.2, 6.8 Hz, 2H), 2.16 – 1.92 (m, 2H). ^{13}C NMR (75 MHz, $CDCl_3$) δ (ppm) 158.2, 132.4, 129.5, 114.1, 69.3, 55.4, 37.5, 31.0, 30.7.

Step 3: following a reported procedure,[12] 3-(4-methoxyphenyl)propyl methanesulfonate (185 mg; 0.756 mmol) was dissolved in dry DMF (3 mL), then sodium azide (61.4 mg, 0.945 mmol) was added. The reaction mixture was stirred at room temperature for 21 h. After this time, the mixture was quenched with water (20 mL) and the solution was extracted with diethylether (3 x 20 mL). The combined organic phases were washed with brine (30 mL) and dried over $MgSO_4$. The solvent was removed under reduced pressure and the crude product was purified by column chromatography (pentane/ethyl acetate = 10/1). The desired product was obtained as a yellow oil (110 mg, 76% yield). ^{1}H NMR (300 MHz, $CDCl_3$) δ (ppm) 7.15 – 7.07 (m, 2H), 6.88 – 6.80 (m, 2H), 3.80 (s, 3H), 3.28 (t, *J* = 6.8 Hz, 2H), 2.65 (dd, *J* = 8.3, 6.8 Hz, 2H), 1.95 – 1.82 (m, 2H). ^{13}C NMR (75 MHz, $CDCl_3$) δ (ppm) 158.1, 133.0, 129.5, 114.0, 55.4, 50.7, 32.0, 30.8. Spectroscopic data are in accordance with those described in literature.[12]

1-Methoxy-4-(2-methoxyethyl)benzene (2e)

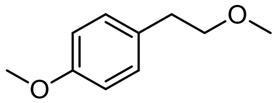

Following a reported procedure,[13] 2-(4-methoxyphenyl)ethan-1-ol (761 mg, 5.0 mmol) was dissolved in dry THF (40 mL), then methyl iodide (467 µL, 7.5 mmol) and a dispersion of sodium hydride in paraffin oil (260 mg, 6.5 mmol) were added. The resulting solution was stirred at 55 °C for 2 h. The reaction solution was allowed to cool to room temperature and then quenched with ice. The aqueous layer was separated and back-extracted with diethylether. The combined organic phases were dried over $MgSO_4$ and the solvent was removed under reduced pressure. The crude product was purified via column chromatography (pentane/ethyl acetate = 10/1) and the desired product was obtained as a yellow liquid (511 mg, 61% yield). ^{1}H NMR (300 MHz, $CDCl_3$) δ (ppm) 7.19 – 7.11 (m, 2H), 6.89 – 6.81 (m, 2H), 3.79 (s, 3H), 3.58 (t, *J* = 7.1 Hz, 2H), 3.36 (s, 3H), 2.84 (t, *J* = 7.1 Hz, 2H). ^{13}C NMR (75 MHz, $CDCl_3$) δ (ppm) 158.2, 131.1, 129.9, 113.9, 74.0, 58.8, 55.3, 35.4. Spectroscopic data are in accordance with those described in literature.[14]

Chromane (2h)

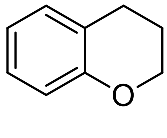

Following a reported procedure,[15] a 100 mL crimp vial was charged with chroman-4-one (740 mg, 5.00 mmol), palladium on charcoal (106.4 mg, 5 wt%, 50 µmol Pd, 1 mol%), 15 mL methanol and chlorobenzene (25.4 µL, 250 µmol, 5mol%). To the sealed vial, polymethylhydrosiloxane (1.5 mL, 25.0 mmol, 5 equiv.) was added via syringe pump over the course of 1 h at room temperature. The mixture was stirred for another hour, filtered, using ethyl acetate to wash both vial and filter. The

solvent was removed under reduced pressure and the crude product was subjected to column chromatography (pentane/ether = 100/0 to 95/5) to obtain the title compound as a colorless oil (596 mg, 89% yield). ^{1}H NMR (300 MHz, $CDCl_3$) δ (ppm) 7.20 – 7.02 (m, 2H), 6.99 – 6.62 (m, 2H), 4.36 – 4.10 (m, 2H), 2.83 (t, *J* = 6.5 Hz, 2H), 2.14 – 1.85 (m, 2H). ^{13}C NMR (75 MHz, $CDCl_3$) δ (ppm) 155.0, 129.9, 127.3, 122.3, 120.2, 116.8, 66.5, 25.0, 22.5. Spectroscopic data are in accordance with those described in literature.[16]

1-Ethyl-4-phenoxybenzene (2i)

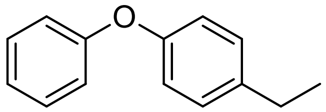

Following a reported procedure,[17] to an oven-dried 100 mL Schlenk flask CuI (190.5 mg, 1 mmol), 2-picolinic acid (307.5 mg, 2.5 mmol), iodobenzene (2.04 g, 10 mmol), 4-ethylphenol (1.59 g, 13 mmol), K_3PO_4 (4.24 g, 20 mmol), and anhydrous DMSO (12 mL) were added. The reaction was heated at 100 ℃ for 24 h under N_2. The reaction mixture was cooled to room temperature and diluted with dichloromethane (DCM) (70 mL) and transferred to a separatory funnel (250 mL). The organic mixture was washed with saturated NH_4Cl (aq) (3 x 50 mL). The organic layer was collected, and dried over anhydrous Na_2SO_4. The Na_2SO_4 was filtered off on a glass frit. All volatiles were removed from the filtrate. The resulting residue was purified by column chromatography on silica gel to afford the pure product as a colorless oil (1.13 g, 57% yield). ^{1}H NMR (300 MHz, $CDCl_3$) δ (ppm) 7.42 – 7.31 (m, 2H), 7.25 – 7.17 (m, 2H), 7.16 – 7.08 (m, 1H), 7.08 – 6.95 (m, 4H), 2.69 (q, *J* = 7.6 Hz, 2H), 1.30 (t, *J* = 7.6 Hz, 3H). ^{13}C NMR (75 MHz, $CDCl_3$) δ (ppm) 157.9, 155.0, 139.4, 129.8, 129.2, 122.9, 119.2, 118.6, 28.3, 15.9. Spectroscopic data are in accordance with those described in literature.[17]

1-(Benzyloxy)-4-ethylbenzene (2m)

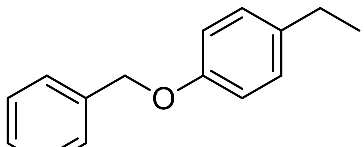

Following a reported procedure,[18] a mixture of 4-ethylphenol (1.22 g, 10 mmol), benzyl bromide (1.88 g, 11 mmol), and $CsCO_3$ (3.58 g, 11 mmol) was dissolved in CH_3CN (25 mL). The mixture was stirred at room temperature for 3 h under a nitrogen atmosphere. After 3 h, the reaction mixture was diluted with CH_2Cl_2. The organic solution was washed with water and brine, dried over anhydrous sodium sulfate, filtered, and concentrated under reduced pressure. The resulting residue was purified by column chromatography on silica gel (pentane) to afford the pure product as a colorless oil (1.38 g, 65% yield). ^{1}H NMR (300 MHz, $CDCl_3$) δ (ppm) 7.54 – 7.34 (m, 5H), 7.22 – 7.12 (m, 2H), 7.02 – 6.89 (m, 2H), 5.10 (s, 2H), 2.66 (q, *J* = 7.6 Hz, 2H), 1.28 (t, *J* = 7.6 Hz,

3H). ^{13}C NMR (75 MHz, $CDCl_3$) (ppm) 157.0, 137.4, 136.8, 128.8, 128.7, 128.0, 127.6, 114.8, 70.2, 28.1, 16.0. Spectroscopic data are in accordance with those described in literature.[19]

5-Methoxy-2,3-dihydro-1H-indene (2o)

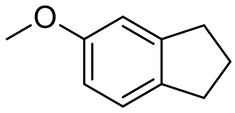

Following a reported procedure,[20] to a solution of 1.34 g (10 mmol) of 2,3-dihydro-1H-inden-5-ol in 10 mL of DMF, 965 μL (15.5 mmol) of MeI and 2.29 g (16.6 mmol) of anhydrous K_2CO_3 were added. The resulting solution was stirred at 55 °C for 4 h under a nitrogen atmosphere. The mixture was cooled to room temperature, diluted with 14 mL of ether and 30 mL of water and extracted with ether. The organic layers were washed with 5% aqueous NaOH, dried over K_2CO_3 and concentrated. The methyl ether was obtained as an orange oil (1.38 g, 93% yield). ^{1}H NMR (300 MHz, $CDCl_3$) δ (ppm) 7.17 (ddt, J = 8.7, 1.1, 0.6 Hz, 1H), 6.85 (dd, J = 2.2, 1.1 Hz, 1H), 6.75 (ddd, J = 8.3, 2.1, 0.8 Hz, 1H), 3.83 (s, 3H), 2.91 (dt, J = 12.3, 7.4 Hz, 4H), 2.27 – 1.93 (m, 2H). ^{13}C NMR (75 MHz, $CDCl_3$) δ (ppm) 158.6, 145.8, 136.2, 124.8, 112.0, 110.0, 55.5, 33.3, 32.1, 26.0. Spectroscopic data are in accordance with those described in literature.[15]

5-Methoxy-1,2,3,4-tetrahydronaphthalene (2q)

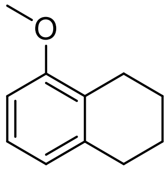

Following a reported procedure,[15] a 100 mL crimp vial was charged with 5-methoxy-3,4-dihydronaphthalen-1(2H)-one (880 mg, 5.00 mmol), palladium on charcoal (106.4 mg, 5 wt%, 50 μmol Pd, 1 mol%), 15 mL methanol and chlorobenzene (25.4 μL, 250 μmol, 5mol%). To the sealed vial, polymethylhydrosiloxane (1.5 mL, 25.0 mmol, 5 equiv.) was added via syringe pump over the course of 1 h at room temperature. The mixture was stirred for another hour, filtered, using ethyl acetate to wash both vial and filter. The solvent was removed under reduced pressure and the crude product was subjected to column chromatography (pentane/ether = 100/0 to 95/5) to obtain the title compound as a colorless oil (705 mg, 87% yield). ^{1}H NMR (300 MHz, $CDCl_3$) δ (ppm) 7.12 (t, J = 7.8 Hz, 1H), 6.72 (dd, J = 16.0, 7.9 Hz, 2H), 3.86 (s, 3H), 2.87 – 2.76 (m, 2H), 2.76 – 2.62 (m, 2H), 1.95 – 1.70 (m, 4H). ^{13}C NMR (75 MHz, $CDCl_3$) δ (ppm) 157.5, 138.6, 126.0, 125.8, 121.5, 106.8, 55.3, 29.8, 23.2, 23.0. Spectroscopic data are in accordance with those described in literature.[21]

5,6-Dimethoxy-2,3-dihydro-1H-indene (2r)

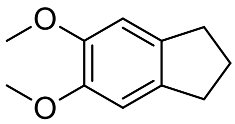

Following a reported procedure,[15] a 100 mL crimp vial was charged with 5,6-dimethoxy-2,3-dihydro-1H-inden-1-one (960 mg, 5.00 mmol), palladium on charcoal (106.4 mg, 5 wt%, 50 µmol Pd, 1 mol%), 15 mL methanol and chlorobenzene (25.4 µL, 250 µmol, 5mol%). To the sealed vial, polymethylhydrosiloxane (1.5 mL, 25.0 mmol, 5 equiv.) was added via syringe pump over the course of 1 h at room temperature. The mixture was stirred for another hour, filtered, using ethyl acetate to wash both vial and filter. The solvent was removed under reduced pressure and the crude product was subjected to column chromatography (pentane/ether = 100/0 to 90/10) to obtain the title compound as a white solid (757 mg, 85% yield). ^{1}H NMR (300 MHz, $CDCl_3$) δ (ppm) 6.68 (s, 2H), 3.76 (s, 6H), 2.76 (t, J = 7.4 Hz, 4H), 1.98 (tt, J = 7.9, 6.9 Hz, 2H). ^{13}C NMR (75 MHz, $CDCl_3$) δ (ppm) 147.8, 135.7, 107.8, 56.1, 32.9, 25.8. HRMS (ESI) Calcd. for $C_{11}H_{14}NaO_2$ $[M+Na]^+$: 201.0891. Found: 201.0885. Spectroscopic data are in accordance with those described in literature.[22]

3-Methylchroman (2t)

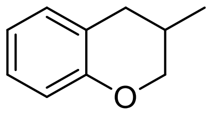

Step 1: following a reported procedure,[23] 1M $LiN(SiMe_3)_2$ solution in THF (13.0 mL, 13.0 mmol) was added to a solution of α-tetralone (1.48 g, 10.0 mmol) in THF (15 mL) and DMI (7.5 mL) at –40 °C and the reaction mixture was stirred for 2 h at the same temperature. MeI (882 µL, 14.0 mmol) was added to the mixture at –40 °C. After stirring over night at rt, the reaction was quenched with 10 % NH_4Cl aq. (35 mL). THF was removed in vacuo, the mixture was extracted with toluene (2 x 30 mL). The combined organic layer was washed with H_2O (40 mL) and 20% NaCl aq. (30 mL). The organic layer was dried over Na_2SO_4 and then concentrated. The residue was purified by silica gel column chromatography to give 3-methylchroman-4-one as a colorless oil (859 mg, 53% yield). ^{1}H NMR (300 MHz, $CDCl_3$) δ (ppm) 7.88 (ddd, J = 7.9, 1.8, 0.5 Hz, 1H), 7.44 (ddd, J = 8.3, 7.2, 1.8 Hz, 1H), 7.11 – 6.72 (m, 2H), 4.48 (dd, J = 11.3, 5.1 Hz, 1H), 4.13 (t, J = 11.2 Hz, 1H), 2.85 (dqd, J = 11.0, 7.0, 5.1 Hz, 1H), 1.20 (d, J = 7.0 Hz, 3H). ^{13}C NMR (75 MHz, $CDCl_3$) δ (ppm) 194.9, 161.8, 135.8, 127.4, 121.4, 120.6, 117.8, 72.3, 40.8, 10.8. Spectroscopic data are in accordance with those described in literature.[23]

Step 2: following a reported procedure,[15] a 100 mL crimp vial was charged with 3-methylchroman-4-one (810 mg, 5.00 mmol), palladium on charcoal (106.4 mg, 5 wt%, 50 µmol Pd, 1 mol%), 15 mL methanol and chlorobenzene (25.4 µL, 250 µmol, 5mol%). To the sealed vial, polymethylhydrosiloxane (1.5 mL, 25.0 mmol, 5 equiv.) was added via syringe pump over the course of 1 h at room temperature. The mixture was stirred for another hour, filtered, using ethyl

acetate to wash both vial and filter. The solvent was removed under reduced pressure and the crude product was subjected to column chromatography (pentane/ether = 100/0 to 95/5) to obtain the title compound as a colorless oil (666 mg, 90% yield). ^{1}H NMR (300 MHz, $CDCl_3$) δ (ppm) 7.20 – 7.01 (m, 2H), 6.96 – 6.73 (m, 2H), 4.19 (ddd, *J* = 10.5, 3.4, 2.0 Hz, 1H), 3.70 (dd, *J* = 10.6, 9.5 Hz, 1H), 2.94 – 2.73 (m, 1H), 2.46 (ddt, *J* = 16.1, 9.7, 1.0 Hz, 1H), 2.17 (ddddt, *J* = 15.1, 8.5, 6.6, 3.3, 1.8 Hz, 1H), 1.07 (d, *J* = 6.7 Hz, 3H). ^{13}C NMR (75 MHz, $CDCl_3$) δ (ppm) 154.5, 129.9, 127.3, 122.0, 120.3, 116.5, 71.9, 33.4, 27.2, 17.2. Spectroscopic data are in accordance with those described in literature.[24]

δ-Tocopherol methyl ether (2u)

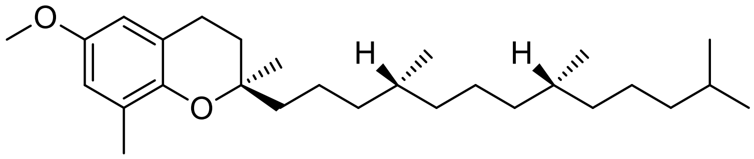

Following a reported procedure,[25] δ-tocopherol (806 mg, 2.0 mmol) was dissolved in DMF (10 mL). K_2CO_3 (552 mg, 4.0 mmol) followed by MeI (568 mg, 4 mmol) was added. After stirring at ambient temperature for 16 h, the reaction mixture was diluted with diethylether (80 mL) and washed with a saturated solution of $NaHCO_3$ (20 mL). The ether layer was dried over anhydrous Na_2SO_4. Na_2SO_4 was removed by filtration, and the ether layer was concentrated. The crude material was purified by flash column chromatography to give the desired product as a light yellow oil (740 mg, 89% yield). ^{1}H NMR (300 MHz, $CDCl_3$) δ (ppm) 6.58 (d, *J* = 3.0 Hz, 1H), 6.45 (d, *J* = 3.1 Hz, 1H), 3.74 (s, 3H), 2.73 (t, *J* = 6.8 Hz, 2H), 2.16 (s, 3H), 1.86 – 1.69 (m, 2H), 1.59 – 1.27 (m, 12H), 1.26 (s, 3H), 1.25 – 1.00 (m, 9H), 0.93 – 0.82 (m, 12H). ^{13}C NMR (75 MHz, $CDCl_3$) δ (ppm) 152.2, 146.3, 127.3, 121.1, 114.9, 111.1, 75.7, 55.8, 40.1, 39.5, 37.6, 37.4, 33.0, 32.8, 31.5, 28.1, 25.0, 24.6, 24.3, 22.9, 22.8, 21.1, 19.9, 19.8, 16.4. Spectroscopic data are in accordance with those described in literature.[25]

(3R,5S,8R,9S,10S,13S,14S)-3-(4-Ethylphenoxy)-10,13-dimethyltetradecahydro-1H-cyclopenta[a]phenanthren-17(2H)-one (2v)

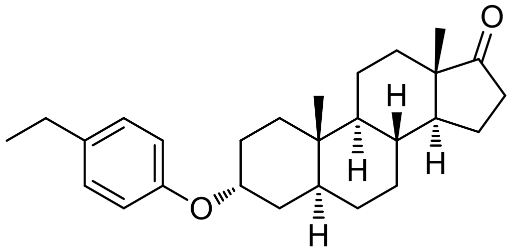

Following a reported procedure,[26] to a solution of epiandrosterone (1.0 g, 3.44 mmol), PPh_3 (1.80 g, 6.89 mmol) and 4-ethylphenol (756 mg, 6.20 mmol) in THF (30 mL), DIAD (diisopropyl

azodicarboxylate, 1.39 g, 6.89 mmol, 2.0 equiv) was added dropwise with cooling in an ice bath and the mixture was allowed to warm to room temperature. After stirring overnight, water was added, and the organic layer was separated. The aqueous layer was extracted with EtOAc twice, and the combined organic layers were washed with brine, and dried over $MgSO_4$. The solvent was removed under reduced pressure, and the residue was purified by flash column chromatography (silica gel, pentane/EtOAc, 10/1) to afford the title compound as a white solid (501 mg, 37%), m.p. = 103-105 ℃. ^{1}H NMR (400 MHz, $CDCl_3$) δ (ppm) 7.15 – 7.05 (m, 2H), 6.87 – 6.75 (m, 2H), 4.49 (p, J = 2.8 Hz, 1H), 2.58 (q, J = 7.6 Hz, 2H), 2.44 (ddd, J = 19.1, 8.9, 1.0 Hz, 1H), 2.07 (dt, J = 19.1, 9.0 Hz, 1H), 1.98 – 1.85 (m, 2H), 1.83 – 1.75 (m, 2H), 1.72 – 1.61 (m, 4H), 1.60 – 1.37 (m, 5H), 1.35 – 1.18 (m, 8H), 1.08 – 0.96 (m, 1H), 0.86 (d, J = 7.3 Hz, 7H). ^{13}C NMR (100 MHz, $CDCl_3$) δ (ppm) 221.6 (C), 155.8 (C), 136.3 (C), 128.8 (CH), 116.2 (CH), 72.1 (CH), 54.4 (CH), 51.6 (CH), 48.0 (C), 39.7 (CH), 36.1 (C), 36.0 (CH_2), 35.2 (CH), 32.9 (CH_2), 32.8 (CH_2), 31.7 (CH_2), 30.9 (CH_2), 28.3 (CH_2), 28.1 (CH_2), 25.7 (CH_2), 21.9 (CH_2), 20.2 (CH_2), 15.9 (CH_3), 14.0 (CH_3), 11.6 (CH_3). HRMS (ESI) Calcd. for $C_{27}H_{38}NaO_2$ $[M+Na]^+$: 417.2770. Found: 417.2756. IR (neat, cm^{-1}): 1738, 1611, 1451, 1371, 1293, 1235, 1167, 1119, 1058, 995, 828.

tert-Butyl 3,4-dimethoxyphenethylcarbamate (2w)

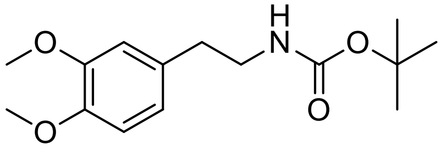

Following a reported procedure,[27] to a solution of 2-(3,4-dimethoxyphenyl)ethan-1-amine (905 mg, 5 mmol) in DCM (10 mL), triethylamine (1.01 g, 10 mmol) was added and the mixture was stirred for 15 min. at room temperature. After that, Boc_2O (1.20 g, 5.5 mmol) was added. The mixture was stirred at room temperature for 20 h. Then the reaction mixture was directly concentrated under reduced pressure. The residue was purified by flash column chromatography (ethyl acetate/ petroleum ether) on silica gel to give the title compound as a white solid (1.98 g, 88% yield). ^{1}H NMR (300 MHz, $CDCl_3$) δ (ppm) 6.86 – 6.76 (m, 1H), 6.76 – 6.63 (m, 2H), 4.58 (s, 1H), 3.85 (s, 3H), 3.84 (s, 3H), 3.33 (d, J = 6.2 Hz, 2H), 2.72 (t, J = 7.1 Hz, 2H), 1.42 (s, 9H). ^{13}C NMR (75 MHz, $CDCl_3$) δ (ppm) 156.0, 149.0, 147.6, 131.6, 120.7, 112.0, 111.4, 79.3, 56.0, 55.9, 42.0, 35.8, 28.5. Spectroscopic data are in accordance with those described in literature.[27]

4,4-d_2-Chromane (2h-d_2)

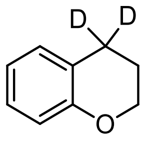

Following a reported procedure,[28] a flame-dried vial was charged with water free $AlCl_3$ (472 mg,

3.54 mmol) and lithium aluminum deuteride (84 mg, 2.00 mmol) under nitrogen atmosphere. The mixture was carefully suspended in dry ether (8 mL). Chroman-4-one (296 mg, 2.0 mmol) was carefully added as solid (violent reaction) to the suspension. The mixture was stirred for 1 h at room temperature, diluted with ether (20 mL) and quenched by the addition of aqueous HCl (1 M). The phases where separated, and the aqueous phase was extracted with ether (3 x 10 mL). The organic phase was dried over sodium sulfate, filtered, and concentrated. The solvent was removed under reduced pressure and the crude product was submitted to column chromatography (pentane/ether = 100/0 to 90/10) to obtain the title compound as a colorless oil (234 mg, 85% yield). ^{1}H NMR (300 MHz, $CDCl_3$) δ (ppm) 7.18 – 7.01 (m, 2H), 6.84 (ddt, J = 15.2, 7.9, 1.3 Hz, 2H), 4.28 – 4.08 (m, 2H), 2.01 (t, J = 5.2 Hz, 2H). ^{13}C NMR (75 MHz, $CDCl_3$) δ (ppm) 155.0 (C), 129.9 (CH), 127.3 (CH), 122.2 (C), 120.2 (CH), 116.8 (CH), 66.5 (CH_2), 24.3 (CD_2), 22.3 (CH_2). HRMS (EI) Calcd. for $C_9H_8D_2O$ $[M]^+$: 136.0857. Found: 136.0851. IR (neat, cm^{-1}): 1608, 1580, 1488, 1452, 1290, 1258, 1219, 1119, 1052, 953, 852, 752.

Characterization data for products

2-(4-Methoxyphenyl)-1-phenylpropan-1-one (3a)

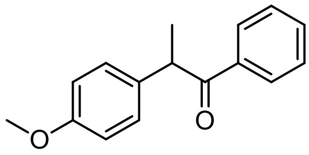

The reaction was performed according to general procedure with 1-ethyl-4-methoxybenzene (13.6 mg, 0.1 mmol) and benzoyl fluoride (49.6 mg, 0.4 mmol). After purification by flash chromatography (*n*-pentane/ethyl acetate = 30/1), the desired compound was obtained as a colorless oil (19.9 mg, 83% yield). ^{1}H NMR (300 MHz, $CDCl_3$) δ (ppm) 8.01 – 7.90 (m, 2H), 7.52 – 7.42 (m, 1H), 7.42 – 7.33 (m, 2H), 7.24 – 7.15 (m, 2H), 6.92 – 6.73 (m, 2H), 4.65 (q, J = 6.9 Hz, 1H), 3.75 (s, 3H), 1.51 (d, J = 6.8 Hz, 3H). ^{13}C NMR (75 MHz, $CDCl_3$) δ (ppm) 200.7, 158.6, 136.6, 133.6, 132.8, 128.9, 128.9, 128.6, 114.5, 55.3, 47.1, 19.6. HRMS (ESI) Calcd. for $C_{16}H_{16}NaO_2$ $[M+Na]^+$: 263.1048. Found: 263.1039. Spectroscopic data are in accordance with those described in literature.[29]

1,2-Bis(4-methoxyphenyl)propan-1-one (3b)

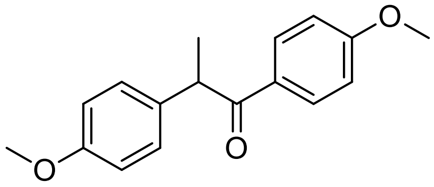

The reaction was performed according to general procedure with 1-ethyl-4-methoxybenzene (13.6 mg, 0.1 mmol) and 4-methoxybenzoyl fluoride (61.7 mg, 0.4 mmol). After purification by flash chromatography (*n*-pentane/ethyl acetate = 40/1 to 20/1), the desired compound was obtained as a brown oil (22.9 mg, 85% yield). ^{1}H NMR (300 MHz, $CDCl_3$) δ (ppm) 8.05 – 7.83 (m, 2H), 7.24 – 7.14 (m, 2H), 7.01 – 6.75 (m, 4H), 4.59 (q, J = 6.9 Hz, 1H), 3.82 (s, 3H), 3.75 (s, 3H), 1.49 (d, J = 6.8 Hz, 3H). ^{13}C NMR (75 MHz, $CDCl_3$) δ (ppm) 199.2, 163.3, 158.5, 134.1, 131.1, 129.6, 128.8, 114.4, 113.7, 55.5, 55.3, 46.7, 19.7. HRMS (ESI) Calcd. for $C_{17}H_{18}NaO_3$ $[M+Na]^+$: 293.1154. Found: 293.1144. Spectroscopic data are in accordance with those described in literature.[30]

1-(4-Fluorophenyl)-2-(4-methoxyphenyl)propan-1-one (3c)

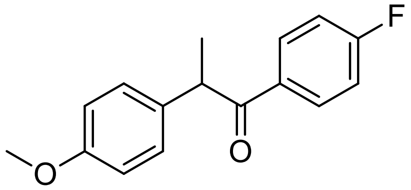

The reaction was performed according to general procedure with 1-ethyl-4-methoxybenzene (13.6 mg, 0.1 mmol) and 4-fluorobenzoyl fluoride (68.5 mg, 0.4 mmol). After purification by flash chromatography (*n*-pentane/ethyl acetate = 40/1 to 20/1), the desired compound was obtained as a yellow oil (24.6 mg, 95% yield). ^{1}H NMR (300 MHz, $CDCl_3$) δ (ppm) 8.02 – 7.92 (m, 2H), 7.23 – 7.13 (m, 2H), 7.07 – 6.99 (m, 2H), 6.86 – 6.81 (m, 2H), 4.58 (q, *J* = 6.8 Hz, 1H), 3.75 (s, 3H), 1.50 (d, *J* = 6.8 Hz, 3H). ^{13}C NMR (75 MHz, $CDCl_3$) δ (ppm) 199.0, 165.5 (d, *J* = 254.5 Hz), 158.7, 133.5, 133.0 (d, *J* = 3.0 Hz), 131.5 (d, *J* = 9.2 Hz), 128.8, 115.7 (d, *J* = 21.8 Hz), 114.6, 55.3, 47.2, 19.6. ^{19}F NMR {^{1}H} (282 MHz, $CDCl_3$): δ (ppm) -105.7. HRMS (ESI) Calcd. for $C_{16}H_{15}FnaO_2$ $[M+Na]^+$: 281.0954. Found: 281.0952. Spectroscopic data are in accordance with those described in literature.[30]

1-(4-Chlorophenyl)-2-(4-methoxyphenyl)propan-1-one (3d)

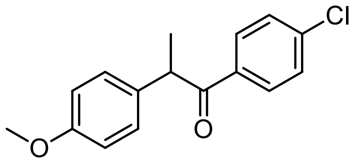

The reaction was performed according to general procedure with 1-ethyl-4-methoxybenzene (13.6 mg, 0.1 mmol) and 4-chlorobenzoyl fluoride (63.2 mg, 0.4 mmol). After purification by flash chromatography (*n*-pentane/ethyl acetate = 30/1), the desired compound was obtained as a colorless oil (24.4 mg, 89% yield). ^{1}H NMR (300 MHz, $CDCl_3$) δ (ppm) 7.92 – 7.82 (m, 2H), 7.39 – 7.28 (m, 2H), 7.20 – 7.11 (m, 2H), 6.87 – 6.75 (m, 2H), 4.57 (q, *J* = 6.8 Hz, 1H), 3.75 (s, 3H), 1.50 (d, *J* = 6.8 Hz, 3H). ^{13}C NMR (75 MHz, $CDCl_3$) δ (ppm) 199.4, 158.7, 139.2, 134.9, 133.3, 130.3, 128.9, 128.8, 114.6, 55.3, 47.3, 19.6. HRMS (ESI) Calcd. for $C_{16}H_{15}ClNaO_2$ $[M+Na]^+$: 297.0658. Found: 297.0652. Spectroscopic data are in accordance with those described in literature.[31]

1-(4-Bromophenyl)-2-(4-methoxyphenyl)propan-1-one (3e)

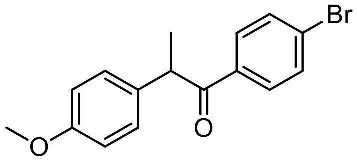

The reaction was performed according to general procedure with 1-ethyl-4-methoxybenzene (13.6 mg, 0.1 mmol) and 4-bromobenzoyl fluoride (80.8 mg, 0.4 mmol). After purification by flash chromatography (*n*-pentane/ethyl acetate = 30/1), the desired compound was obtained as a colorless oil (29.2 mg, 92% yield). ^{1}H NMR (300 MHz, $CDCl_3$) δ (ppm) 7.86 – 7.75 (m, 2H), 7.55 – 7.45 (m, 2H), 7.22 – 7.11 (m, 2H), 6.87 – 6.74 (m, 2H), 4.56 (q, *J* = 6.8 Hz, 1H), 3.75 (s, 3H), 1.50 (d, *J* = 6.8 Hz, 3H). ^{13}C NMR (75 MHz, $CDCl_3$) δ (ppm) 199.5 (C), 158.7 (C), 135.3 (C), 133.2 (C), 131.9 (CH), 130.4 (CH), 128.8 (CH), 127.9 (C), 114.6 (CH), 55.3 (CH_3), 47.3 (CH), 19.5 (CH_3). HRMS (ESI) Calcd. for $C_{16}H_{15}BrNaO_2$ $[M+Na]^+$: 341.0153. Found: 341.0147. IR

(neat, cm^{-1}): 2932, 1680, 1584, 1510, 1396, 1244, 1175, 1070, 1033, 1004, 950, 830, 778, 763.

1-(4-Iodophenyl)-2-(4-methoxyphenyl)propan-1-one (3f)

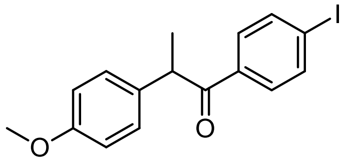

The reaction was performed according to general procedure with 1-ethyl-4-methoxybenzene (13.6 mg, 0.1 mmol) and 4-iodobenzoyl fluoride (100 mg, 0.4 mmol). After purification by flash chromatography (*n*-pentane/ethyl acetate = 30/1), the desired compound was obtained as a colorless oil (17.5 mg, 48% yield). ^{1}H NMR (300 MHz, $CDCl_3$) δ (ppm) 7.77 – 7.68 (m, 2H), 7.68 – 7.57 (m, 2H), 7.20 – 7.08 (m, 2H), 6.87 – 6.74 (m, 2H), 4.55 (q, *J* = 6.8 Hz, 1H), 3.75 (s, 3H), 1.49 (d, *J* = 6.8 Hz, 3H). ^{13}C NMR (75 MHz, $CDCl_3$) δ (ppm) 199.8 (C), 158.7 (C), 137.9 (CH), 135.8 (C), 133.2 (C), 130.3 (CH), 128.8 (CH), 114.6 (CH), 100.8 (C), 55.3 (CH_3), 47.2 (CH), 19.5 (CH_3). HRMS (ESI) Calcd. for $C_{16}H_{15}INaO_2$ $[M+Na]^+$: 389.0014. Found: 389.0009. IR (neat, cm^{-1}): 2930, 1680, 1580, 1510, 1391, 1245, 1177, 1059, 1033, 1002, 950, 830, 776, 762.

2-(4-Methoxyphenyl)-1-(4-(trifluoromethoxy)phenyl)propan-1-one (3g)

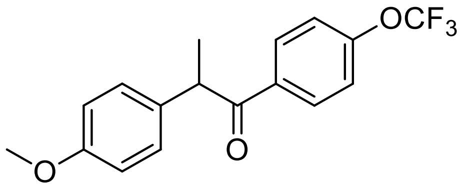

The reaction was performed according to general procedure with 1-ethyl-4-methoxybenzene (13.6 mg, 0.1 mmol) and 4-(trifluoromethoxy)benzoyl fluoride (83.2 mg, 0.4 mmol). After purification by flash chromatography (*n*-pentane/ethyl acetate = 40/1), the desired compound was obtained as a colorless oil (15.9 mg, 49% yield). ^{1}H NMR (300 MHz, $CDCl_3$) δ (ppm) 8.02 – 7.94 (m, 2H), 7.24 – 7.12 (m, 4H), 6.89 – 6.81 (m, 2H), 4.58 (q, *J* = 6.8 Hz, 1H), 3.76 (s, 3H), 1.50 (d, *J* = 6.8 Hz, 3H). ^{13}C NMR (100 MHz, $CDCl_3$) δ (ppm) 199.0 (C), 158.8 (C), 152.4 (C), 134.8 (C), 133.2 (C), 130.9 (CH), 128.9 (CH), 120.4 (q, J_{C-F} = 257.0 Hz, CF_3), 120.4 (CH), 114.7 (CH), 55.3 (CH_3), 47.4 (CH), 19.6 (CH_3). ^{19}F NMR {^{1}H} (282 MHz, $CDCl_3$): δ (ppm) -57.6. HRMS (ESI) Calcd. for $C_{17}H_{15}F_3NaO_3$ $[M+Na]^+$: 347.0871. Found: 347.0863. IR (neat, cm^{-1}): 2958, 2929, 2845, 1686, 1604, 1511, 1374, 1303, 1248, 1210, 1165, 1113, 1036, 1006, 954, 833, 780.

4-(2-(4-Methoxyphenyl)propanoyl)benzonitrile (3h)

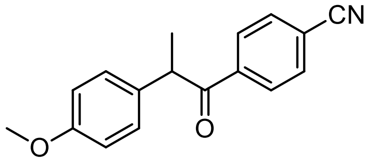

The reaction was performed according to general procedure with 1-ethyl-4-methoxybenzene (13.6 mg, 0.1 mmol) and 4-cyanobenzoyl fluoride (100 mg, 0.4 mmol). After purification by flash chromatography (*n*-pentane/ethyl acetate = 15/1), the desired compound was obtained as a white solid (18.8 mg, 71% yield), m.p. = 101-103 ℃. ^{1}H NMR (300 MHz, $CDCl_3$) δ (ppm) 8.03 – 7.93 (m, 2H), 7.70 – 7.59 (m, 2H), 7.18 – 7.06 (m, 2H), 6.88 – 6.77 (m, 2H), 4.56 (q, *J* = 6.8 Hz, 1H), 3.75 (s, 3H), 1.51 (d, *J* = 6.8 Hz, 3H). ^{13}C NMR (75 MHz, $CDCl_3$) δ (ppm) 199.2 (C), 158.9 (C), 139.7 (C), 132.5 (C), 132.4 (CH), 129.2 (CH), 128.9 (CH), 118.1 (C), 116.0 (C), 114.8 (CH), 55.3 (CH_3), 47.8 (CH), 19.4 (CH_3). HRMS (ESI) Calcd. for $C_{17}H_{15}NNaO_2$ $[M+Na]^+$: 288.1000. Found: 288.0995. IR (neat, cm^{-1}): 2934, 2231, 1687, 1609, 1511, 1248, 1218, 1178, 1033, 953, 858, 833, 776.

2-(4-Methoxyphenyl)-1-(m-tolyl)propan-1-one (3i)

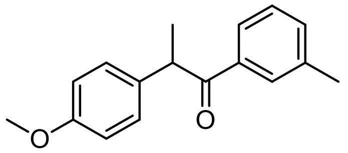

The reaction was performed according to general procedure with 1-ethyl-4-methoxybenzene (13.6 mg, 0.1 mmol) and 3-methylbenzoyl fluoride (55.2 mg, 0.4 mmol). After purification by flash chromatography (*n*-pentane/ethyl acetate = 30/1), the desired compound was obtained as a colorless oil (18.5 mg, 73% yield). ^{1}H NMR (300 MHz, $CDCl_3$) δ (ppm) 7.81 – 7.65 (m, 2H), 7.32 – 7.13 (m, 4H), 6.88 – 6.77 (m, 2H), 4.65 (q, *J* = 6.9 Hz, 1H), 3.75 (s, 3H), 2.36 (s, 3H), 1.51 (d, *J* = 6.9 Hz, 3H). ^{13}C NMR (75 MHz, $CDCl_3$) δ (ppm) 200.9 (C), 158.6 (C), 138.4 (C), 136.7 (C), 133.7 (C), 133.6 (CH), 129.4 (CH), 128.9 (CH), 128.4 (CH), 126.1 (CH), 114.5 (CH), 55.3 (CH_3), 47.0 (CH), 21.5 (CH_3), 19.7 (CH_3). HRMS (ESI) Calcd. for $C_{17}H_{18}NaO_2$ $[M+Na]^+$: 277.1204. Found: 277.1196. IR (neat, cm^{-1}): 2930, 1677, 1608, 1510, 1453, 1302, 1246, 1178, 1160, 1030, 967, 833, 761, 691.

1-(3-Bromophenyl)-2-(4-methoxyphenyl)propan-1-one (3j)

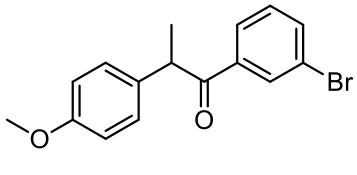

The reaction was performed according to general procedure with 1-ethyl-4-methoxybenzene (13.6 mg, 0.1 mmol) and 3-bromobenzoyl fluoride (80.8 mg, 0.4 mmol). After purification by flash chromatography (*n*-pentane/ethyl acetate = 30/1), the desired compound was obtained as a colorless oil (26.7 mg, 84% yield). ^{1}H NMR (300 MHz, $CDCl_3$) δ (ppm) 8.10 (t, *J* = 1.8 Hz, 1H), 7.86 (ddd, *J* = 7.8, 1.7, 1.0 Hz, 1H), 7.61 (ddd, *J* = 7.9, 2.0, 1.0 Hz, 1H), 7.26 (t, *J* = 7.9 Hz, 1H), 7.22 – 7.12 (m, 2H), 6.89 – 6.79 (m, 2H), 4.59 (q, *J* = 6.8 Hz, 1H), 3.78 (s, 3H), 1.52 (d, *J* = 6.8 Hz, 3H). ^{13}C NMR (75 MHz, $CDCl_3$) δ (ppm) 199.2 (C), 158.7 (C), 138.4 (C), 135.6 (CH), 133.0

(C), 131.9 (CH), 130.1 (CH), 128.9 (CH), 127.4 (CH), 123.0 (C), 114.6 (CH), 55.3 (CH_3), 47.3 (CH), 19.5 (CH_3). HRMS (ESI) Calcd. for $C_{16}H_{15}BrNaO_2$ $[M+Na]^+$: 341.0153. Found: 341.0148. IR (neat, cm^{-1}): 2932, 1684, 1610, 1565, 1510, 1303, 1246, 1178, 1034, 834, 760, 698.

2-(4-Methoxyphenyl)-1-(3-(trifluoromethyl)phenyl)propan-1-one (3k)

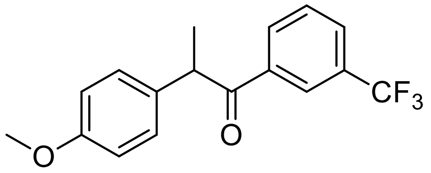

The reaction was performed according to general procedure with 1-ethyl-4-methoxybenzene (13.6 mg, 0.1 mmol) and 3-(trifluoromethyl)benzoyl fluoride (76.8 mg, 0.4 mmol). After purification by flash chromatography (*n*-pentane/ethyl acetate = 40/1), the desired compound was obtained as a yellow oil (29.2 mg, 95% yield). ^1H NMR (300 MHz, $CDCl_3$) δ (ppm) 8.22 (d, J = 1.8 Hz, 1H), 8.09 (d, J = 8.1 Hz, 1H), 7.76 – 7.69 (m, 1H), 7.50 (t, J = 7.8 Hz, 1H), 7.23 – 7.14 (m, 2H), 6.88 – 6.80 (m, 2H), 4.62 (q, J = 6.8 Hz, 1H), 3.75 (s, 3H), 1.53 (d, J = 6.8 Hz, 3H). ^{13}C NMR (100 MHz, $CDCl_3$) δ (ppm) 199.1 (C), 158.8 (C), 137.1 (C), 132.8 (C), 132.0 (CH), 131.2 (q, $J_{C\text{-}F}$ = 32.6 Hz, C), 129.2 (CH), 129.2 (q, $J_{C\text{-}F}$ = 3.5 Hz, CH), 128.9 (CH), 125.7 (q, $J_{C\text{-}F}$ = 3.9 Hz, CH), 123.8 (q, $J_{C\text{-}F}$ = 270.7 Hz, CF_3), 114.7 (CH), 55.3 (CH_3), 47.5 (CH), 19.5 (CH_3). ^{19}F NMR {^1H} (282 MHz, $CDCl_3$): δ (ppm) -62.9. HRMS (ESI) Calcd. for $C_{17}H_{15}F_3NaO_2$ $[M+Na]^+$: 331.0922. Found: 331.0914. IR (neat, cm^{-1}): 2976, 2933, 2839, 1689, 1611, 1511, 1443, 1374, 1325, 1248, 1210, 1167, 1125, 1072, 1034, 963, 925, 835, 772, 751, 693, 649.

1-(2-Fluorophenyl)-2-(4-methoxyphenyl)propan-1-one (3l)

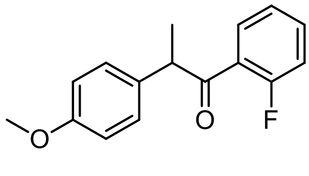

The reaction was performed according to general procedure with 1-ethyl-4-methoxybenzene (13.6 mg, 0.1 mmol) and 2-fluorobenzoyl fluoride (56.8 mg, 0.4 mmol). After purification by flash chromatography (*n*-pentane/ethyl acetate = 30/1), the desired compound was obtained as a colorless oil (21.0 mg, 81% yield). ^1H NMR (300 MHz, $CDCl_3$) δ (ppm) 7.70 (td, J = 7.6, 1.9 Hz, 1H), 7.44 – 7.36 (m, 1H), 7.20 – 7.08 (m, 3H), 7.02 (ddd, J = 11.2, 8.3, 1.1 Hz, 1H), 6.84 – 6.73 (m, 2H), 4.56 (q, J = 6.9 Hz, 1H), 3.74 (s, 3H), 1.51 (dd, J = 6.9, 0.9 Hz, 3H). ^{13}C NMR (75 MHz, $CDCl_3$) δ (ppm) 199.9 (C), 160.8 (C, d, $J_{C\text{-}F}$ = 251.8 Hz), 158.5 (C), 134.0 (CH), 133.8 (C), 132.5 (CH), 131.0 (CH, d, $J_{C\text{-}F}$ = 2.2 Hz), 129.2 (CH), 124.4 (CH, d, $J_{C\text{-}F}$ = 3.4 Hz), 116.5 (C, d, $J_{C\text{-}F}$ = 23.8 Hz), 114.1 (CH), 55.2 (CH_3), 51.0 (CH, d, $J_{C\text{-}F}$ = 6.4 Hz), 18.9 (CH_3). ^{19}F {^1H} NMR (282 MHz, $CDCl_3$) δ (ppm) -110.07. HRMS (ESI) Calcd. for $C_{16}H_{15}FNaO_2$ $[M+Na]^+$: 281.0954. Found: 281.0945. IR (neat, cm^{-1}): 2932, 1682, 1608, 1511, 1480, 1450, 1248, 1210, 1178, 1105, 1033, 953,

828, 755, 563.

1-(2-Bromophenyl)-2-(4-methoxyphenyl)propan-1-one (3m)

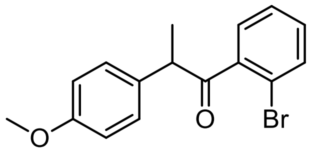

The reaction was performed according to general procedure with 1-ethyl-4-methoxybenzene (13.6 mg, 0.1 mmol) and 2-bromobenzoyl fluoride (80.8 mg, 0.4 mmol). After purification by flash chromatography (*n*-pentane/ethyl acetate = 30/1), the desired compound was obtained as a colorless oil (16.4 mg, 52% yield). ^{1}H NMR (300 MHz, $CDCl_3$) δ (ppm) 7.56 – 7.48 (m, 1H), 7.22 – 7.15 (m, 2H), 7.15 – 7.08 (m, 2H), 6.96 (dd, *J* = 5.9, 3.5 Hz, 1H), 6.84 – 6.74 (m, 2H), 4.43 (q, *J* = 6.9 Hz, 1H), 3.76 (s, 3H), 1.56 (d, *J* = 6.9 Hz, 3H). ^{13}C NMR (75 MHz, $CDCl_3$) δ (ppm) 204.9 (C), 158.9 (C), 142.1 (C), 133.3 (CH), 131.3 (C), 131.0 (CH), 129.5 (CH), 128.7 (CH), 127.1 (CH), 118.6 (C), 114.3 (CH), 55.3 (CH_3), 51.3 (CH), 17.8 (CH_3). HRMS (ESI) Calcd. for $C_{16}H_{15}BrNaO_2$ $[M+Na]^+$: 341.0153. Found: 341.0146. IR (neat, cm^{-1}): 1698, 1609, 1510, 1465, 1428, 1302, 1246, 1178, 1029, 957, 834, 750, 681, 564.

2-(4-Methoxyphenyl)-1-(naphthalen-1-yl)propan-1-one (3n)

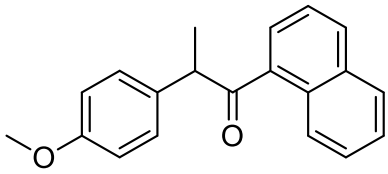

The reaction was performed according to general procedure with 1-ethyl-4-methoxybenzene (13.6 mg, 0.1 mmol) and 1-naphthoyl fluoride (69.6 mg, 0.4 mmol). After purification by flash chromatography (*n*-pentane/ethyl acetate = 30/1), the desired compound was obtained as a yellow oil (13.7 mg, 47% yield). ^{1}H NMR (300 MHz, $CDCl_3$) δ (ppm) 8.31 (dq, *J* = 7.6, 0.8 Hz, 1H), 7.95 – 7.71 (m, 3H), 7.57 – 7.36 (m, 3H), 7.26 – 7.16 (m, 2H), 6.87 – 6.62 (m, 2H), 4.65 (q, *J* = 6.9 Hz, 1H), 3.73 (s, 3H), 1.62 (d, *J* = 6.9 Hz, 3H). ^{13}C NMR (75 MHz, $CDCl_3$) δ (ppm) 205.0 (C), 158.6 (C), 136.9 (C), 134.0 (C), 132.7 (C), 132.0 (CH), 130.6 (C), 129.1 (CH), 128.4 (CH), 127.7 (CH), 126.7 (CH), 126.4 (CH), 125.8 (CH), 124.4 (CH), 114.4 (CH), 55.3 (CH_3), 50.6 (CH), 19.0 (CH_3). HRMS (ESI) Calcd. for $C_{20}H_{18}NaO_2$ $[M+Na]^+$: 313.1204. Found: 313.1196. IR (neat, cm^{-1}): 2929, 1682, 1610, 1510, 1462, 1372, 1302, 1250, 1178, 1105, 1060, 1033, 933, 836, 783, 735.

2-(4-Methoxyphenyl)-1-(naphthalen-2-yl)propan-1-one (3o)

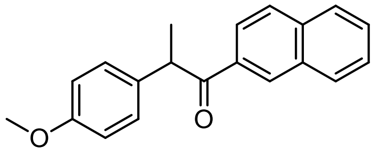

The reaction was performed according to general procedure with 1-ethyl-4-methoxybenzene (13.6 mg, 0.1 mmol) and 2-naphthoyl fluoride (69.6 mg, 0.4 mmol). After purification by flash chromatography (*n*-pentane/ethyl acetate = 30/1), the desired compound was obtained as a white solid (22.0 mg, 76% yield), m.p. = 92-94 ℃. ^{1}H NMR (300 MHz, $CDCl_3$) δ (ppm) 8.55 – 8.47 (m, 1H), 8.05 (dd, *J* = 8.7, 1.8 Hz, 1H), 7.99 – 7.89 (m, 1H), 7.84 (dd, *J* = 8.1, 1.6 Hz, 2H), 7.64 – 7.46 (m, 2H), 7.35 – 7.25 (m, 2H), 6.91 – 6.77 (m, 2H), 4.84 (q, *J* = 6.8 Hz, 1H), 3.76 (s, 3H), 1.61 (d, *J* = 6.8 Hz, 3H). ^{13}C NMR (75 MHz, $CDCl_3$) δ (ppm) 200.6 (C), 158.6 (C), 135.5 (C), 134.0 (C), 133.7 (C), 132.6 (C), 130.5 (CH), 129.7 (CH), 128.9 (CH), 128.44 (CH), 128.39 (CH), 127.8 (CH), 126.7 (CH), 124.7 (CH), 114.5 (CH), 55.3 (CH_3), 47.1 (CH), 19.7 (CH_3). HRMS (ESI) Calcd. for $C_{20}H_{18}NaO_2$ $[M+Na]^+$: 313.1204. Found: 313.1196. IR (neat, cm^{-1}): 2932, 1674, 1510, 1466, 1249, 1178, 1126, 1034, 933, 829, 785.

1-(Furan-2-yl)-2-(4-methoxyphenyl)propan-1-one (3p)

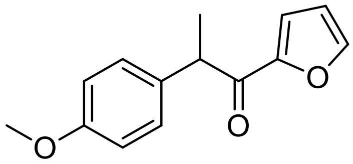

The reaction was performed according to general procedure with 1-ethyl-4-methoxybenzene (13.6 mg, 0.1 mmol) and furan-2-carbonyl fluoride (45.6 mg, 0.4 mmol). After purification by flash chromatography (*n*-pentane/ethyl acetate = 40/1 to 20/1), the desired compound was obtained as a colorless oil (10.8 mg, 47% yield). ^{1}H NMR (300 MHz, $CDCl_3$) δ (ppm) 7.45 (dd, *J* = 1.7, 0.8 Hz, 1H), 7.22 – 7.13 (m, 2H), 7.05 (dd, *J* = 3.6, 0.8 Hz, 1H), 6.81 – 6.72 (m, 2H), 6.38 (dd, *J* = 3.6, 1.7 Hz, 1H), 4.37 (q, *J* = 6.9 Hz, 1H), 3.69 (s, 3H), 1.42 (d, *J* = 7.0 Hz, 3H). ^{13}C NMR (75 MHz, $CDCl_3$) δ (ppm) 189.8 (C), 158.7 (C), 152.3 (C), 146.4 (CH), 133.0 (C), 129.0 (CH), 117.9 (CH), 114.3 (CH), 112.3 (CH), 55.4 (CH_3), 47.2 (CH), 18.4 (CH_3). HRMS (ESI) Calcd. for $C_{14}H_{14}NaO_3$ $[M+Na]^+$: 253.0841. Found: 253.0833. IR (neat, cm^{-1}): 2956, 2926, 2852, 1667, 1611, 1582, 1563, 1511, 1466, 1392, 1375, 1303, 1254, 1178, 1162, 1081, 1029, 1004, 958, 905, 880, 840, 814, 794, 781, 748, 596.

2-(4-Methoxyphenyl)-1-(thiophen-2-yl)propan-1-one (3q)

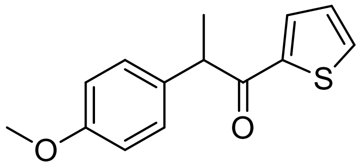

The reaction was performed according to general procedure with 1-ethyl-4-methoxybenzene (13.6 mg, 0.1 mmol) and thiophene-2-carbonyl fluoride (52.0 mg, 0.4 mmol). After purification by flash chromatography (*n*-pentane/ethyl acetate = 40/1 to 20/1), the desired compound was obtained as a colorless oil (20.3 mg, 82% yield). ^{1}H NMR (300 MHz, $CDCl_3$) δ (ppm) 7.67 (dd, *J* = 3.8, 1.1 Hz,

1H), 7.55 (dd, J = 4.9, 1.1 Hz, 1H), 7.27 – 7.22 (m, 2H), 7.04 (dd, J = 5.0, 3.8 Hz, 1H), 6.89 – 6.81 (m, 2H), 4.46 (q, J = 6.9 Hz, 1H), 3.76 (s, 3H), 1.52 (d, J = 6.9 Hz, 3H). ^{13}C NMR (75 MHz, $CDCl_3$) δ (ppm) 193.7 (C), 158.8 (C), 143.8 (C), 133.6 (CH), 133.5 (C), 132.5 (CH), 128.9 (CH), 128.1 (CH), 114.4 (CH), 55.3 (CH_3), 48.6 (CH), 19.3 (CH_3). HRMS (ESI) Calcd. for $C_{14}H_{14}NaO_2S$ $[M+Na]^+$: 269.0612. Found: 269.0604. IR (neat, cm^{-1}): 2965, 2929, 2838, 1657, 1609, 1582, 1510, 1454, 1412, 1371, 1354, 1302, 1244, 1177, 1115, 1054, 1032, 997, 942, 906, 855, 832, 778, 720, 678.

3-(4-Methoxyphenyl)-4-oxo-4-phenylbutyl acetate (3r)

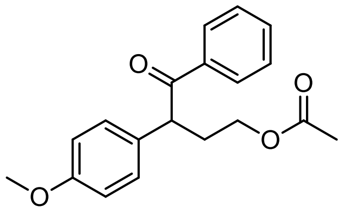

The reaction was performed according to general procedure with 3-(4-methoxyphenyl)propyl acetate (20.8 mg, 0.1 mmol) and benzoyl fluoride (49.6 mg, 0.4 mmol). After purification by flash chromatography (*n*-pentane/ethyl acetate = 8/1), the desired compound was obtained as a colorless oil (25.6 mg, 82% yield). ^{1}H NMR (300 MHz, $CDCl_3$) δ (ppm) 8.00 – 7.88 (m, 2H), 7.52 – 7.43 (m, 1H), 7.38 (ddt, J = 8.3, 6.6, 1.3 Hz, 2H), 7.24 – 7.15 (m, 2H), 6.87 – 6.76 (m, 2H), 4.65 (t, J = 7.3 Hz, 1H), 4.16 – 3.94 (m, 2H), 3.74 (s, 3H), 2.58 – 2.38 (m, 1H), 2.18 – 2.07 (m, 1H), 2.01 (s, 3H). ^{13}C NMR (75 MHz, $CDCl_3$) δ (ppm) 199.2 (C), 171.1 (C), 158.9 (C), 136.6 (C), 133.0 (CH), 130.6 (C), 129.4 (CH), 128.8 (CH), 128.6 (CH), 114.6 (CH), 62.6 (CH_2), 55.3 (CH_3), 49.4 (CH), 32.7 (CH_2), 21.0 (CH_3). HRMS (ESI) Calcd. for $C_{19}H_{20}NaO_4$ $[M+Na]^+$: 335.1259. Found: 335.1249. IR (neat, cm^{-1}): 1735, 1679, 1609, 1510, 1448, 1233, 1177, 1033, 819, 734, 690.

2-(4-Methoxyphenyl)-1-phenyl-5-(pyrrolidin-1-yl)pentane-1,5-dione (3s)

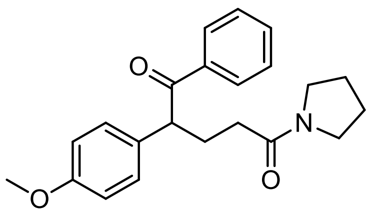

The reaction was performed according to general procedure with 4-(4-methoxyphenyl)-1-(pyrrolidin-1-yl)butan-1-one (24.7 mg, 0.1 mmol) and benzoyl fluoride (49.6 mg, 0.4 mmol). After purification by flash chromatography (*n*-pentane/ethyl acetate = 1/1), the desired compound was obtained as a yellow oil (29.6 mg, 84% yield). ^{1}H NMR (300 MHz, $CDCl_3$) δ (ppm) 8.00 – 7.90 (m, 2H), 7.47 – 7.38 (m, 1H), 7.38 – 7.29 (m, 2H), 7.24 – 7.16 (m, 2H), 6.86 – 6.73 (m, 2H), 4.78 (t, J = 7.2 Hz, 1H), 3.71 (s, 3H), 3.43 (td, J = 6.5, 2.1 Hz, 2H), 3.23 (t, J = 6.6 Hz, 2H), 2.51 – 2.35 (m, 1H), 2.27 – 2.05 (m, 3H), 1.88 – 1.69 (m, 4H). ^{13}C NMR (75

MHz, $CDCl_3$) δ (ppm) 200.2 (C), 171.2 (C), 158.7 (C), 136.8 (C), 132.8 (CH), 131.3 (C), 129.5 (CH), 128.8 (CH), 128.5 (CH), 114.3 (CH), 55.2 (CH_3), 51.4 (CH), 46.6 (CH_2), 45.7 (CH_2), 32.0 (CH_2), 28.9 (CH_2), 26.1 (CH_2), 24.4 (CH_2). HRMS (ESI) Calcd. for $C_{22}H_{25}NNaO_3$ $[M+Na]^+$: 374.1732. Found: 374.1723. IR (neat, cm^{-1}): 2975, 2871, 1679, 1634, 1610, 1511, 1440, 1248, 1177, 1115, 1035, 731, 690.

4-Azido-2-(4-methoxyphenyl)-1-phenylbutan-1-one (3t)

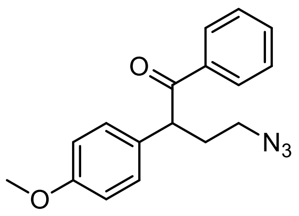

The reaction was performed according to general procedure with 1-(3-azidopropyl)-4-methoxybenzene (19.1 mg, 0.1 mmol) and benzoyl fluoride (49.6 mg, 0.4 mmol). After purification by flash chromatography (*n*-pentane/ethyl acetate = 40/1), the desired compound was obtained as a yellow oil (13.9 mg, 47% yield). ^1H NMR (300 MHz, $CDCl_3$) δ (ppm) 8.01 – 7.90 (m, 2H), 7.52 – 7.45 (m, 1H), 7.44 – 7.35 (m, 2H), 7.25 – 7.16 (m, 2H), 6.87 – 6.78 (m, 2H), 4.69 (t, *J* = 7.3 Hz, 1H), 3.75 (s, 3H), 3.34 (dt, *J* = 12.3, 6.1 Hz, 1H), 3.22 (ddd, *J* = 12.4, 7.9, 5.7 Hz, 1H), 2.47 – 2.30 (m, 1H), 2.15 – 1.99 (m, 1H). ^{13}C NMR (75 MHz, $CDCl_3$) δ (ppm) 199.2 (C), 159.0 (C), 136.5 (C), 133.1 (CH), 130.3 (C), 129.5 (CH), 128.9 (CH), 128.7 (CH), 114.7 (CH), 55.3 (CH_3), 49.5 (CH), 49.4 (CH_2), 32.8 (CH_2). HRMS (ESI) Calcd. for $C_{17}H_{17}N_3NaO_2$ $[M+Na]^+$: 318.1218. Found: 318.1212. IR (neat, cm^{-1}): 2956, 2934, 2838, 2098, 1679, 1609, 1597, 1581, 1511, 1448, 1350, 1303, 1250, 1178, 1112, 1033, 820, 734, 690.

2-(4-Methoxyphenyl)-1-phenylpentane-1,4-dione (3u)

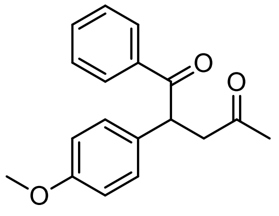

The reaction was performed according to general procedure with 4-(4-methoxyphenyl)butan-2-one (17.8 mg, 0.1 mmol) and benzoyl fluoride (49.6 mg, 0.4 mmol). After purification by flash chromatography (*n*-pentane/ethyl acetate = 20/1), the desired compound was obtained as a light yellow solid (18.5 mg, 66% yield). ^1H NMR (300 MHz, $CDCl_3$) δ (ppm) 8.02 – 7.88 (m, 2H), 7.46 (ddt, *J* = 8.3, 6.4, 1.4 Hz, 1H), 7.36 (ddt, *J* = 8.3, 6.9, 1.3 Hz, 2H), 7.22 – 7.11 (m, 2H), 6.87 – 6.70 (m, 2H), 5.06 (dd, *J* = 9.9, 4.1 Hz, 1H), 3.73 (s, 3H), 3.57 (dd, *J* = 17.9, 9.9 Hz, 1H), 2.74 (dd, *J* = 17.9, 4.1 Hz, 1H), 2.18 (s, 3H). ^{13}C NMR (75 MHz, $CDCl_3$) δ (ppm) 207.1, 199.2, 158.9, 136.4, 132.9, 130.5, 129.2, 129.0, 128.5, 114.7, 55.3, 48.2,

48.0, 30.2. HRMS (ESI) Calcd. for $C_{18}H_{18}NaO_3$ $[M+Na]^+$: 305.1154. Found: 305.1145. Spectroscopic data are in accordance with those described in literature.[32]

3-Methoxy-2-(4-methoxyphenyl)-1-phenylpropan-1-one (3v)

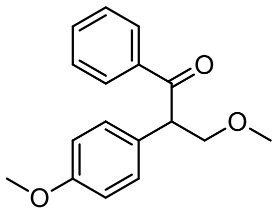

The reaction was performed according to general procedure with 1-methoxy-4-(2-methoxyethyl)benzene (16.6 mg, 0.1 mmol) and benzoyl fluoride (49.6 mg, 0.4 mmol). After purification by flash chromatography (*n*-pentane/ethyl acetate = 40/1), the desired compound was obtained as a yellow oil (8.5 mg, 31% yield). ^{1}H NMR (300 MHz, $CDCl_3$) δ (ppm) 7.91 – 7.83 (m, 2H), 7.43 – 7.35 (m, 1H), 7.33 – 7.24 (m, 2H), 7.19 – 7.10 (m, 2H), 6.80 – 6.69 (m, 2H), 4.74 (dd, J = 8.7, 5.4 Hz, 1H), 4.05 (dd, $J_1 = J_2$ = 8.9 Hz, 1H), 3.65 (s, 3H), 3.51 (dd, J = 9.1, 5.4 Hz, 1H), 3.25 (s, 3H). ^{13}C NMR (75 MHz, $CDCl_3$) δ (ppm) 198.6 (C), 159.0 (C), 136.8 (C), 133.1 (CH), 129.6 (C), 128.9 (CH), 128.6 (CH), 128.5 (CH), 114.6 (CH), 74.9 (CH_2), 59.3 (CH_3), 55.3 (CH_3), 53.0 (CH). HRMS (ESI) Calcd. for $C_{17}H_{18}NaO_3$ $[M+Na]^+$: 293.1154. Found: 293.1146. IR (neat, cm^{-1}): 2926, 2838, 1681, 1609, 1597, 1583, 1511, 1448, 1303, 1249, 1178, 1110, 1034, 964, 832, 803, 749, 691.

Methyl 2-(4-methoxyphenyl)-3-oxo-3-phenylpropanoate (3w)

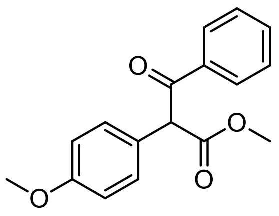

The reaction was performed according to general procedure with methyl 2-(4-methoxyphenyl)acetate (18.0 mg, 0.1 mmol) and benzoyl fluoride (49.6 mg, 0.4 mmol). After purification by flash chromatography (*n*-pentane/ethyl acetate = 10/1), the desired compound was obtained as a light yellow solid (17.1 mg, 60% yield). ^{1}H NMR (300 MHz, $CDCl_3$) δ (ppm) 7.95 (dt, J = 7.1, 1.4 Hz, 2H), 7.59 – 7.48 (m, 1H), 7.42 (td, J = 8.4, 7.2, 2.5 Hz, 2H), 7.33 (dd, J = 9.2, 2.6 Hz, 2H), 6.94 – 6.86 (m, 2H), 5.59 (s, 1H), 3.77 (s, 3H), 3.76 (s, 3H). ^{13}C NMR (75 MHz, $CDCl_3$) δ (ppm) 193.6, 169.7, 159.6, 135.7, 133.6, 130.8, 129.1, 128.8, 125.0, 114.5, 59.7, 55.4, 52.8. HRMS (ESI) Calcd. for $C_{17}H_{16}NaO_4$ $[M+Na]^+$: 307.0946. Found: 307.0937. Spectroscopic data are in accordance with those described in literature.[33]

(2,3-Dihydrobenzofuran-3-yl)(phenyl)methanone (3x)

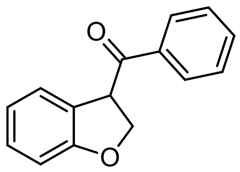

The reaction was performed according to general procedure with 2,3-dihydrobenzofuran (12.0 mg, 0.1 mmol) and benzoyl fluoride (49.6 mg, 0.4 mmol). $[Ir(dF(CF_3)ppy)_2(dtbbpy)]PF_6$ (2.2 mg, 0.002 mmol) was used instead of 4CzIPN. After purification by flash chromatography (*n*-pentane/ethyl acetate = 30/1), the desired compound was obtained as a white solid (18.3 mg, 82% yield), m.p. = 94-96 ℃. ^{1}H NMR (300 MHz, $CDCl_3$) δ (ppm) 8.11 – 8.00 (m, 2H), 7.71 – 7.61 (m, 1H), 7.61 – 7.49 (m, 2H), 7.19 – 7.13 (m, 1H), 7.02 – 6.94 (m, 1H), 6.86 (dd, *J* = 8.1, 0.9 Hz, 1H), 6.77 (td, *J* = 7.5, 1.0 Hz, 1H), 5.29 (dd, *J* = 9.5, 6.5 Hz, 1H), 5.13 (dd, *J* = 8.9, 6.5 Hz, 1H), 4.77 (dd, *J* = 9.5, 8.9 Hz, 1H). ^{13}C NMR (75 MHz, $CDCl_3$) δ (ppm) 196.4 (C), 160.2 (C), 136.2 (C), 133.9 (CH), 129.4 (CH), 129.2 (CH), 129.1 (CH), 125.3 (C), 125.1 (CH), 120.5 (CH), 110.2 (CH), 72.6 (CH_2), 49.5 (CH). HRMS (ESI) Calcd. for $C_{15}H_{12}NaO_2$ $[M+Na]^+$: 247.0735. Found: 247.0725.

Chroman-4-yl(phenyl)methanone (3y)

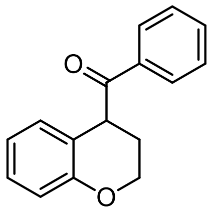

The reaction was performed according to general procedure with chroman (13.4 mg, 0.1 mmol) and benzoyl fluoride (49.6 mg, 0.4 mmol). $[Ir(dF(CF_3)ppy)_2(dtbbpy)]PF_6$ (2.2 mg, 0.002 mmol) was used instead of 4CzIPN. After purification by flash chromatography (*n*-pentane/ethyl acetate = 10/1), the desired compound was obtained as a grey solid (20.7 mg, 87% yield), m.p. = 93-95 ℃. ^{1}H NMR (300 MHz, $CDCl_3$) δ (ppm) 8.10 – 7.96 (m, 2H), 7.69 – 7.57 (m, 1H), 7.52 (dd, *J* = 8.2, 6.8 Hz, 2H), 7.16 (ddd, *J* = 8.5, 7.0, 1.8 Hz, 1H), 6.95 – 6.87 (m, 2H), 6.82 (td, *J* = 7.4, 1.3 Hz, 1H), 4.84 (t, *J* = 6.0 Hz, 1H), 4.37 – 4.13 (m, 2H), 2.43 – 2.19 (m, 2H). ^{13}C NMR (75 MHz, $CDCl_3$) δ (ppm) 201.1 (C), 155.3 (C), 136.3 (C), 133.5 (CH), 130.0 (CH), 129.0 (CH), 128.9 (CH), 128.6 (CH), 120.6 (CH), 120.0 (C), 117.5 (CH), 63.5 (CH_2), 42.4 (CH), 26.2 (CH_2). HRMS (ESI) Calcd. for $C_{16}H_{14}NaO_2$ $[M+Na]^+$: 261.0891. Found: 261.0885. IR (neat, cm^{-1}): 2934, 1684, 1581, 1489, 1448, 1228, 1211, 1118, 987, 755, 701.

2-(4-Phenoxyphenyl)-1-phenylpropan-1-one (3z)

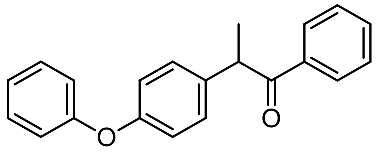

The reaction was performed according to general procedure with 1-ethyl-4-phenoxybenzene (19.8 mg, 0.1 mmol) and benzoyl fluoride (49.6 mg, 0.4 mmol). After purification by flash chromatography (*n*-pentane/ethyl acetate = 30/1), the desired compound was obtained as a white solid (14.2 mg, 47% yield). ^{1}H NMR (300 MHz, $CDCl_3$) δ (ppm) 8.01 – 7.93 (m, 2H), 7.55 – 7.46 (m, 1H), 7.46 – 7.37 (m, 2H), 7.37 – 7.28 (m, 2H), 7.28 – 7.22 (m, 2H), 7.10 (ddt, *J* = 7.8, 6.9, 1.1 Hz, 1H), 7.01 – 6.89 (m, 4H), 4.70 (q, *J* = 6.9 Hz, 1H), 1.54 (d, *J* = 6.9 Hz, 3H). ^{13}C NMR (75 MHz, $CDCl_3$) δ (ppm) 200.5, 157.0, 156.4, 136.1, 133.0, 129.9, 129.2, 128.9, 128.7, 123.5, 119.2, 119.1, 47.1, 19.7. HRMS (ESI) Calcd. for $C_{21}H_{18}NaO_2$ $[M+Na]^+$: 325.1204. Found: 325.1197. Spectroscopic data are in accordance with those described in literature.[34]

2-(4-Methoxyphenyl)-1-phenylethanone (3aa)

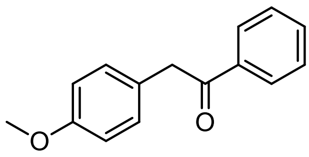

The reaction was performed according to general procedure with 1-methoxy-4-methylbenzene (12.2 mg, 0.1 mmol) and benzoyl fluoride (49.6 mg, 0.4 mmol). After purification by flash chromatography (*n*-pentane/ethyl acetate = 30/1), the desired compound was obtained as a light yellow solid (11.5 mg, 48% yield). 48% of starting 1-methoxy-4-methylbenzene was recovered. ^{1}H NMR (300 MHz, $CDCl_3$) δ (ppm) 8.06 – 7.94 (m, 2H), 7.61 – 7.51 (m, 1H), 7.49 – 7.39 (m, 2H), 7.22 – 7.13 (m, 2H), 6.91 – 6.80 (m, 2H), 4.23 (s, 2H), 3.78 (s, 3H). ^{13}C NMR (75 MHz, $CDCl_3$) δ (ppm) 198.1, 158.7, 136.7, 133.2, 130.6, 128.8, 128.7, 126.6, 114.3, 55.4, 44.8. HRMS (ESI) Calcd. for $C_{15}H_{14}NaO_2$ $[M+Na]^+$: 249.0891. Found: 249.0884. Spectroscopic data are in accordance with those described in literature.[35]

2-(4-Methoxyphenyl)-2-methyl-1-phenylpropan-1-one (3ab)

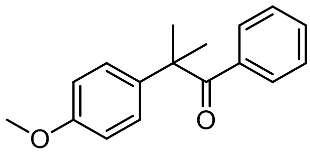

The reaction was performed according to general procedure with 1-isopropyl-4-methoxybenzene (15.0 mg, 0.1 mmol) and benzoyl fluoride (49.6 mg, 0.4 mmol). $[Ir(dF(CF_3)ppy)_2(dtbbpy)]PF_6$ (2.2 mg, 0.002 mmol) was used instead of 4CzIPN. After purification by flash chromatography (*n*-pentane/ethyl acetate = 30/1), the desired compound was obtained as a colorless oil (7.1 mg, 28% yield). ^{1}H NMR (400 MHz, $CDCl_3$) δ (ppm) 7.53 – 7.46 (m, 2H), 7.41 – 7.33 (m, 1H), 7.25 (td, *J*

= 8.5, 7.8, 6.2 Hz, 4H), 6.96 – 6.82 (m, 2H), 3.82 (s, 3H), 1.59 (s, 6H). ^{13}C NMR (100 MHz, $CDCl_3$) δ (ppm) 204.2, 158.5, 137.4, 136.6, 131.7, 129.8, 128.1, 127.0, 114.5, 55.4, 50.9, 28.0. HRMS (ESI) Calcd. for $C_{17}H_{18}NaO_2$ $[M+Na]^+$: 277.1204. Found: 277.1199. Spectroscopic data are in accordance with those described in literature.[36]

5-Bromo-1,2-diphenylpentan-1-one (3ac)

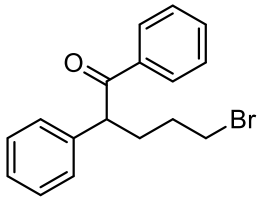

The reaction was performed according to general procedure with (4-bromobutyl)benzene (21.2 mg, 0.1 mmol) and benzoyl fluoride (49.6 mg, 0.4 mmol). After purification by flash chromatography (*n*-pentane/ethyl acetate = 30/1), the desired compound was obtained as a white solid (13.0 mg, 41% yield), m.p. = 99-101 ℃. ^{1}H NMR (300 MHz, $CDCl_3$) δ (ppm) 8.00 – 7.90 (m, 2H), 7.53 – 7.44 (m, 1H), 7.44 – 7.34 (m, 2H), 7.30 (d, J = 4.4 Hz, 4H), 7.24 – 7.18 (m, 1H), 4.56 (t, J = 7.2 Hz, 1H), 3.39 (td, J = 6.6, 0.8 Hz, 2H), 2.40 – 2.20 (m, 1H), 2.08 – 1.70 (m, 3H). ^{13}C NMR (75 MHz, $CDCl_3$) δ (ppm) 199.4 (C), 139.2 (C), 136.7 (C), 133.1 (CH), 129.2 (CH), 128.8 (CH), 128.7 (CH), 128.3 (CH), 127.4 (CH), 53.1 (CH), 33.5 (CH_2), 32.6 (CH_2), 30.9 (CH_2). HRMS (ESI) Calcd. for $C_{17}H_{17}BrNaO$ $[M+Na]^+$: 339.0360. Found: 339.0355. IR (neat, cm^{-1}): 3027, 1717, 1681, 1597, 1493, 1448, 1274, 1219, 1176, 1116, 1071, 1027, 754, 698.

2-(4-(Benzyloxy)phenyl)-1-phenylpropan-1-one (3ad)

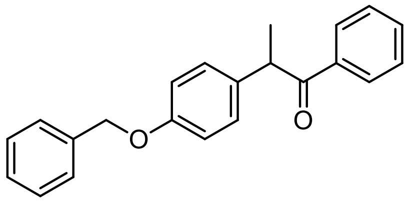

The reaction was performed according to general procedure with 1-(benzyloxy)-4-ethylbenzene (21.2 mg, 0.1 mmol) and benzoyl fluoride (49.6 mg, 0.4 mmol). After purification by flash chromatography (*n*-pentane/ethyl acetate = 30/1), the desired compound was obtained as a white solid (20.4 mg, 65% yield), m.p. = 93-95 ℃. ^{1}H NMR (300 MHz, $CDCl_3$) δ (ppm) 8.01 – 7.92 (m, 2H), 7.53 – 7.45 (m, 1H), 7.45 – 7.28 (m, 7H), 7.25 – 7.18 (m, 2H), 6.97 – 6.85 (m, 2H), 5.01 (s, 2H), 4.66 (q, J = 6.9 Hz, 1H), 1.53 (d, J = 6.8 Hz, 3H). ^{13}C NMR (75 MHz, $CDCl_3$) δ (ppm) 200.6 (C), 157.9 (C), 137.0 (C), 136.6 (C), 133.9 (C), 132.8 (CH), 128.9 (2xCH), 128.7 (CH), 128.6 (CH), 128.1 (CH), 127.6 (CH), 115.4 (CH), 70.11 (CH_2), 47.1 (CH), 19.64 (CH_3). HRMS (ESI) Calcd. for $C_{22}H_{20}NaO_2$ $[M+Na]^+$: 339.1361. Found: 339.1355. IR (neat, cm^{-1}): 2932, 1680, 1608, 1509, 1453, 1300, 1241, 1222, 1177, 1002, 952, 837, 738, 697.

(5-Methoxy-2,3-dihydro-1H-inden-1-yl)(phenyl)methanone (3ae)

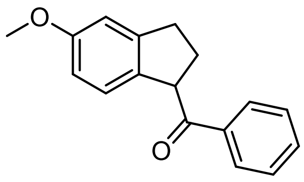

The reaction was performed according to general procedure with 5-methoxy-2,3-dihydro-1H-indene (14.8 mg, 0.1 mmol) and benzoyl fluoride (49.6 mg, 0.4 mmol). After purification by flash chromatography (*n*-pentane/ethyl acetate = 20/1), the desired compound was obtained as a light yellow oil (11.0 mg, 44% yield). ^{1}H NMR (300 MHz, $CDCl_3$) δ (ppm) 8.09 – 8.01 (m, 2H), 7.65 – 7.56 (m, 1H), 7.56 – 7.46 (m, 2H), 6.96 (dd, *J* = 8.4, 0.9 Hz, 1H), 6.88 – 6.80 (m, 1H), 6.66 (ddt, *J* = 8.4, 2.5, 0.8 Hz, 1H), 5.05 – 4.92 (m, 1H), 3.78 (s, 3H), 3.19 – 3.09 (m, 1H), 3.05 – 2.87 (m, 1H), 2.61 – 2.39 (m, 2H). ^{13}C NMR (75 MHz, $CDCl_3$) δ (ppm) 200.7 (C), 159.5 (C), 146.4 (C), 137.1 (C), 133.6 (C), 133.2 (CH), 129.0 (CH), 128.8 (CH), 125.7 (CH), 112.5 (CH), 110.2 (CH), 55.5 (CH_3), 51.8 (CH), 32.3 (CH_2), 30.1 (CH_2). HRMS (ESI) Calcd. for $C_{17}H_{16}NaO_2$ $[M+Na]^+$: 275.1048. Found: 275.1041. IR (neat, cm^{-1}): 2941, 1679, 1604, 1489, 1447, 1259, 1217, 1173, 1031, 807, 695.

(6-Methoxy-1,2,3,4-tetrahydronaphthalen-1-yl)(phenyl)methanone (3af)

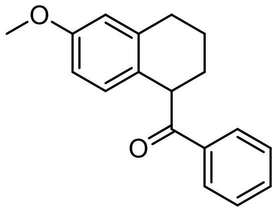

The reaction was performed according to general procedure with 6-methoxy-1,2,3,4-tetrahydronaphthalene (16.2 mg, 0.1 mmol) and benzoyl fluoride (49.6 mg, 0.4 mmol). After purification by flash chromatography (*n*-pentane/ethyl acetate = 20/1), the desired compound was obtained as a white solid (19.6 mg, 74% yield), m.p. = 86-88 ℃. ^{1}H NMR (300 MHz, $CDCl_3$) δ (ppm) 8.08 – 7.94 (m, 2H), 7.65 – 7.54 (m, 1H), 7.54 – 7.41 (m, 2H), 6.85 (d, *J* = 8.2 Hz, 1H), 6.77 – 6.60 (m, 2H), 4.78 (t, *J* = 6.6 Hz, 1H), 3.78 (s, 3H), 2.97 – 2.71 (m, 2H), 2.28 – 1.99 (m, 2H), 1.99 – 1.85 (m, 1H), 1.86 – 1.71 (m, 1H). ^{13}C NMR (75 MHz, $CDCl_3$) δ (ppm) 202.9, 158.2, 139.1, 136.7, 133.0, 130.4, 128.9, 128.8, 127.0, 114.0, 112.4, 55.3, 46.8, 29.8, 27.8, 20.7. HRMS (ESI) Calcd. for $C_{18}H_{18}NaO_2$ $[M+Na]^+$: 289.1204. Found: 289.1195. Spectroscopic data are in accordance with those described in literature.[37]

(8-Methoxy-1,2,3,4-tetrahydronaphthalen-1-yl)(phenyl)methanone (3ag)

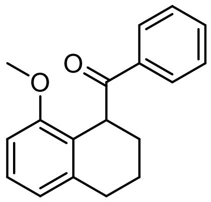

The reaction was performed according to general procedure with 5-methoxy-1,2,3,4-tetrahydronaphthalene (16.2 mg, 0.1 mmol) and benzoyl fluoride (49.6 mg, 0.4 mmol). After purification by flash chromatography (*n*-pentane/ethyl acetate = 20/1), the desired compound was obtained as a white solid (18.7 mg, 70% yield), m.p. = 87-89 ℃. ^{1}H NMR (300 MHz, $CDCl_3$) δ (ppm) 8.03 – 7.90 (m, 2H), 7.53 – 7.45 (m, 1H), 7.45 – 7.32 (m, 2H), 7.13 – 6.99 (m, 1H), 6.78 – 6.66 (m, 1H), 6.56 (dd, *J* = 8.2, 1.0 Hz, 1H), 4.88 – 4.70 (m, 1H), 3.49 (s, 3H), 2.87 – 2.59 (m, 2H), 2.10 – 1.95 (m, 1H), 1.95 – 1.56 (m, 3H). ^{13}C NMR (75 MHz, $CDCl_3$) δ (ppm) 203.0 (C), 156.8 (C), 139.5 (C), 137.1 (C), 132.5 (CH), 128.6 (CH), 128.4 (CH), 127.3 (CH), 124.6 (C), 121.8 (CH), 107.6 (CH), 55.3 (CH_3), 42.0 (CH), 29.5 (CH_2), 27.1 (CH_2), 20.1 (CH_2). HRMS (ESI) Calcd. for $C_{18}H_{18}NaO_2$ $[M+Na]^+$: 289.1204. Found: 289.1196. IR (neat, cm^{-1}): 2935, 1586, 1468, 1446, 1317, 1252, 1209, 1095, 1058, 989, 892, 766, 701, 609.

(5,6-Dimethoxy-2,3-dihydro-1H-inden-1-yl)(phenyl)methanone (3ah)

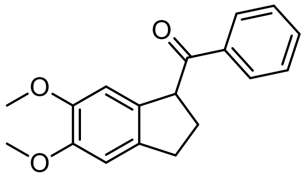

The reaction was performed according to general procedure with 5,6-dimethoxy-2,3-dihydro-1H-indene (17.8 mg, 0.1 mmol) and benzoyl fluoride (49.6 mg, 0.4 mmol). $[Ir(dF(CF_3)ppy)_2(dtbbpy)]PF_6$ (2.2 mg, 0.002 mmol) was used instead of 4CzIPN. After purification by flash chromatography (*n*-pentane/ethyl acetate = 10/1), the desired compound was obtained as a dark green solid (16.1 mg, 57% yield), m.p. = 86-88 ℃. ^{1}H NMR (300 MHz, $CDCl_3$) δ (ppm) 8.10 – 7.97 (m, 2H), 7.68 – 7.57 (m, 1H), 7.52 (ddt, *J* = 8.4, 6.6, 1.3 Hz, 2H), 6.81 (s, 1H), 6.59 (s, 1H), 4.98 (dd, *J* = 8.3, 6.7 Hz, 1H), 3.86 (s, 3H), 3.71 (s, 3H), 3.15 – 2.86 (m, 2H), 2.59 – 2.33 (m, 2H). ^{13}C NMR (75 MHz, $CDCl_3$) δ (ppm) 200.9 (C), 149.0 (C), 148.1 (C), 137.1 (C), 136.6 (C), 133.2 (CH), 133.0 (C), 128.9 (CH), 128.8 (CH), 108.4 (CH), 107.8 (CH), 56.1 ($2xCH_3$), 52.9 (CH), 32.1 (CH_2), 30.5 (CH_2). HRMS (ESI) Calcd. for $C_{18}H_{18}NaO_3$ $[M+Na]^+$: 305.1154. Found: 305.1145. IR (neat, cm^{-1}): 2936, 1682, 1596, 1505, 1448, 1307, 1268, 1212, 1085, 1024, 1001, 851, 696.

trans-(2-Methyl-2,3-dihydrobenzofuran-3-yl)(phenyl)methanone (3ai)

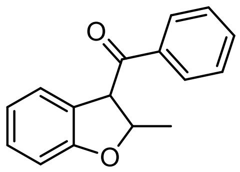

The reaction was performed according to general procedure with 2-methyl-2,3-dihydrobenzofuran (13.4 mg, 0.1 mmol) and benzoyl fluoride (49.6 mg, 0.4 mmol). [Ir(dF(CF_3)ppy)$_2$(dtbbpy)]PF_6 (2.2 mg, 0.002 mmol) was used instead of 4CzIPN. After purification by flash chromatography (*n*-pentane/ethyl acetate = 30/1), the desired compound was obtained as a white solid (16.4 mg, 69% yield, trans/cis = 98:2), m.p. = 97-99 ℃. ^{1}H NMR (300 MHz, $CDCl_3$) δ (ppm) 8.10 – 8.02 (m, 2H), 7.70 – 7.62 (m, 1H), 7.61 – 7.51 (m, 2H), 7.21 – 7.11 (m, 1H), 6.94 – 6.87 (m, 1H), 6.87 – 6.81 (m, 1H), 6.74 (td, *J* = 7.5, 1.0 Hz, 1H), 5.47 (p, *J* = 6.4 Hz, 1H), 4.87 (dt, *J* = 6.8, 1.0 Hz, 1H), 1.55 (d, *J* = 6.3 Hz, 3H). ^{13}C NMR (75 MHz, $CDCl_3$) δ (ppm) 196.6 (C), 159.5 (C), 136.6 (C), 133.9 (CH), 129.5 (CH), 129.2 (CH), 129.1 (CH), 125.5 (C), 125.0 (CH), 120.4 (CH), 110.3 (CH), 81.4 (CH), 57.0 (CH), 21.2 (CH_3). HRMS (ESI) Calcd. for $C_{16}H_{14}NaO_2$ $[M+Na]^+$: 261.0891. Found: 261.0886. IR (neat, cm^{-1}): 2976, 1683, 1595, 1478, 1448, 1293, 1235, 1163, 1013, 749, 695.

trans-(3-Methylchroman-4-yl)(phenyl)methanone (3aj)

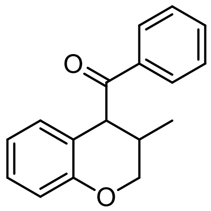

The reaction was performed according to general procedure with 3-methylchroman (14.8 mg, 0.1 mmol) and benzoyl fluoride (49.6 mg, 0.4 mmol). [Ir(dF(CF_3)ppy)$_2$(dtbbpy)]PF_6 (2.2 mg, 0.002 mmol) was used instead of 4CzIPN. After purification by flash chromatography (*n*-pentane/ethyl acetate = 20/1), the desired compound was obtained as a white solid (14.3 mg, 57% yield, trans/cis = 91/9), m.p. = 105-107 ℃. Data of the major trans-isomer are provided: ^{1}H NMR (300 MHz, $CDCl_3$) δ (ppm) 8.02 – 7.92 (m, 2H), 7.65 – 7.56 (m, 1H), 7.53 – 7.44 (m, 2H), 7.16 (ddd, *J* = 8.8, 6.8, 2.1 Hz, 1H), 6.94 – 6.74 (m, 3H), 4.43 (d, *J* = 6.7 Hz, 1H), 4.27 (dd, *J* = 10.9, 3.2 Hz, 1H), 3.86 (dd, *J* = 10.9, 7.4 Hz, 1H), 2.53 (ddt, *J* = 10.1, 6.9, 3.2 Hz, 1H), 1.10 (d, *J* = 6.8 Hz, 3H). ^{13}C NMR (75 MHz, $CDCl_3$) δ (ppm) 201.4 (C), 154.5 (C), 136.9 (C), 133.4 (CH), 129.7 (CH), 129.0 (CH), 129.0 (CH), 128.5 (CH), 120.9 (CH), 120.1 (C), 117.3 (CH), 69.2 (CH_2), 51.1 (CH), 30.8 (CH), 16.7 (CH_3). HRMS (ESI) Calcd. for $C_{17}H_{16}NaO_2$ $[M+Na]^+$: 275.1048. Found: 275.1041. IR (neat, cm^{-1}): 2965, 1680, 1581, 1489, 1447, 1228, 1209, 1048, 989, 754, 696.

Benzoylation of δ-tocopherol methyl ether (3ak)

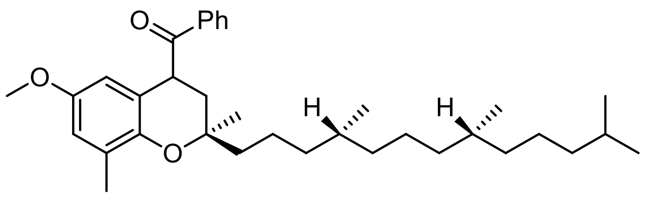

The reaction was performed according to general procedure with δ-tocopherol methyl ether (41.7 mg, 0.1 mmol) and benzoyl fluoride (49.6 mg, 0.4 mmol). [Ir(dF(CF_3)ppy)$_2$(dtbbpy)]PF_6 (2.2 mg, 0.002 mmol) was used instead of 4CzIPN. After purification by flash chromatography (*n*-pentane/ethyl acetate = 10/1), the desired compound was obtained as a colorless oil (17.7 mg, 34% yield, dr = 1/1). ^{1}H NMR (400 MHz, $CDCl_3$) δ (ppm) 7.94 (td, *J* = 7.8, 1.4 Hz, 2H), 7.64 – 7.53 (m, 1H), 7.47 (ddd, *J* = 9.7, 7.0, 2.2 Hz, 2H), 6.64 (d, *J* = 3.0 Hz, 1H), 6.26 (d, *J* = 3.0 Hz, 1H), 4.71 (ddd, *J* = 17.5, 11.0, 7.0 Hz, 1H), 3.61 (d, *J* = 2.6 Hz, 3H), 2.20 (d, *J* = 3.2 Hz, 3H), 2.16 – 1.94 (m, 2H), 1.63 (ddd, *J* = 22.6, 9.8, 5.7 Hz, 2H), 1.57 – 1.45 (m, 2H), 1.39 – 1.06 (m, 20H), 0.88 – 0.83 (m, 12H). ^{13}C NMR (100 MHz, $CDCl_3$) δ (ppm) 201.8 (2xC), 152.5 (2xC), 146.1 (C), 145.8 (C), 136.7 (C), 136.6 (C), 133.3 (2xCH), 129.1 (2xCH), 128.9 (CH), 128.2 (C), 128.1 (C), 119.9 (C), 119.8 (C), 116.3 (CH), 116.2 (CH), 110.6 (CH), 110.5 (CH), 75.8 (C), 75.7 (C), 55.7 (2xCH_3), 43.1 (2xCH), 42.9 (CH_2), 39.5 (CH_2), 37.6 (CH_2), 37.6 (CH_2), 37.5 (CH_2), 37.5 (CH_2), 37.4 (CH_2), 37.1 (CH_2), 36.7 (CH_2), 36.3 (CH_2), 33.0 (CH_3), 32.9 (CH_3), 32.8 (CH_3), 28.1 (CH), 26.5 (CH_3), 25.0 (CH_2), 24.6 (CH_2), 24.6 (CH_2), 22.9 (CH_3), 22.8 (CH_3), 22.7 (CH_3), 21.4 (CH_2), 21.0 (CH_2), 19.9 (CH_3), 19.8 (CH_3), 19.8 (CH_3), 16.6 (CH_3). HRMS (ESI) Calcd. for $C_{35}H_{52}NaO_3$ $[M+Na]^+$: 543.3814. Found: 543.3806. IR (neat, cm^{-1}): 2926, 2867, 1688, 1597, 1479, 1466, 1378, 1212, 1152, 1063, 919, 861, 781, 734, 707.

(3R,5S,8R,9S,10S,13S,14S)-10,13-Dimethyl-3-(4-(1-oxo-1-phenylpropan-2-yl)phenoxy)tetradecahydro-1H-cyclopenta[a]phenanthren-17(2H)-one (3al)

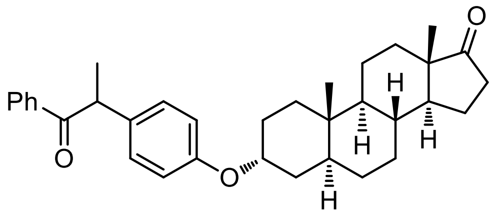

The reaction was performed according to general procedure with (3R,5S,8R,9S,10S,13S,14S)-3-(4-ethylphenoxy)-10,13-dimethyltetradecahydro-1H-cyclopenta[a] phenanthren-17(2H)-one (39.4 mg, 0.1 mmol) and benzoyl fluoride (49.6 mg, 0.4 mmol). After purification by flash chromatography (*n*-pentane/ethyl acetate = 10/1), the desired compound was obtained as a white solid (36.2 mg, 73% yield, dr = 1:1), m.p. = 132-134 ℃. ^{1}H NMR (300 MHz, $CDCl_3$) δ (ppm) 7.91 – 7.80 (m, 2H), 7.44 – 7.34 (m, 1H), 7.29 (dd, *J* = 8.1, 6.7 Hz, 2H), 7.13 – 7.03 (m, 2H), 6.77 – 6.64 (m, 2H), 4.55 (q, *J* = 6.8 Hz, 1H), 4.38 (t, *J* = 2.8 Hz, 1H), 2.35 (dd, *J* = 19.0, 8.7 Hz, 1H), 2.08 – 1.91 (m, 1H), 1.89 – 1.65 (m, 4H), 1.62 – 1.44 (m, 6H), 1.40 (d, *J* = 4.5

Hz, 3H), 1.36 – 1.03 (m, 8H), 0.94 (td, *J* = 11.5, 5.7 Hz, 1H), 0.76 (d, *J* = 7.6 Hz, 7H). ^{13}C NMR (75 MHz, $CDCl_3$) δ (ppm) 221.5 (C), 200.7 (C), 156.7 (C), 136.6 (C), 133.1 (C), 132.8 (CH), 128.8 (CH), 128.5 (CH), 116.4 (CH), 71.9 (CH), 54.3 (CH), 51.6 (CH), 47.9 (C), 47.0 (CH), 39.6 (CH), 36.0 (CH), 35.9 (CH_2), 35.1 (CH), 32.8 (CH_2), 32.7 (CH_2), 31.6 (CH_2), 30.8 (CH_2), 28.2 (CH_2), 25.7 (CH_2), 21.8 (CH_2), 20.2 (CH_2), 19.6 (CH_3), 13.9 (CH_3), 11.5 (CH_3). HRMS (ESI) Calcd. for $C_{34}H_{42}NaO_3$ $[M+Na]^+$: 521.3032. Found: 521.3022. IR (neat, cm^{-1}): 2927, 2856, 1737, 1682, 1507, 1448, 1242, 1167, 1118, 993, 952, 832, 730, 702.

tert-Butyl (2-(3,4-dimethoxyphenyl)-3-oxo-3-phenylpropyl)carbamate (3am)

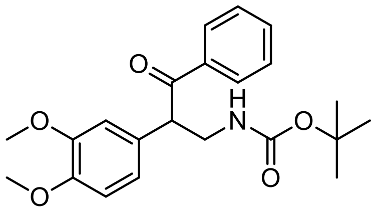

The reaction was performed according to general procedure with tert-butyl 3,4-dimethoxyphenethylcarbamate (28.1 mg, 0.1 mmol) and benzoyl fluoride (49.6 mg, 0.4 mmol). After purification by flash chromatography (*n*-pentane/ethyl acetate = 3/1), the desired compound was obtained as a colorless oil (20.0 mg, 52% yield). ^{1}H NMR (400 MHz, $CDCl_3$) δ (ppm) 7.93 – 7.80 (m, 2H), 7.47 – 7.36 (m, 1H), 7.30 (dd, *J* = 8.4, 7.0 Hz, 2H), 6.77 – 6.65 (m, 3H), 4.91 (t, *J* = 6.5 Hz, 1H), 4.76 (dd, *J* = 8.6, 5.6 Hz, 1H), 3.77 (s, 3H), 3.74 (s, 3H), 3.63 – 3.43 (m, 2H), 1.33 (s, 9H). ^{13}C NMR (100 MHz, $CDCl_3$) δ (ppm) 199.6 (C), 156.1 (C), 149.5 (C), 148.5 (C), 136.5 (C), 133.3 (CH), 129.4 (CH), 128.9 (CH), 128.7 (CH), 120.8 (CH), 111.7 (CH), 111.2 (CH), 79.4 (C), 56.0 (CH_3), 55.9 (CH_3), 53.6 (CH), 43.9 (CH_2), 28.5 (CH_3). HRMS (ESI) Calcd. for $C_{22}H_{27}NNaO_5$ $[M+Na]^+$: 408.1787. Found: 408.1785. IR (neat, cm^{-1}): 2925, 2855, 1706, 1678, 1594, 1514, 1448, 1262, 1241, 1165, 1027, 766, 691.

(4-d-Chroman-4-yl)(phenyl)methanone (3y-d$_1$)

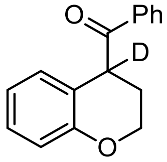

The reaction was performed according to general procedure with 4,4-d$_2$-chroman (13.6 mg, 0.1 mmol) and benzoyl fluoride (49.6 mg, 0.4 mmol). [Ir(dF(CF_3)ppy)$_2$(dtbbpy)]PF_6 (2.2 mg, 0.002 mmol) was used instead of 4CzIPN. After purification by flash chromatography (*n*-pentane/ethyl acetate = 10/1), the desired compound was obtained as a white solid (7.9 mg, 33% yield), m.p. = 93-95 °C. ^{1}H NMR (400 MHz, $CDCl_3$) δ (ppm) 8.09 – 7.98 (m, 2H), 7.66 – 7.56 (m, 1H), 7.52 (dd, *J* = 8.4, 7.0 Hz, 2H), 7.16 (ddd, *J* = 8.6, 7.2, 1.8 Hz, 1H), 6.90 (dt, *J* = 8.0, 1.8 Hz, 2H), 6.82 (td, *J* = 7.4, 1.3 Hz, 1H), 4.31 (ddd, *J* = 11.6, 8.6, 3.2 Hz, 1H), 4.22 (ddd, *J* = 11.1, 5.9, 3.6 Hz, 1H),

2.39 – 2.18 (m, 2H). ^{13}C NMR (100 MHz, $CDCl_3$) δ (ppm) 201.2 (C), 155.3 (C), 136.3 (C), 133.5 (CH), 130.0 (CH), 129.0 (CH), 128.9 (CH), 128.6 (CH), 120.7 (CH), 120.0 (C), 117.5 (CH), 63.5 (CH_2), 42.4 (CH), 26.1 (CH_2). HRMS (ESI) Calcd. for $C_{16}H_{13}DNaO_2$ $[M+Na]^+$: 262.0954. Found: 262.0946. IR (neat, cm^{-1}): 2927, 1677, 1596, 1581, 1489, 1448, 1280, 1258, 1220, 1181, 1118, 1012, 911, 755, 699.

Friedel Crafts acylation of 4-ethylanisole with 1a

(5-Ethyl-2-methoxyphenyl)(phenyl)methanone

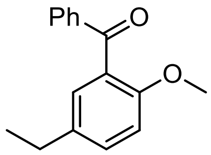

To a solution of 4-ethylanisole (680 mg, 5 mmol) in dry CH_2Cl_2 (8 mL) at 0 ℃ aluminum chloride (798 mg, 6 mmol) was added. Then the reaction mixture was treated dropwise with benzoyl fluoride (744 mg, 6 mmol) and the mixture was stirred at 0 ℃ for 1 h under N_2. After that, the mixture was stirred at room temperature for 12 h. Then the reaction was poured into ice water and extracted with CH_2Cl_2. The organic layer was dried (anhydrous Na_2SO_4), filtered and the solvent removed using rotary evaporation. Purification of the desired compound was performed by flash chromatography (*n*-pentane/ethyl acetate = 20/1). The desired product was obtained as a colorless oil (860 mg, 72% yield). ^1H NMR (300 MHz, $CDCl_3$) δ (ppm) 7.90 – 7.75 (m, 2H), 7.60 – 7.49 (m, 1H), 7.43 (ddt, J = 8.2, 6.7, 1.2 Hz, 2H), 7.29 (dd, J = 8.4, 2.3 Hz, 1H), 7.20 (d, J = 2.3 Hz, 1H), 6.91 (d, J = 8.5 Hz, 1H), 3.69 (s, 3H), 2.63 (q, J = 7.6 Hz, 2H), 1.23 (t, J = 7.6 Hz, 3H). ^{13}C NMR (75 MHz, $CDCl_3$) δ (ppm) 196.9 (C), 155.6 (C), 138.0 (C), 136.4 (C), 132.9 (CH), 131.2 (CH), 129.9 (CH), 129.0 (CH), 128.8 (C), 128.3 (CH), 111.6 (CH), 55.9 (CH_3), 28.0 (CH_2), 15.8 (CH_3). HRMS (ESI) Calcd. for $C_{16}H_{16}NaO_2$ $[M+Na]^+$: 263.1048. Found: 263.1039. IR (neat, cm^{-1}): 1661, 1581, 1495, 1449, 1293, 1263, 1240, 1177, 1117, 1025, 951, 840, 819, 705, 646.

^{1}H, ^{13}C and ^{19}F NMR spectra

2-(4-Methoxyphenyl)-1-phenylpropan-1-one (3a)

^{1}H NMR (300 MHz, $CDCl_3$, 300 K)

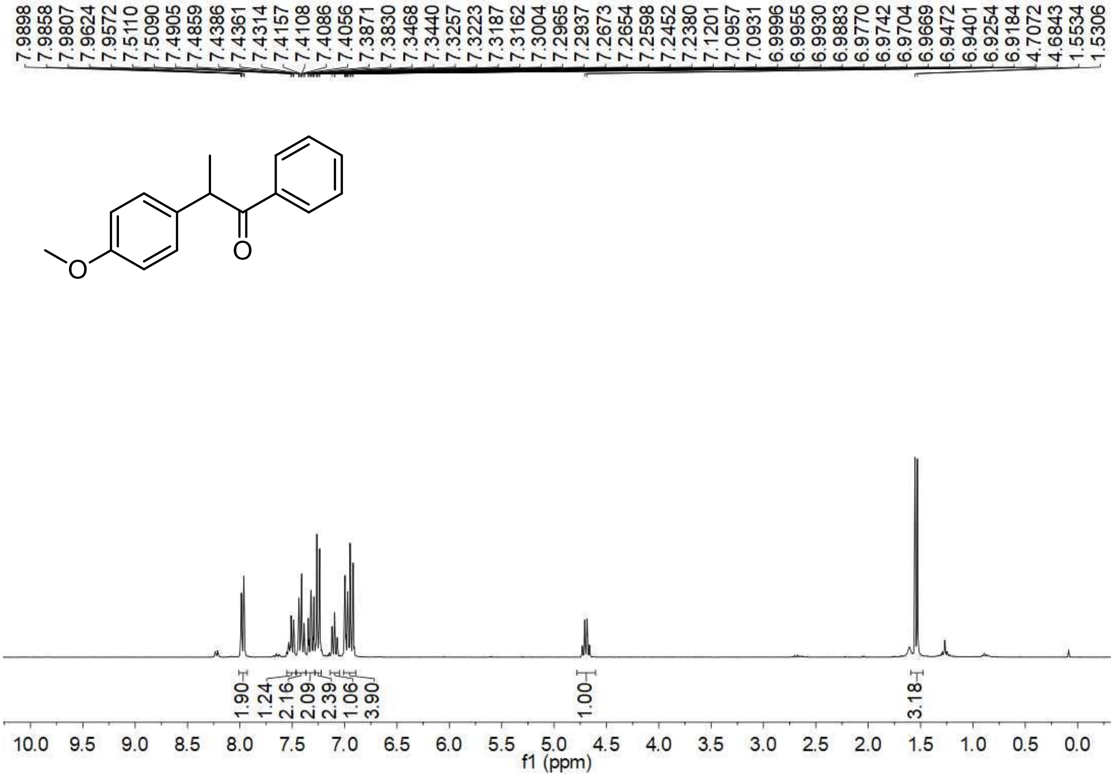

^{13}C NMR (75 MHz, $CDCl_3$, 300 K)

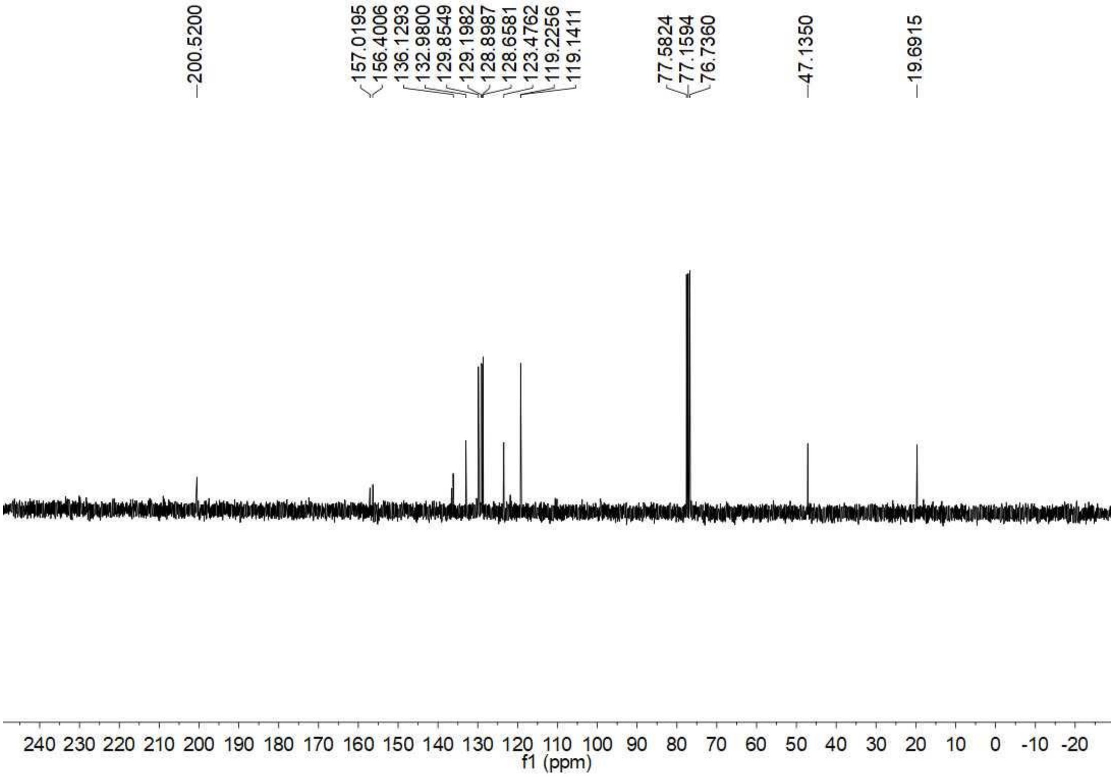

1,2-Bis(4-methoxyphenyl)propan-1-one (3b)

^{1}H NMR (300 MHz, $CDCl_3$, 300 K)

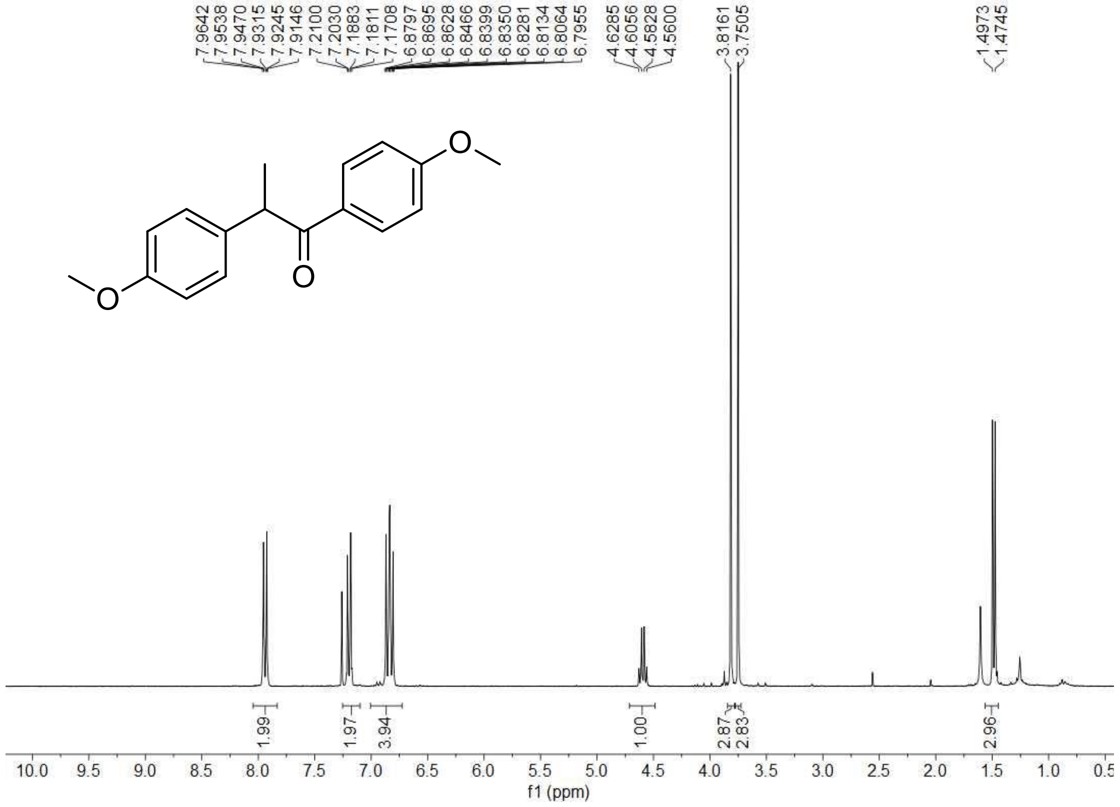

^{13}C NMR (75 MHz, $CDCl_3$, 300 K)

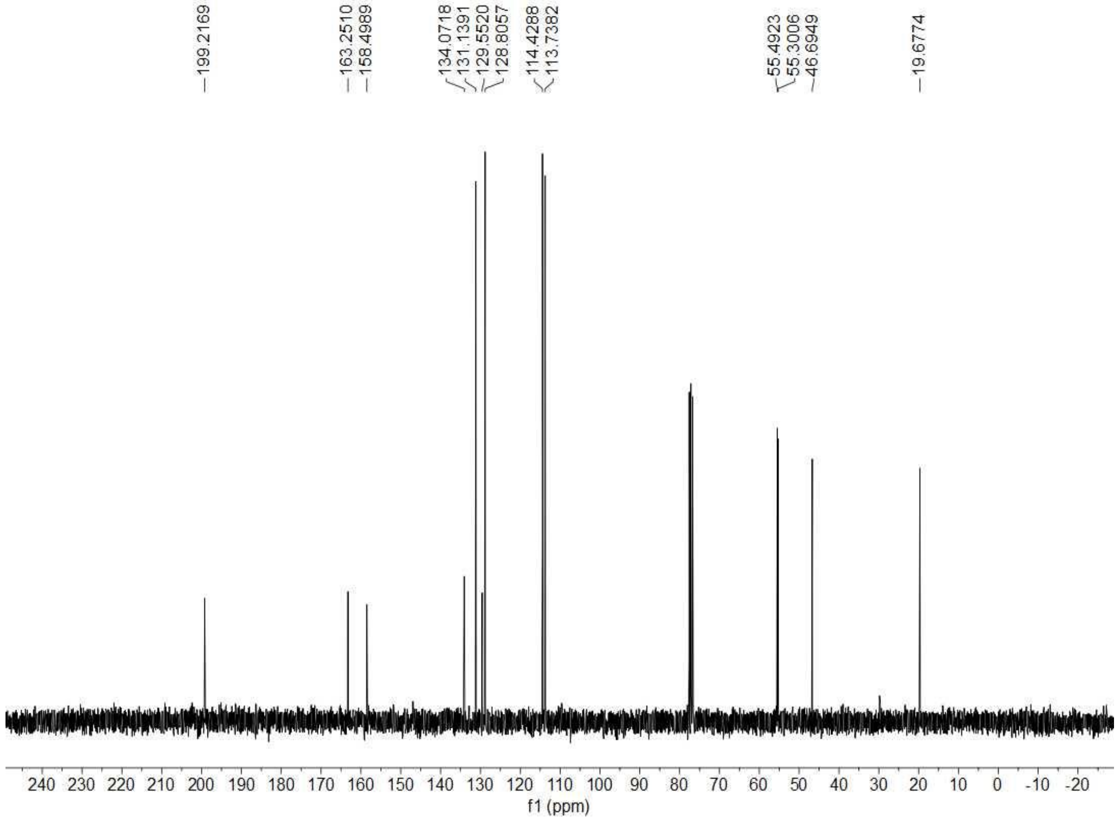

1-(4-Fluorophenyl)-2-(4-methoxyphenyl)propan-1-one (3c)

^{1}H NMR (300 MHz, $CDCl_3$, 300 K)

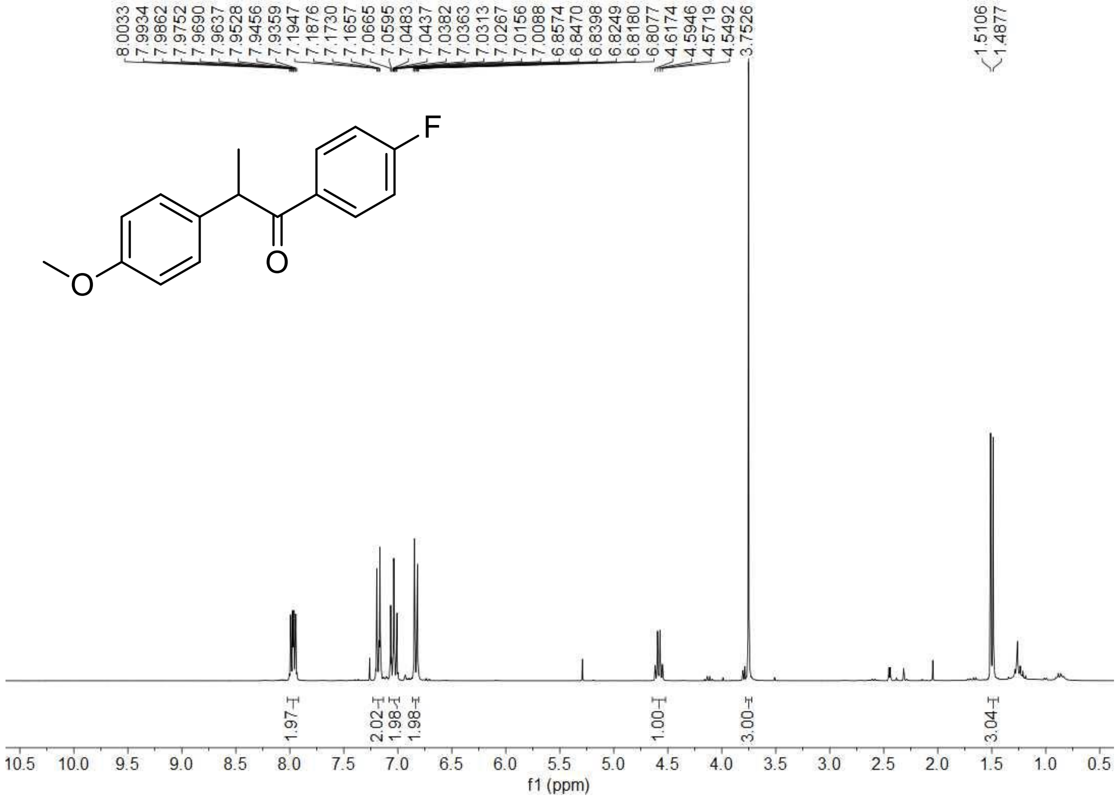

^{13}C NMR (75 MHz, $CDCl_3$, 300 K)

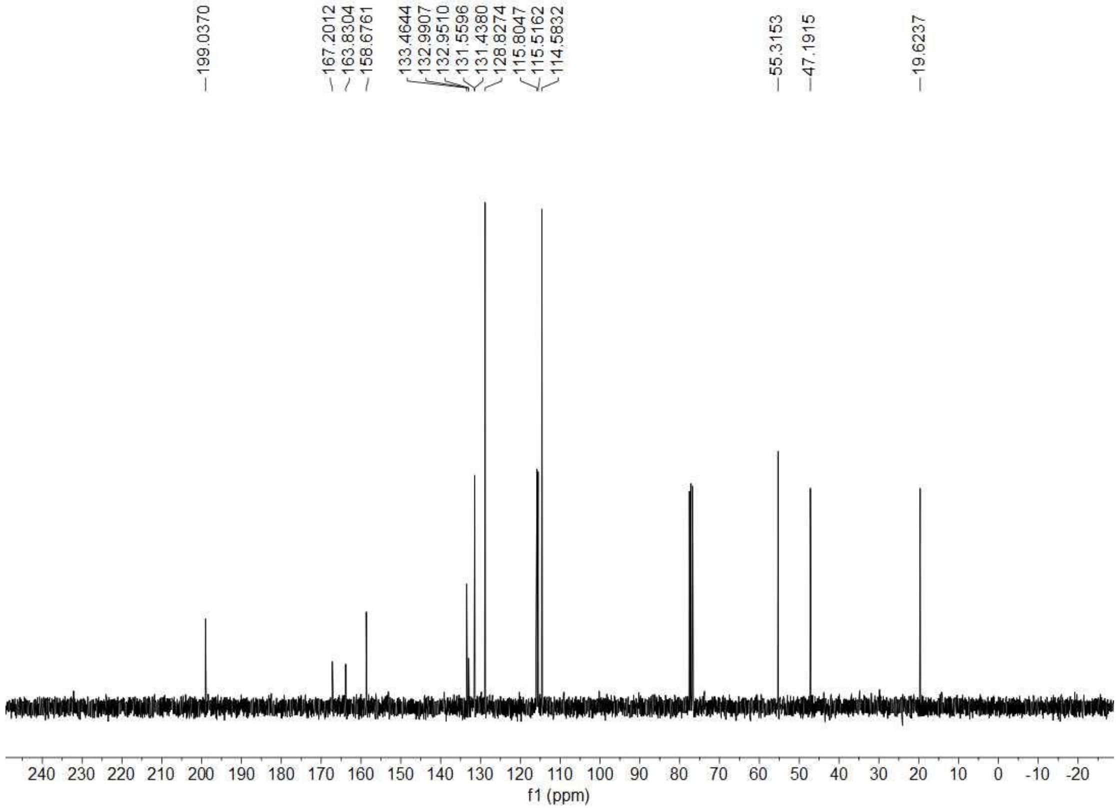

$^{19}F\{^1H\}$ NMR (282 MHz, $CDCl_3$, 300 K)

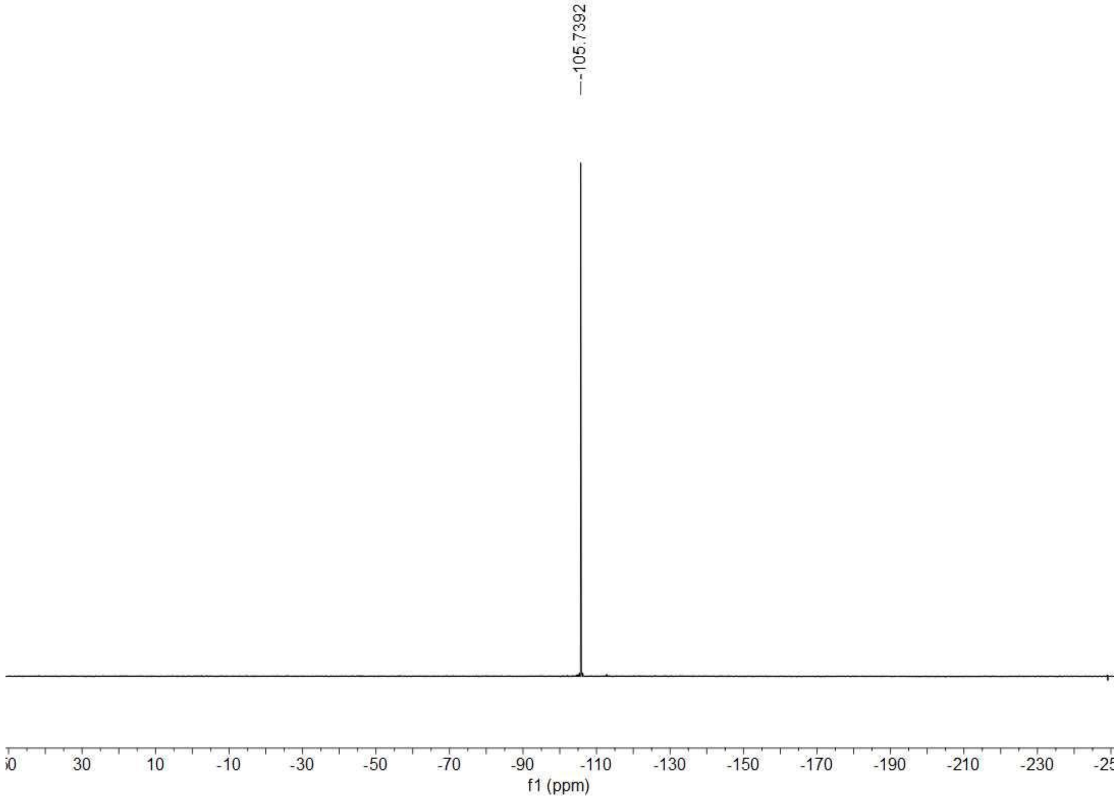

1-(4-Chlorophenyl)-2-(4-methoxyphenyl)propan-1-one (3d)

^1H NMR (300 MHz, $CDCl_3$, 300 K)

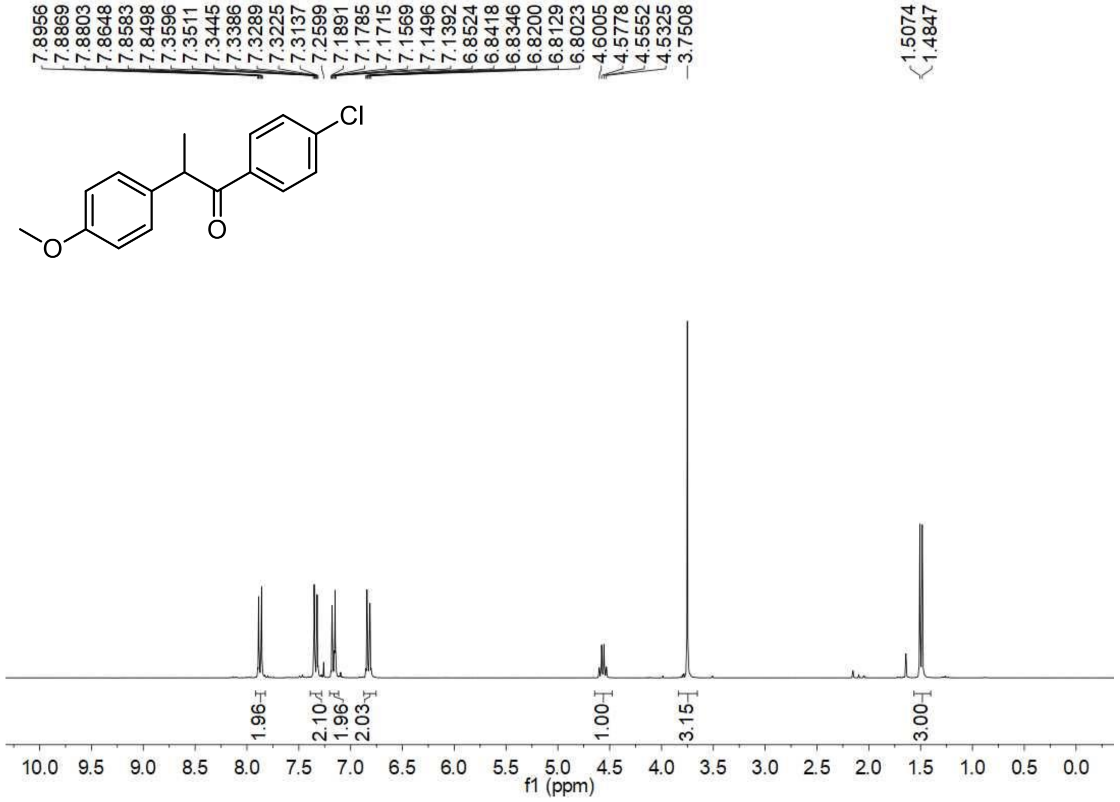

^{13}C NMR (75 MHz, $CDCl_3$, 300 K)

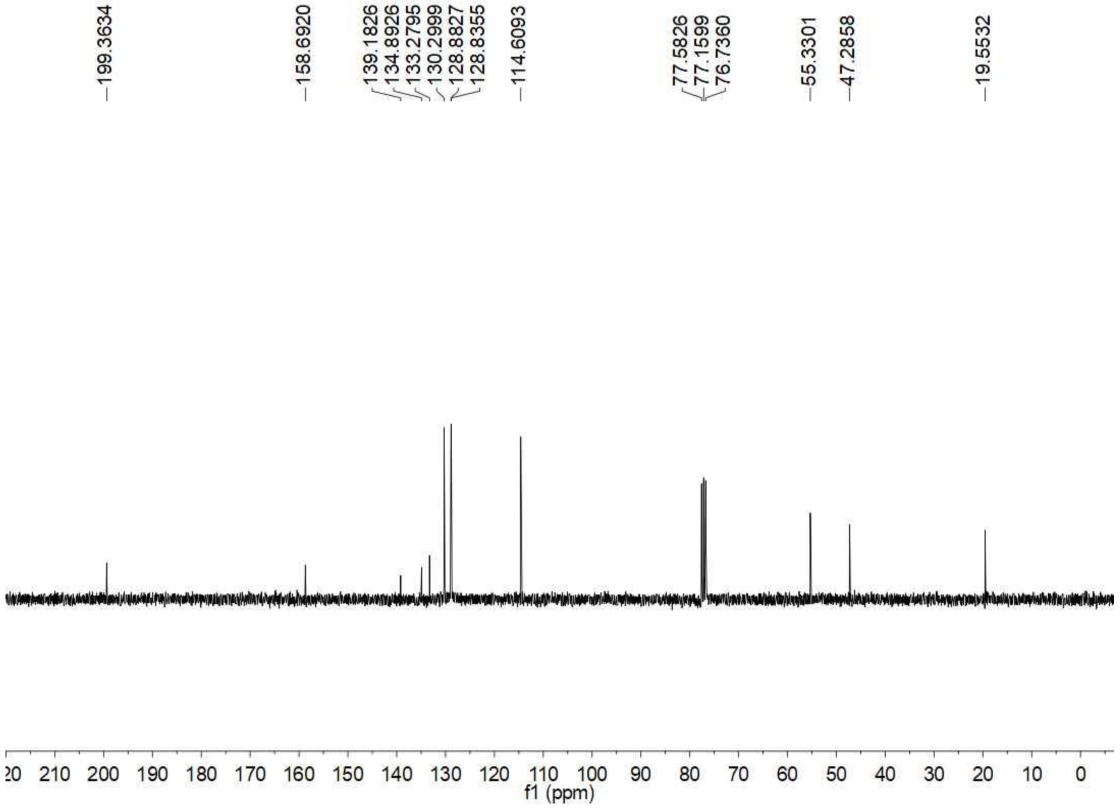

1-(4-Bromophenyl)-2-(4-methoxyphenyl)propan-1-one (3e)

^{1}H NMR (300 MHz, $CDCl_3$, 300 K)

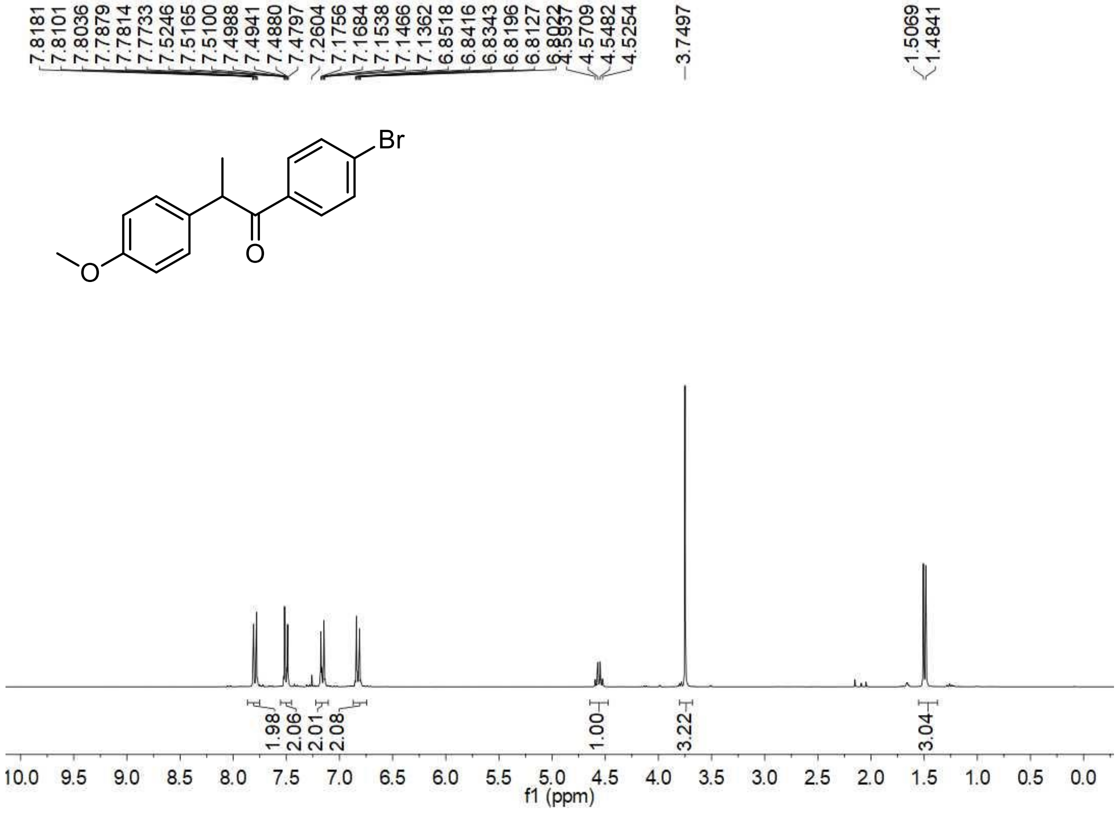

^{13}C NMR (75 MHz, $CDCl_3$, 300 K)

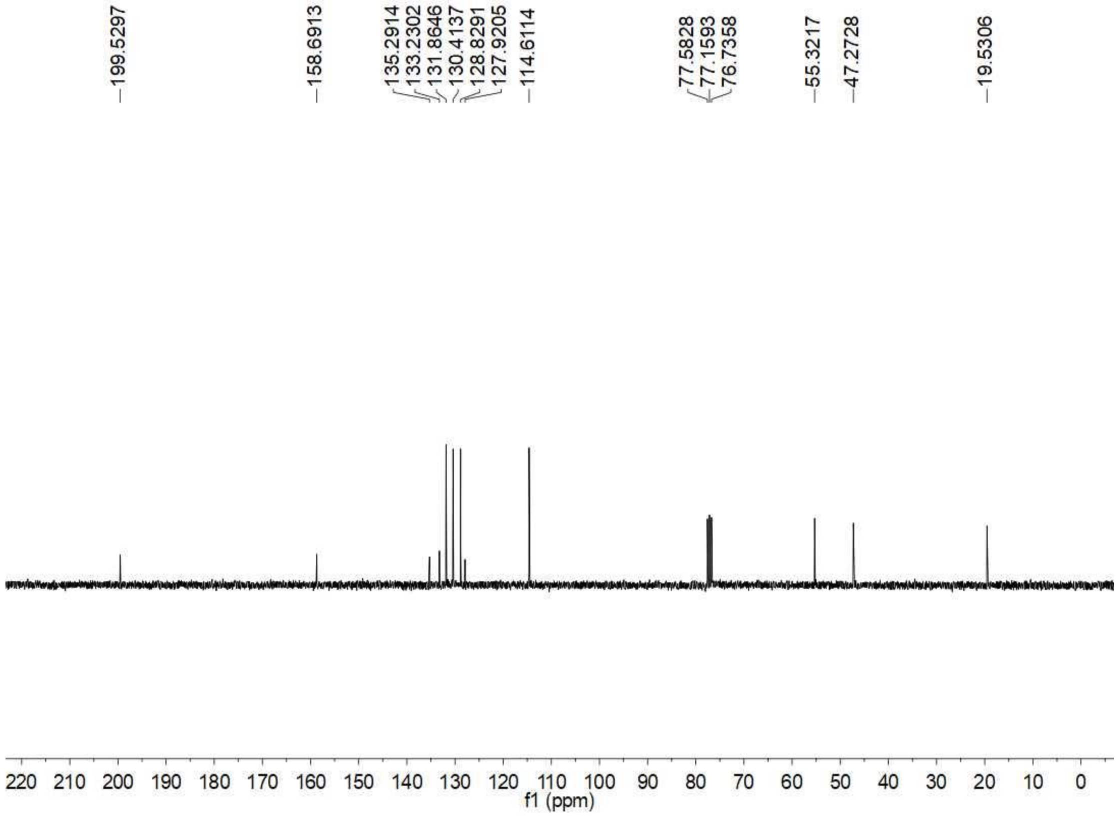

1-(4-Iodophenyl)-2-(4-methoxyphenyl)propan-1-one (3f)

^{1}H NMR (300 MHz, $CDCl_3$, 300 K)

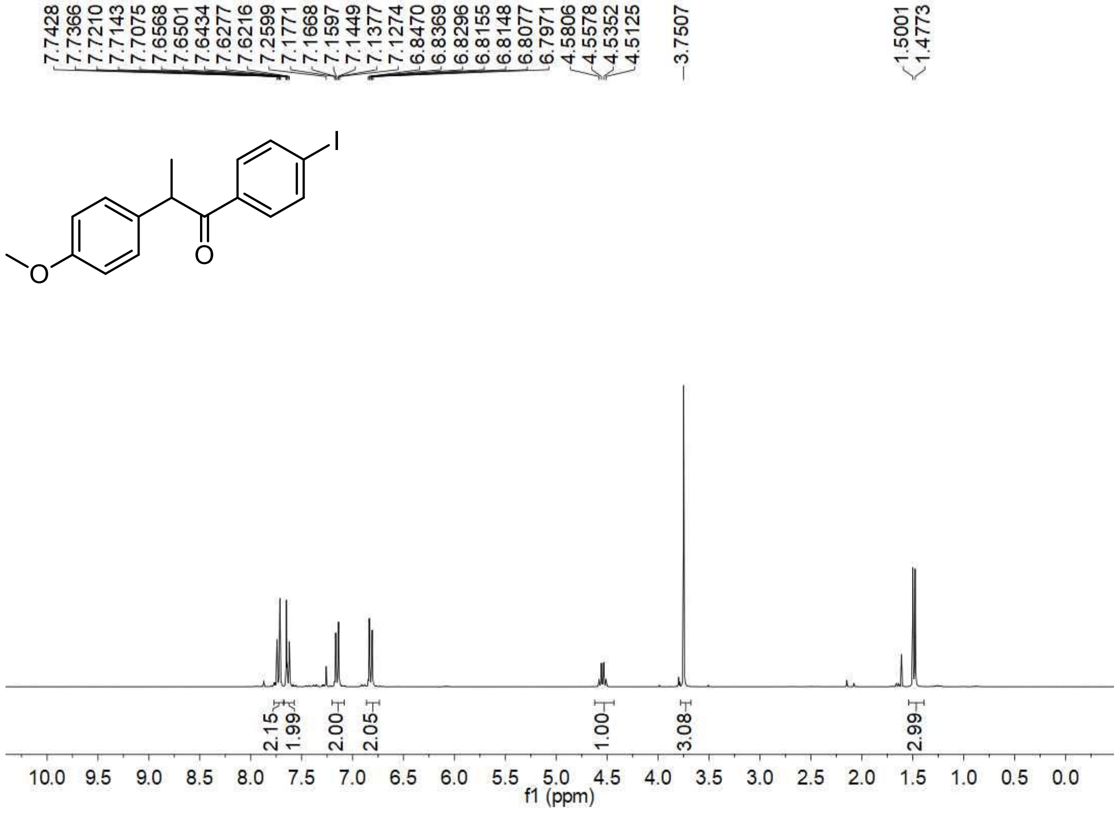

^{13}C NMR (75 MHz, $CDCl_3$, 300 K)

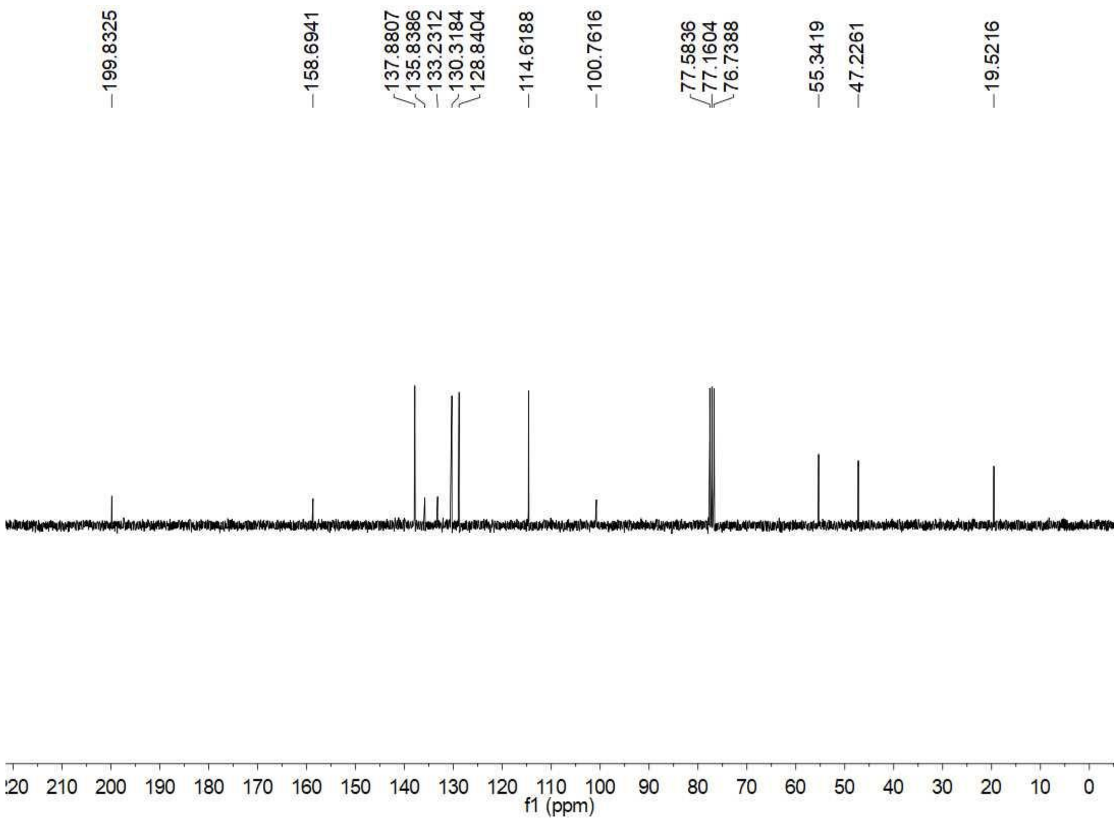

2-(4-Methoxyphenyl)-1-(4-(trifluoromethoxy)phenyl)propan-1-one (3g)

^{1}H NMR (300 MHz, $CDCl_3$, 300 K)

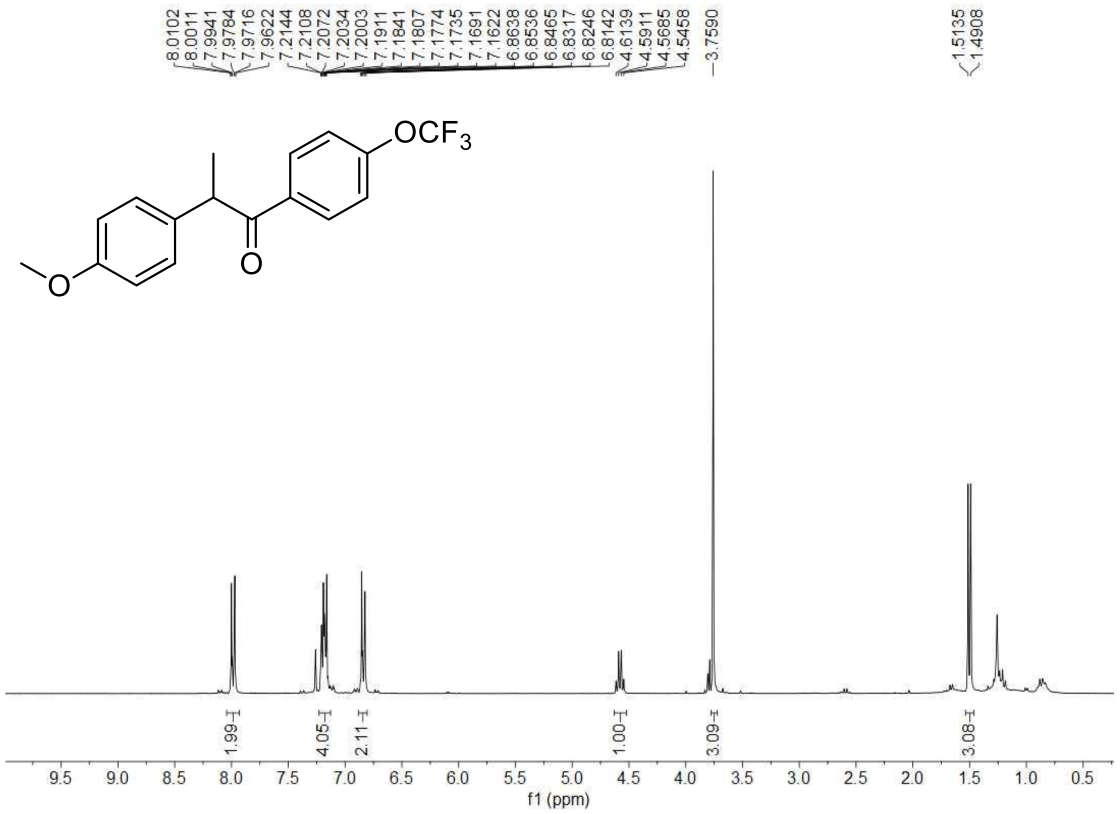

^{13}C NMR (100 MHz, $CDCl_3$, 300 K)

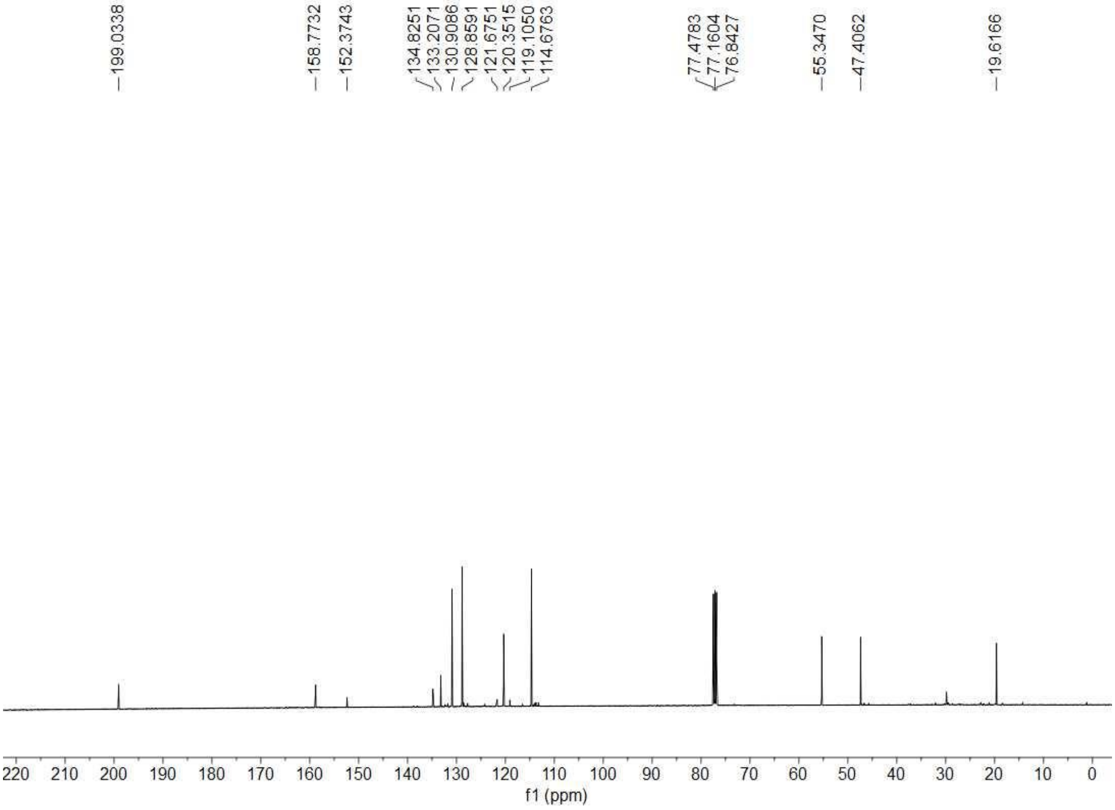

^{19}F {^{1}H} NMR (282 MHz, $CDCl_3$, 300 K)

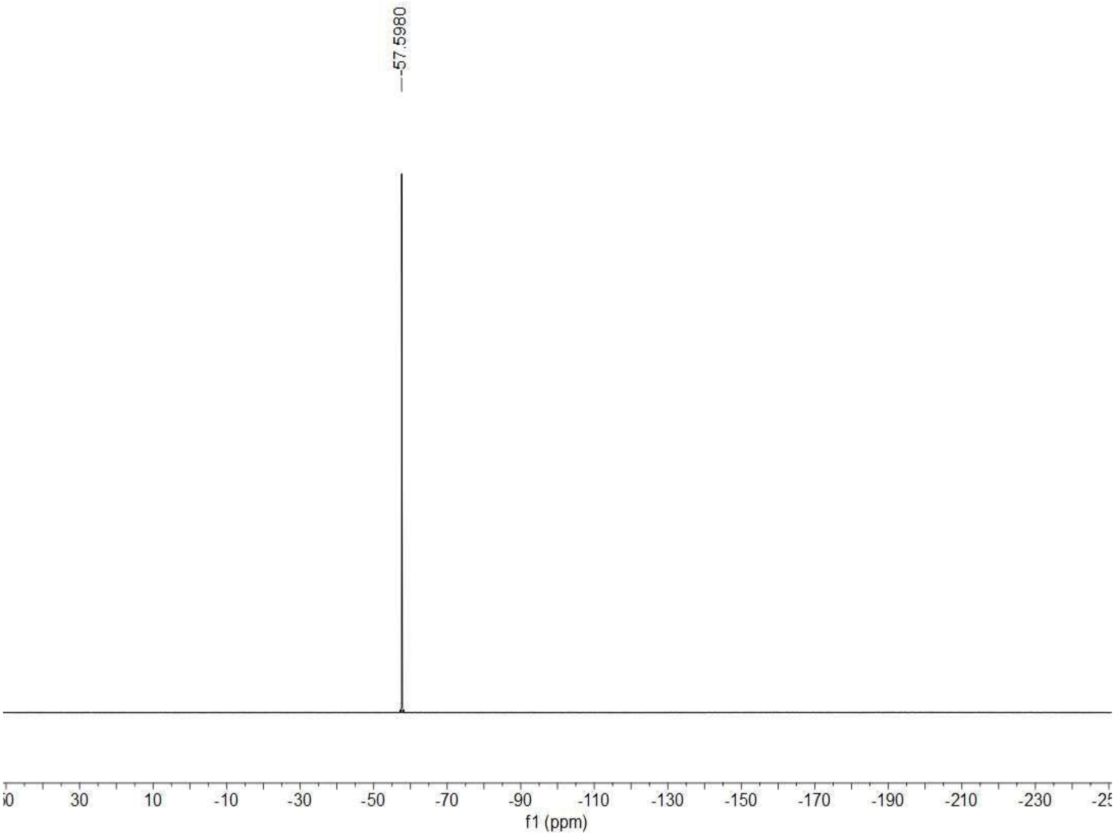

4-(2-(4-Methoxyphenyl)propanoyl)benzonitrile (3h)

^{1}H NMR (300 MHz, $CDCl_3$, 300 K)

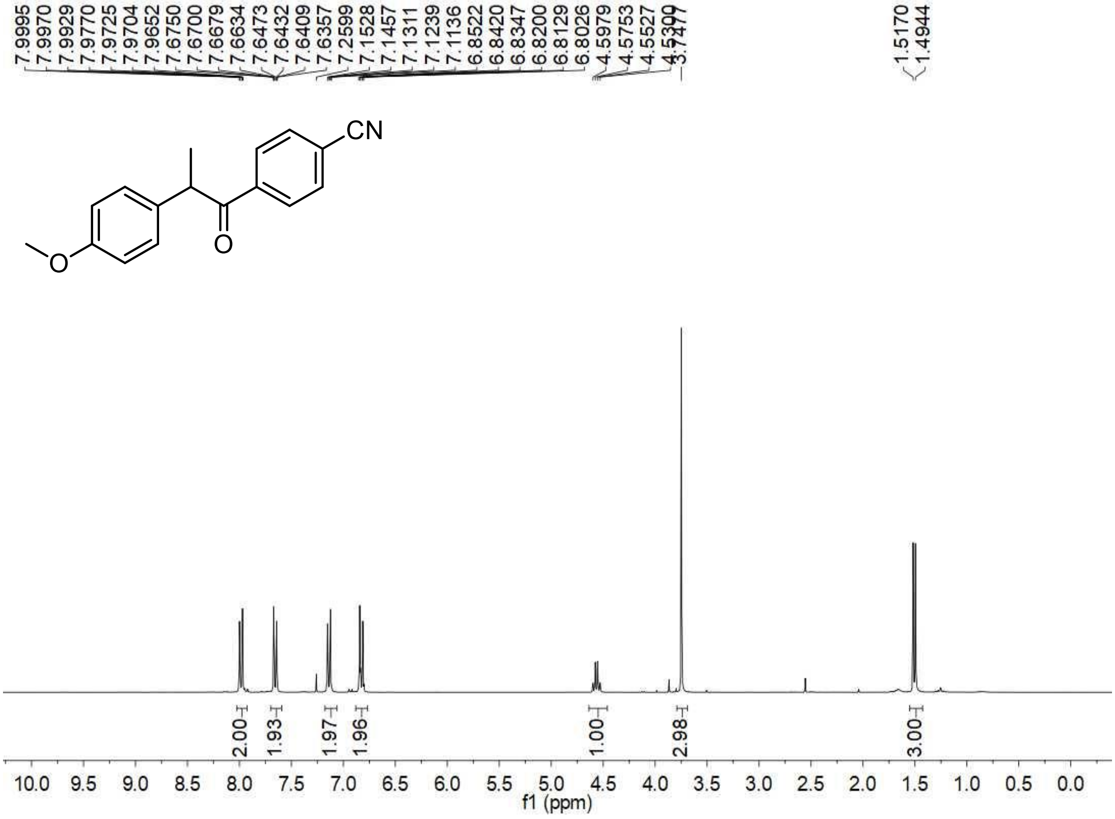

^{13}C NMR (75 MHz, $CDCl_3$, 300 K)

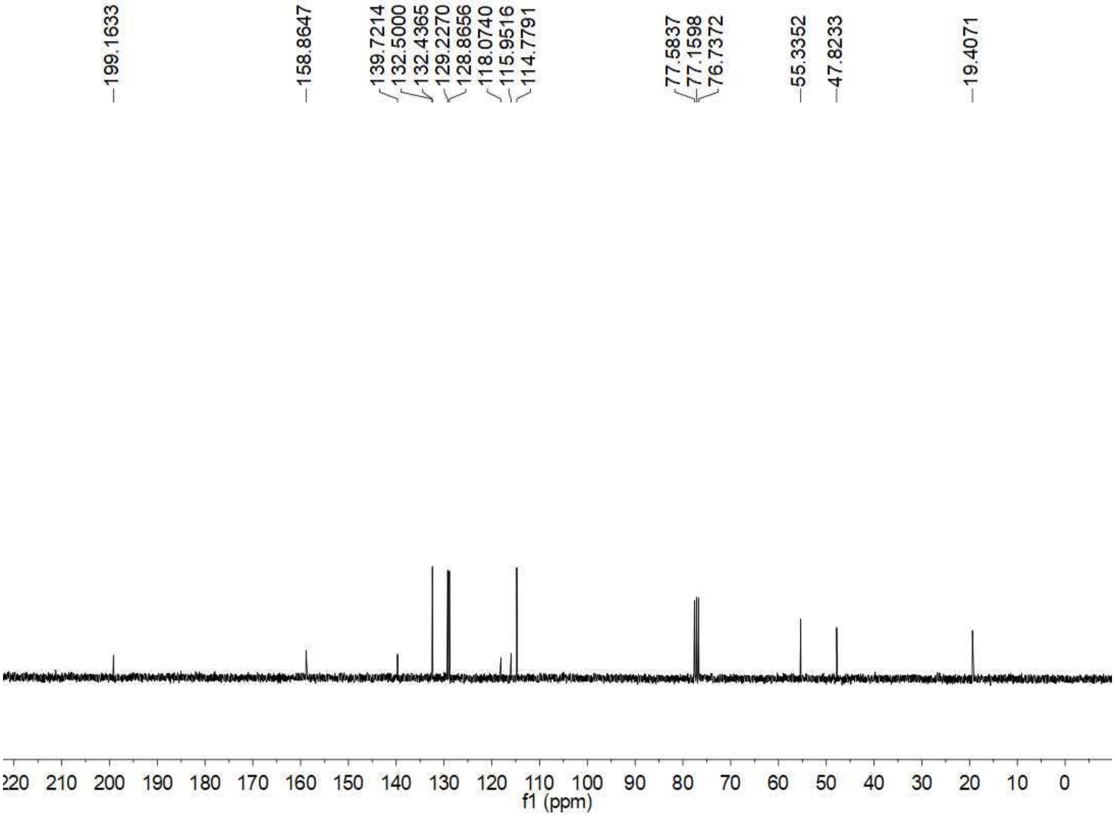

2-(4-Methoxyphenyl)-1-(m-tolyl)propan-1-one (3i)

^{1}H NMR (300 MHz, $CDCl_3$, 300 K)

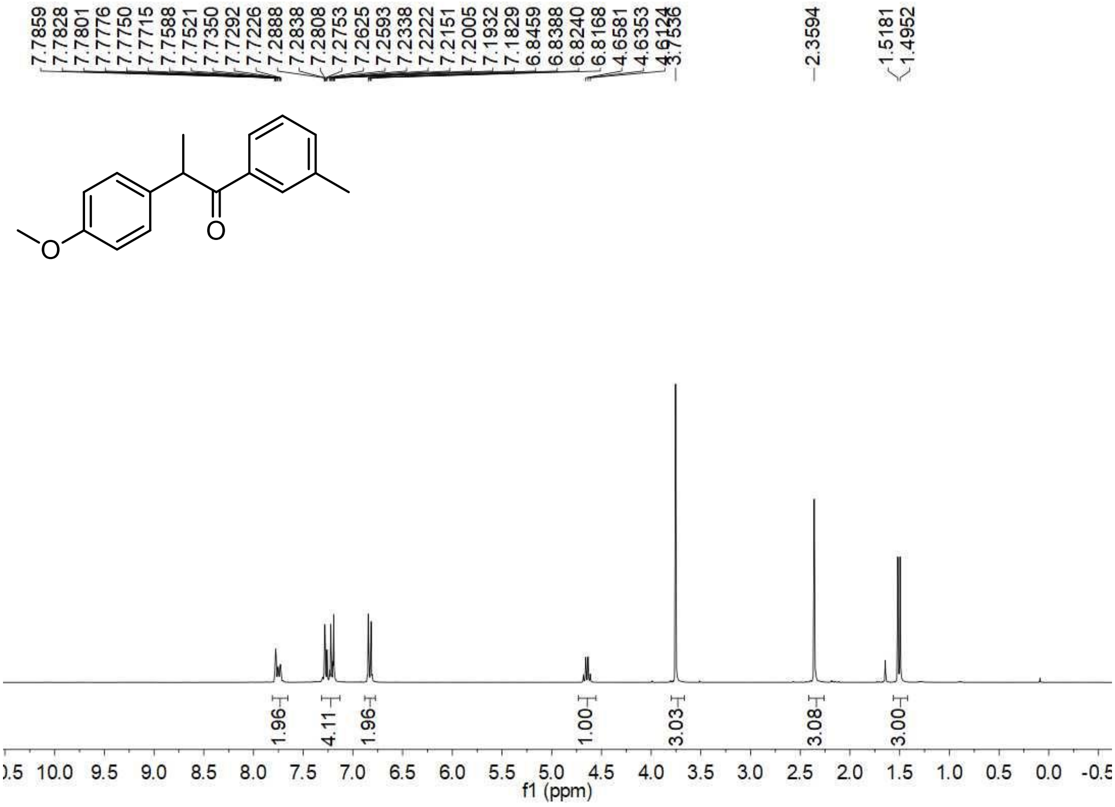

^{13}C NMR (75 MHz, $CDCl_3$, 300 K)

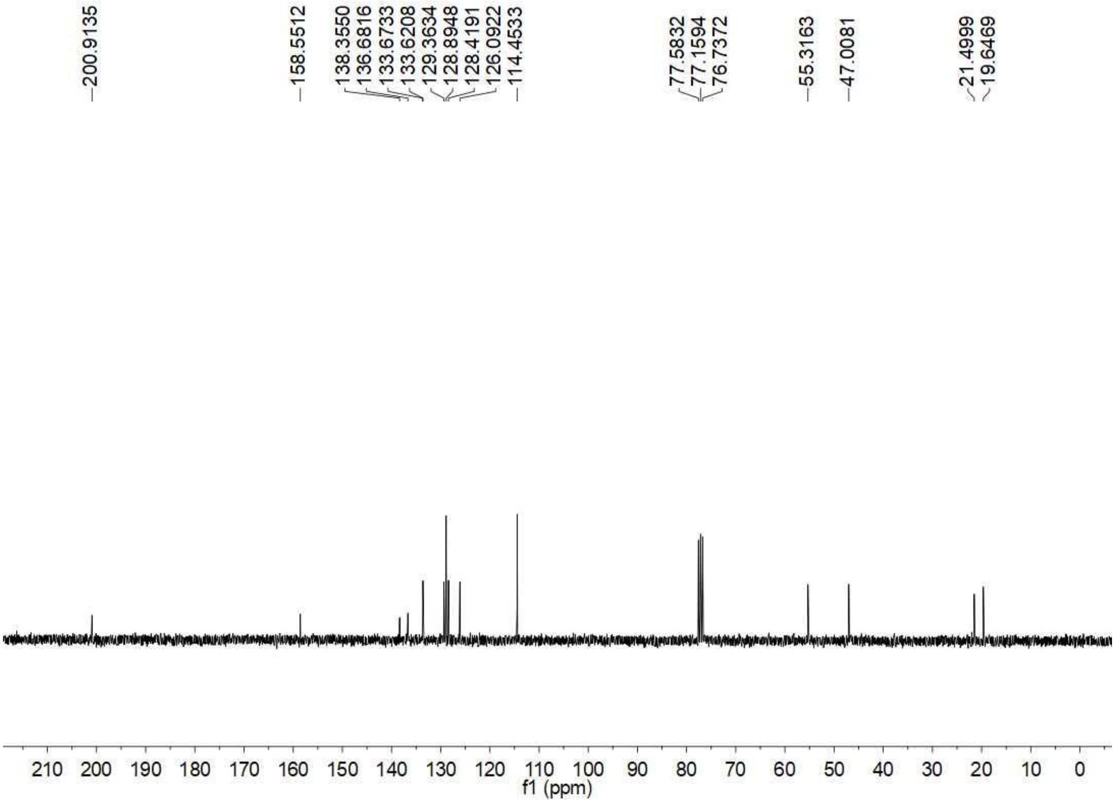

1-(3-Bromophenyl)-2-(4-methoxyphenyl)propan-1-one (3j)

^{1}H NMR (300 MHz, $CDCl_3$, 300 K)

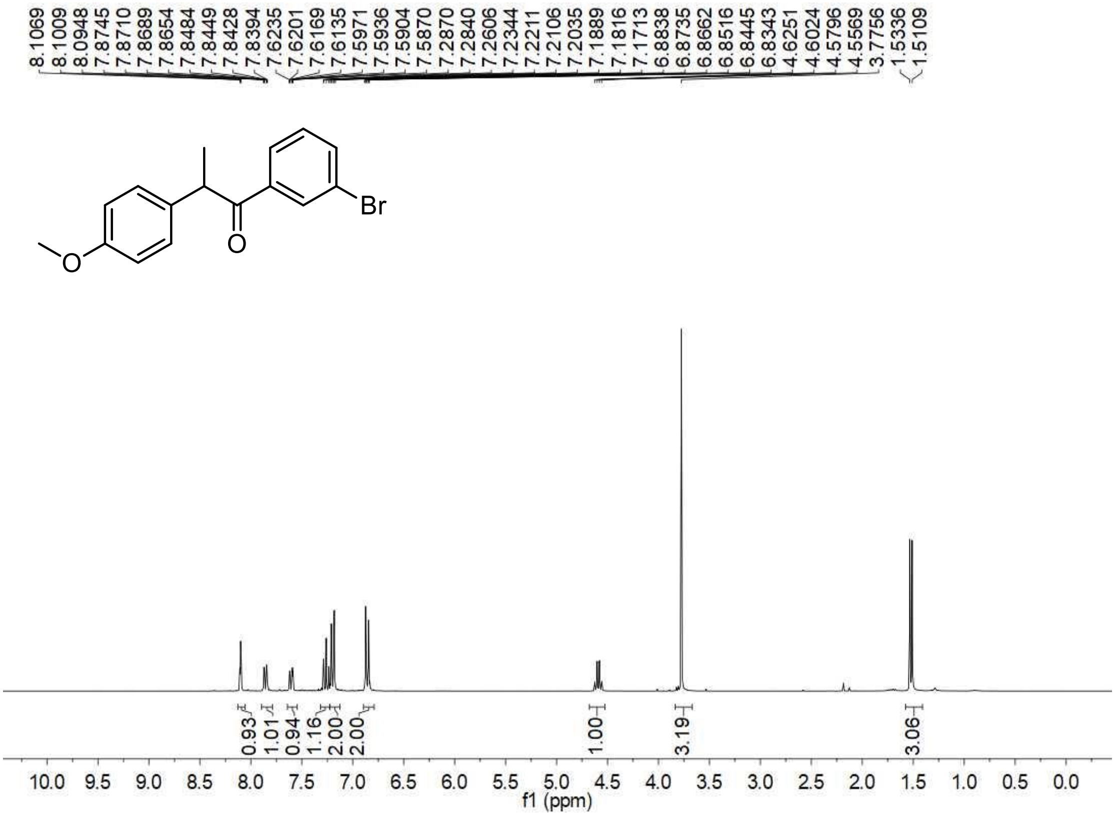

^{13}C NMR (75 MHz, $CDCl_3$, 300 K)

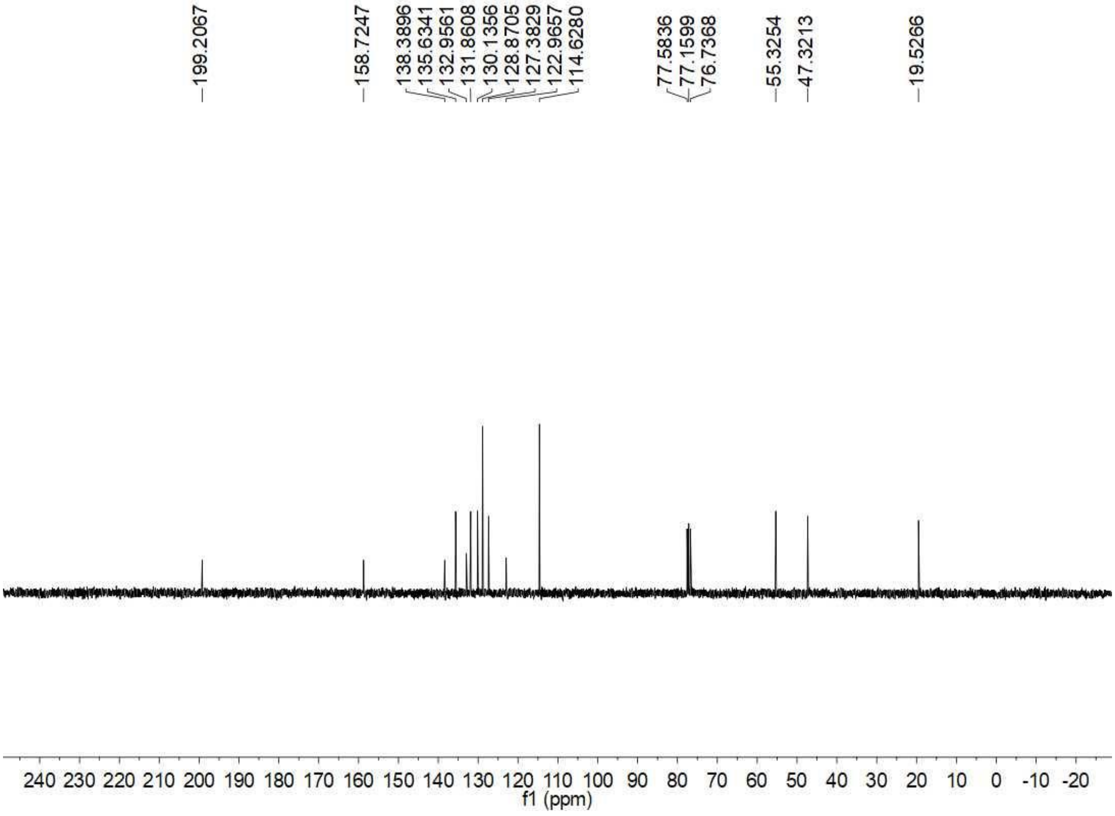

2-(4-Methoxyphenyl)-1-(3-(trifluoromethyl)phenyl)propan-1-one (3k)

^{1}H NMR (300 MHz, $CDCl_3$, 300 K)

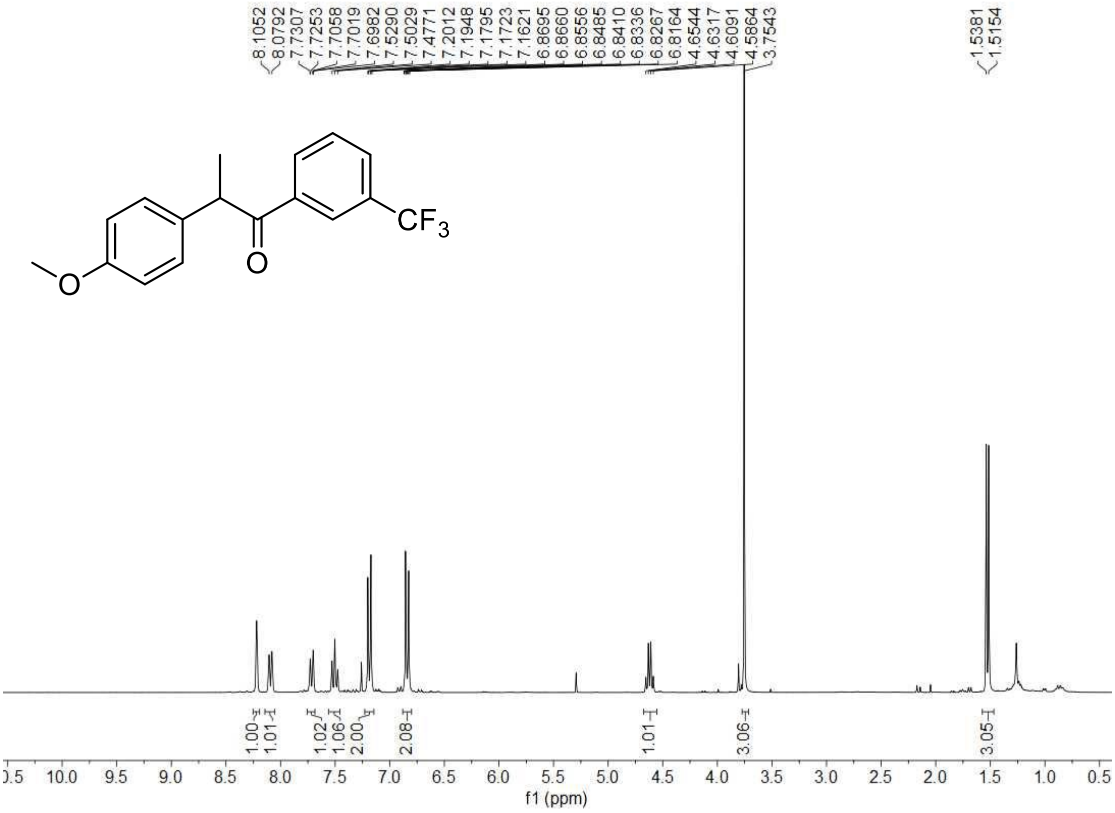

^{13}C NMR (100 MHz, $CDCl_3$, 300 K)

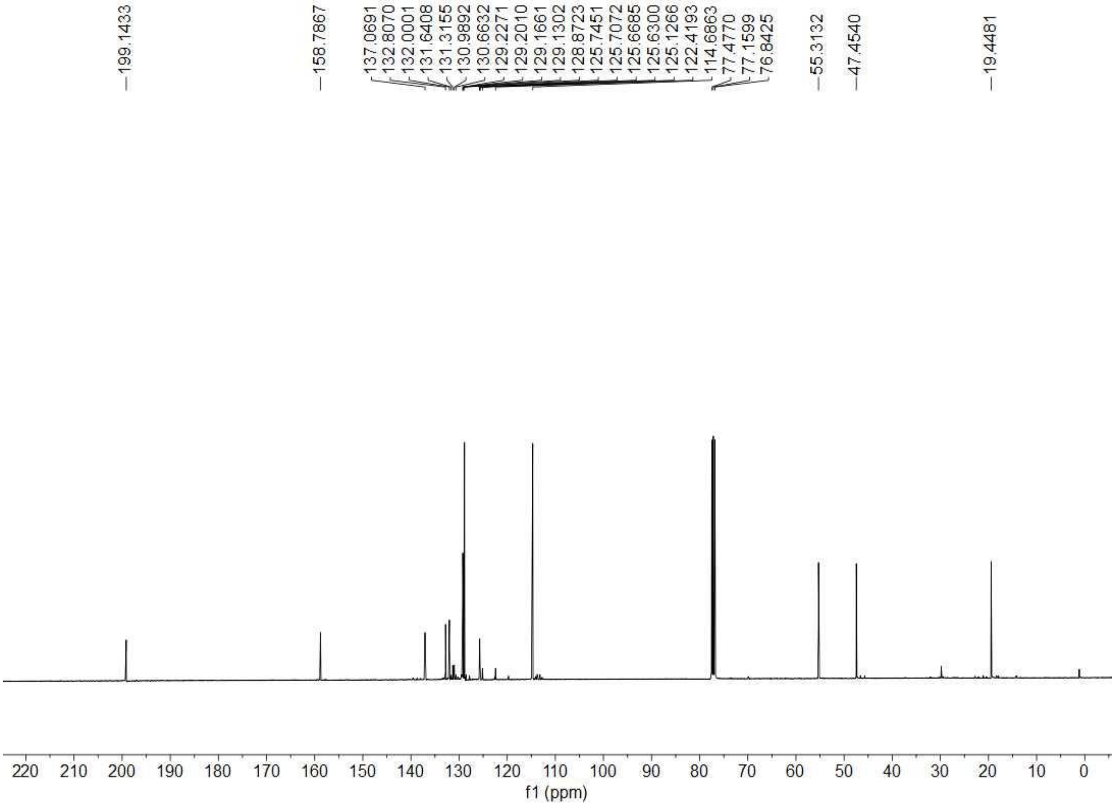

$^{19}F\{^{1}H\}$ NMR (282 MHz, $CDCl_3$, 300 K)

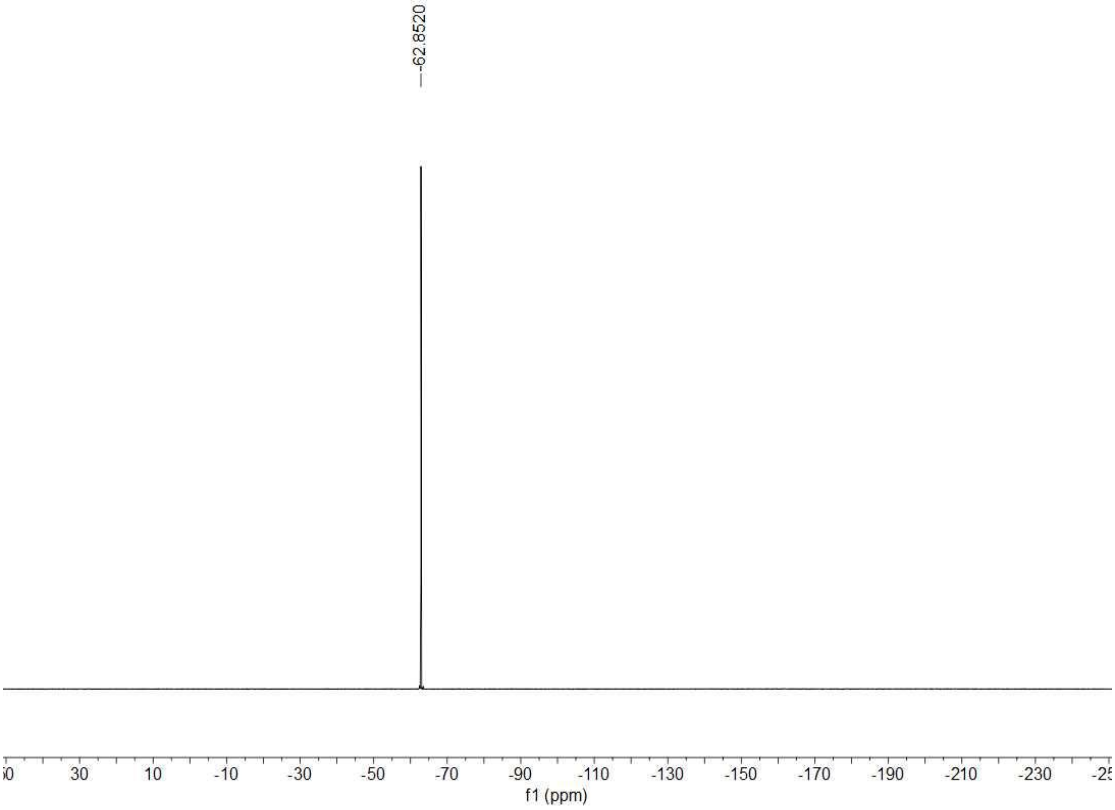

1-(2-Fluorophenyl)-2-(4-methoxyphenyl)propan-1-one (3l)

^{1}H NMR (300 MHz, $CDCl_3$, 300 K)

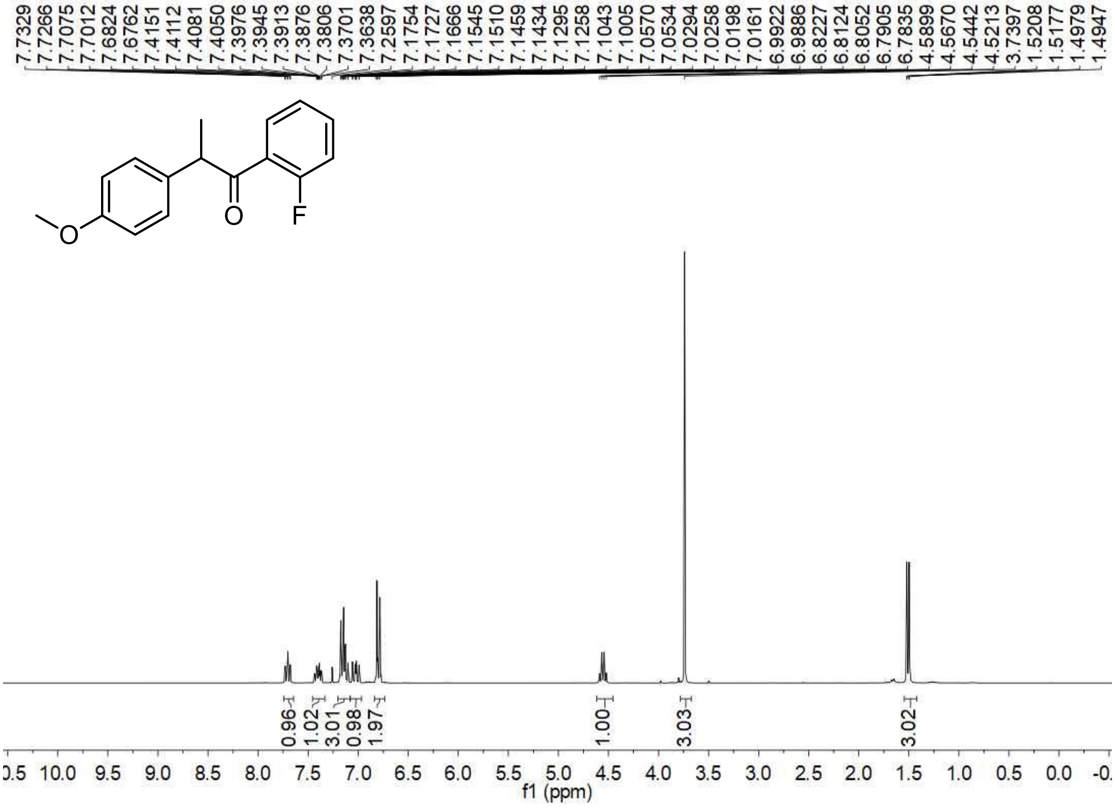

^{13}C NMR (75 MHz, $CDCl_3$, 300 K)

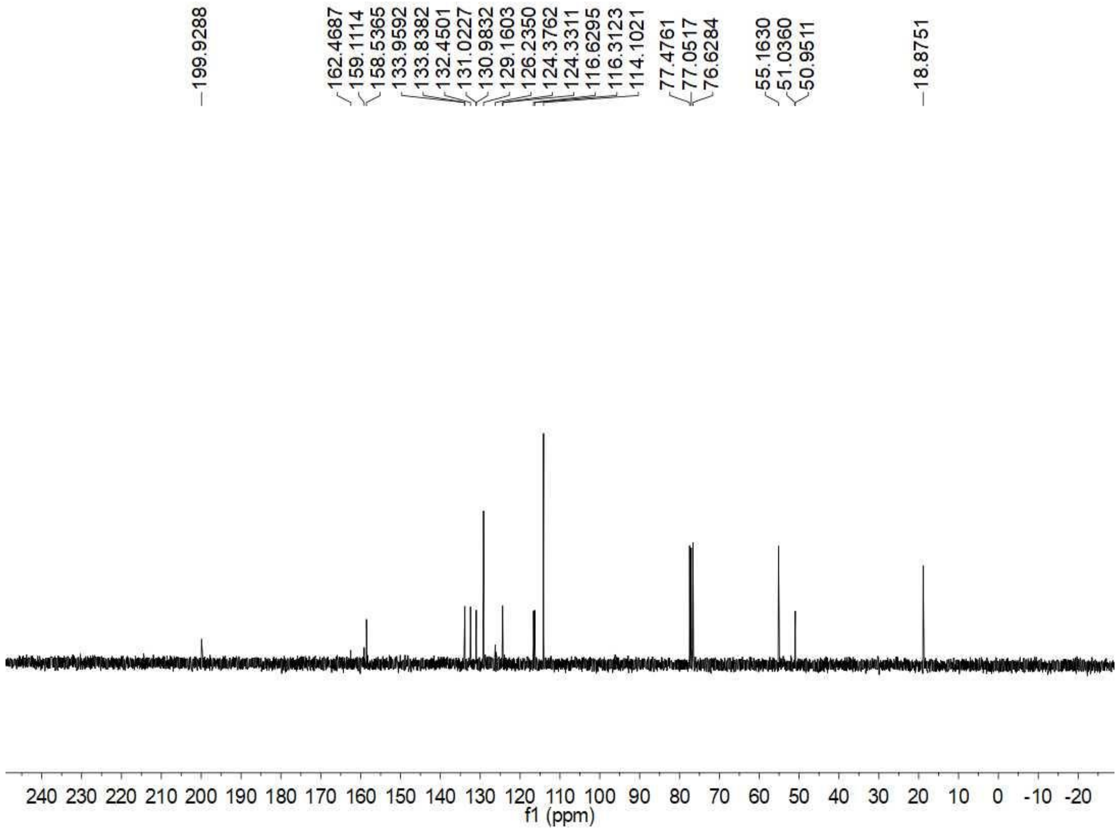

^{19}F {^{1}H} NMR (282 MHz, $CDCl_3$, 300 K)

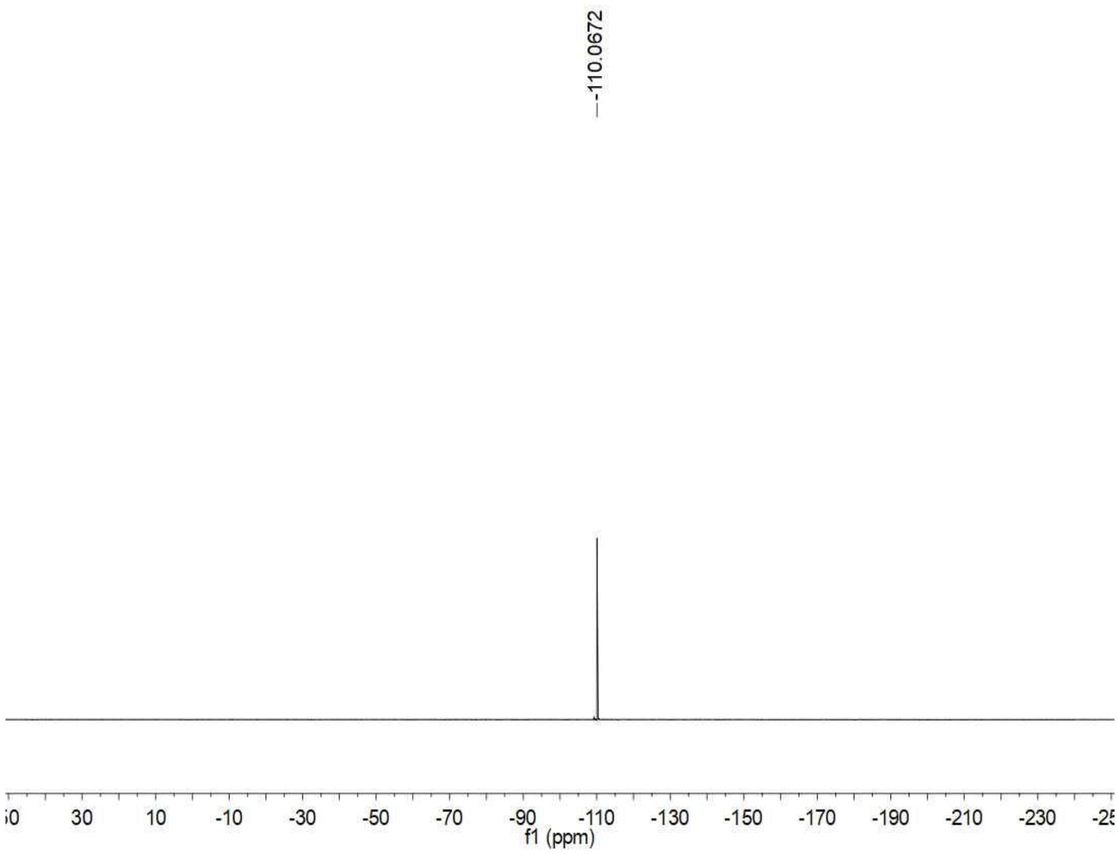

1-(2-Bromophenyl)-2-(4-methoxyphenyl)propan-1-one (3m)

^{1}H NMR (300 MHz, $CDCl_3$, 300 K)

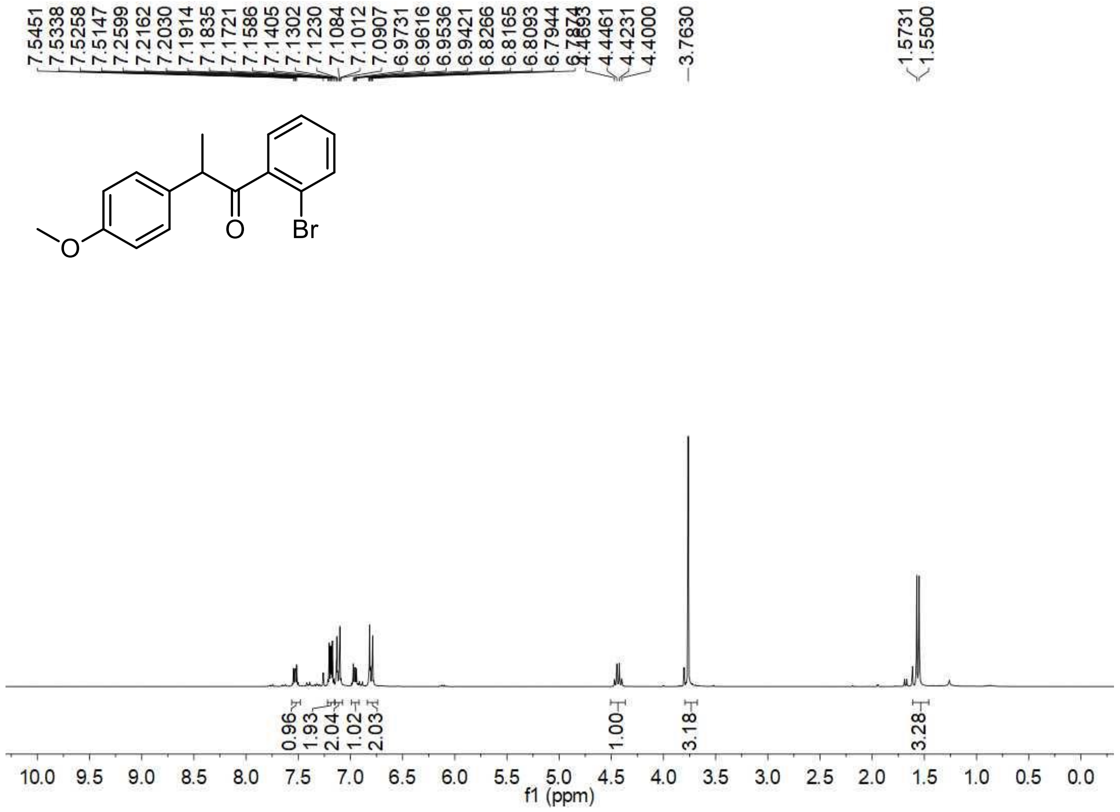

^{13}C NMR (75 MHz, $CDCl_3$, 300 K)

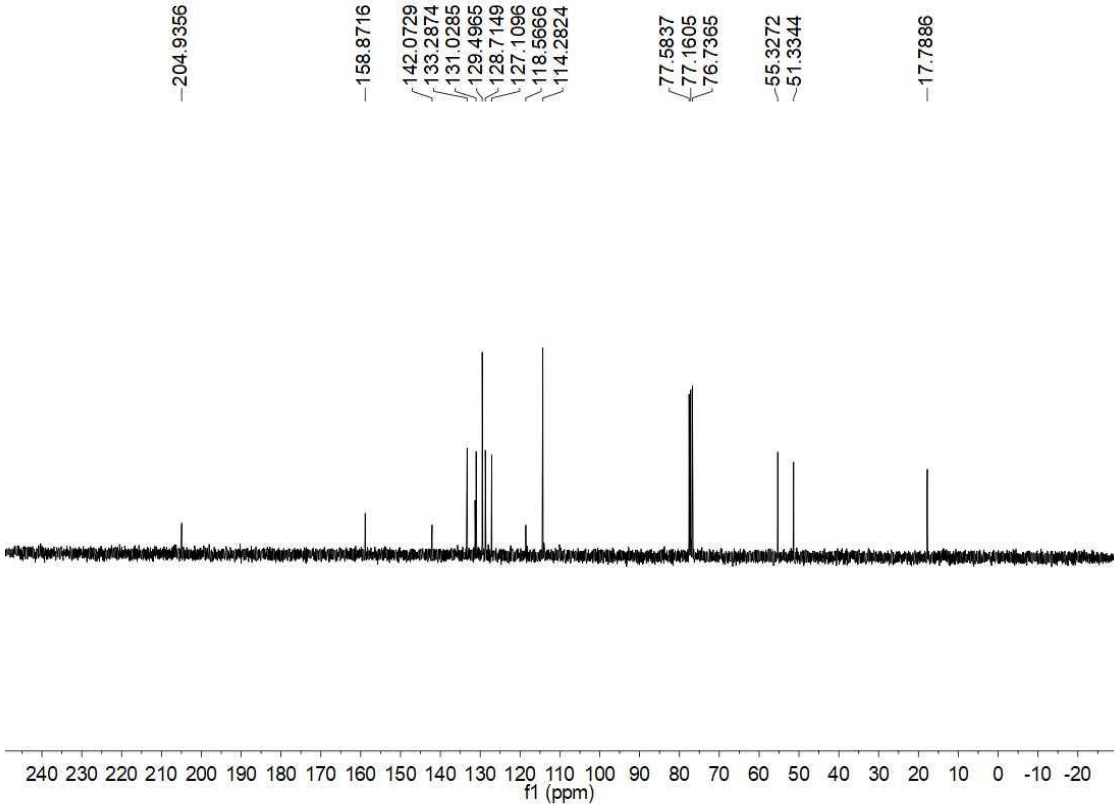

2-(4-Methoxyphenyl)-1-(naphthalen-1-yl)propan-1-one (3n)

^{1}H NMR (300 MHz, $CDCl_3$, 300 K)

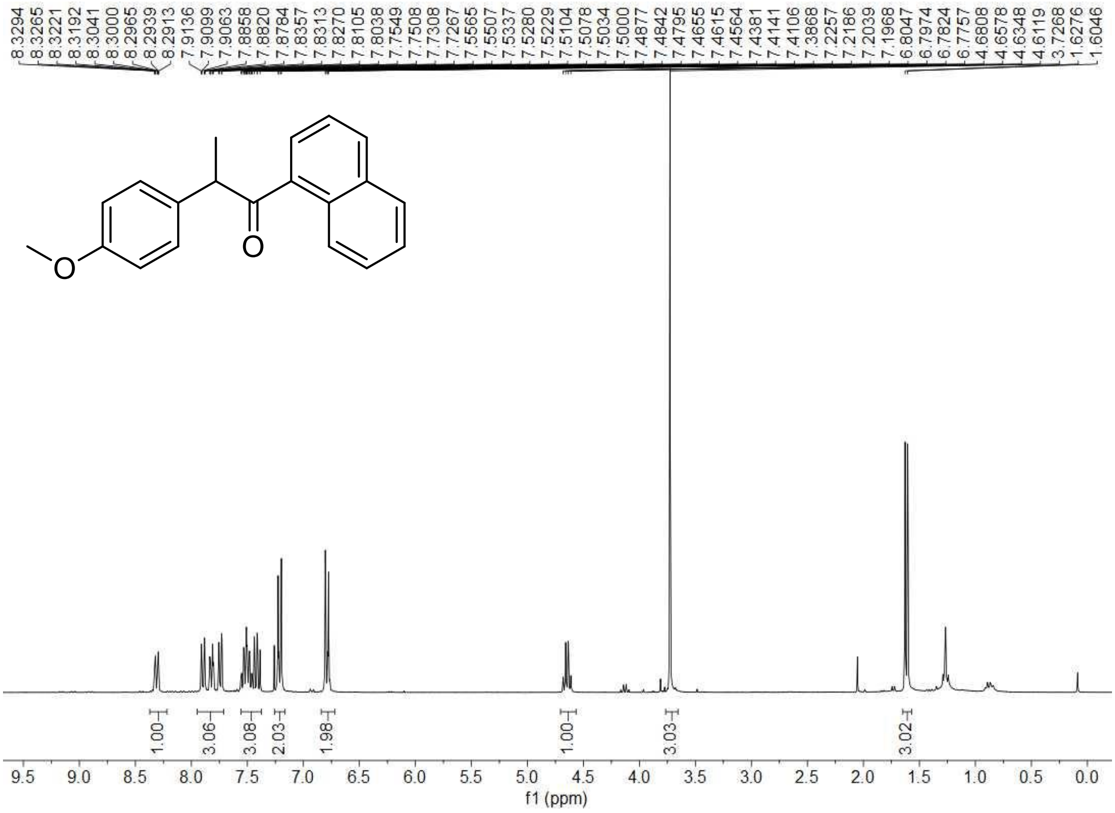

^{13}C NMR (75 MHz, $CDCl_3$, 300 K)

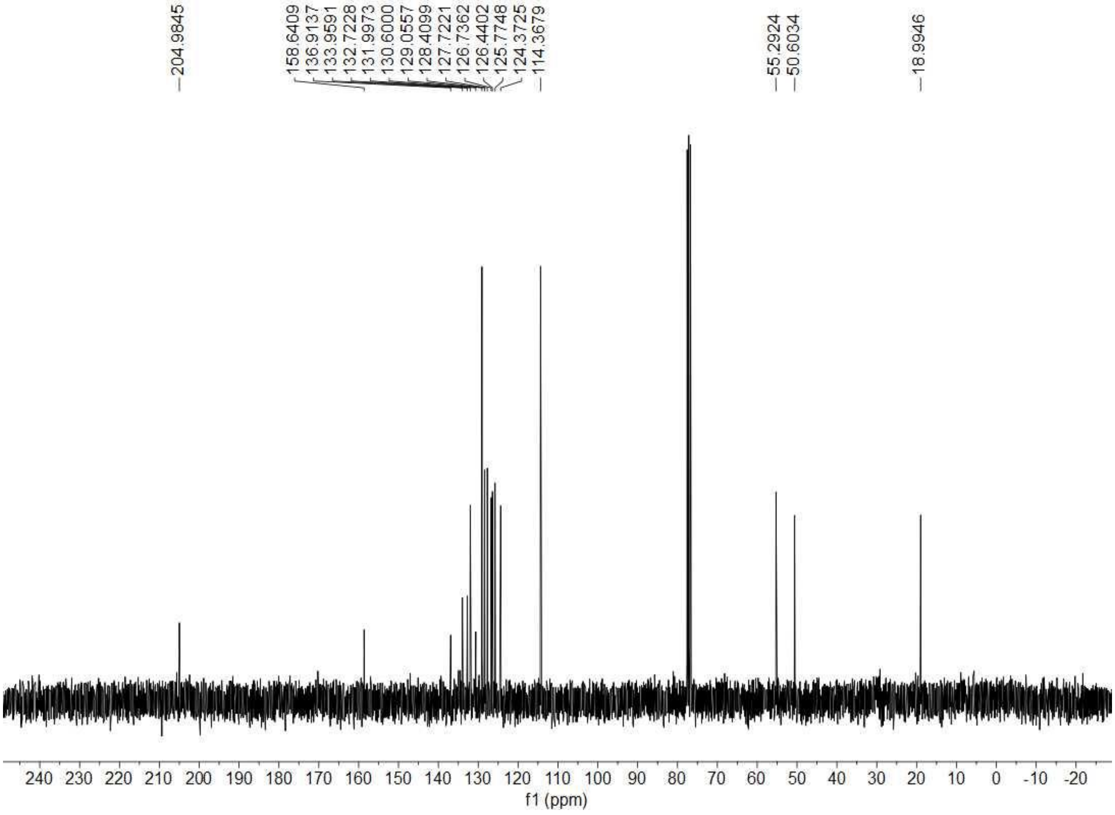

2-(4-Methoxyphenyl)-1-(naphthalen-2-yl)propan-1-one (3o)

^{1}H NMR (300 MHz, $CDCl_3$, 300 K)

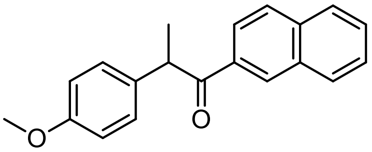

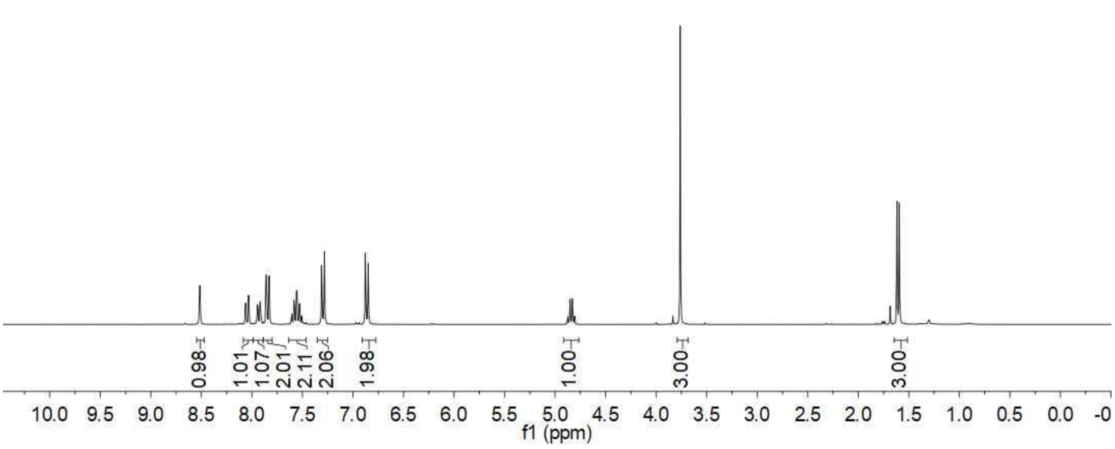

^{13}C NMR (75 MHz, $CDCl_3$, 300 K)

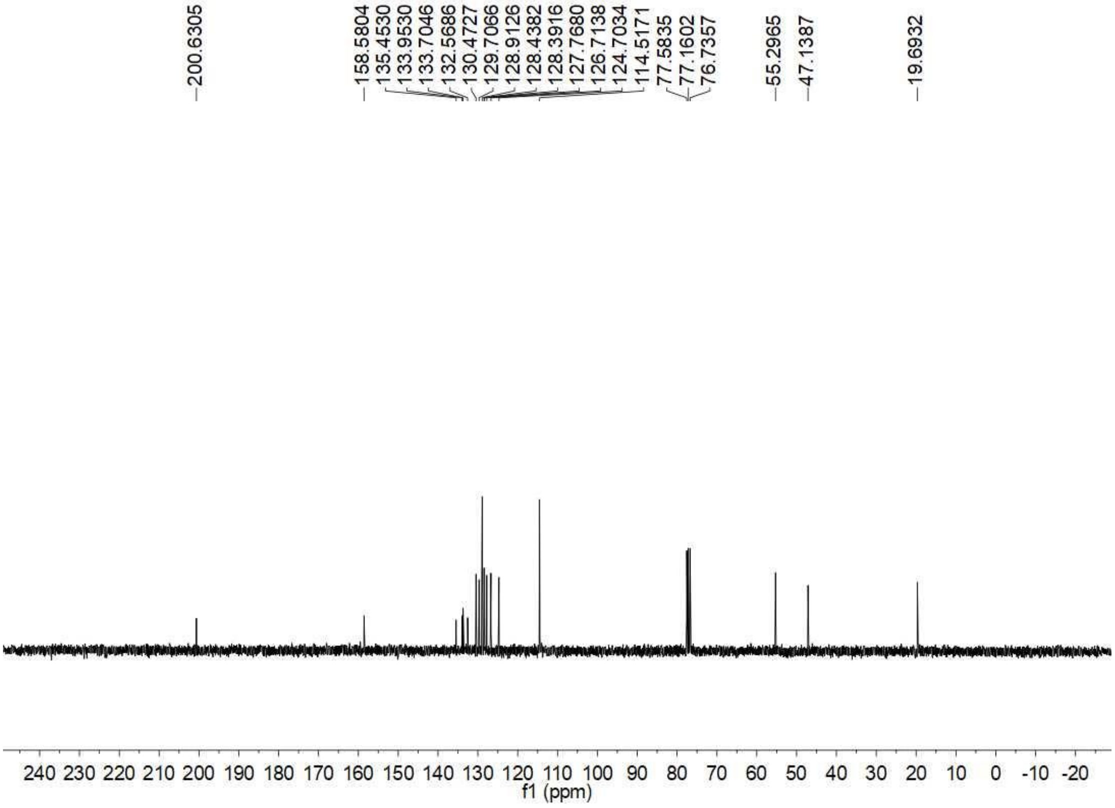

1-(Furan-2-yl)-2-(4-methoxyphenyl)propan-1-one (3p)

^{1}H NMR (300 MHz, $CDCl_3$, 300 K)

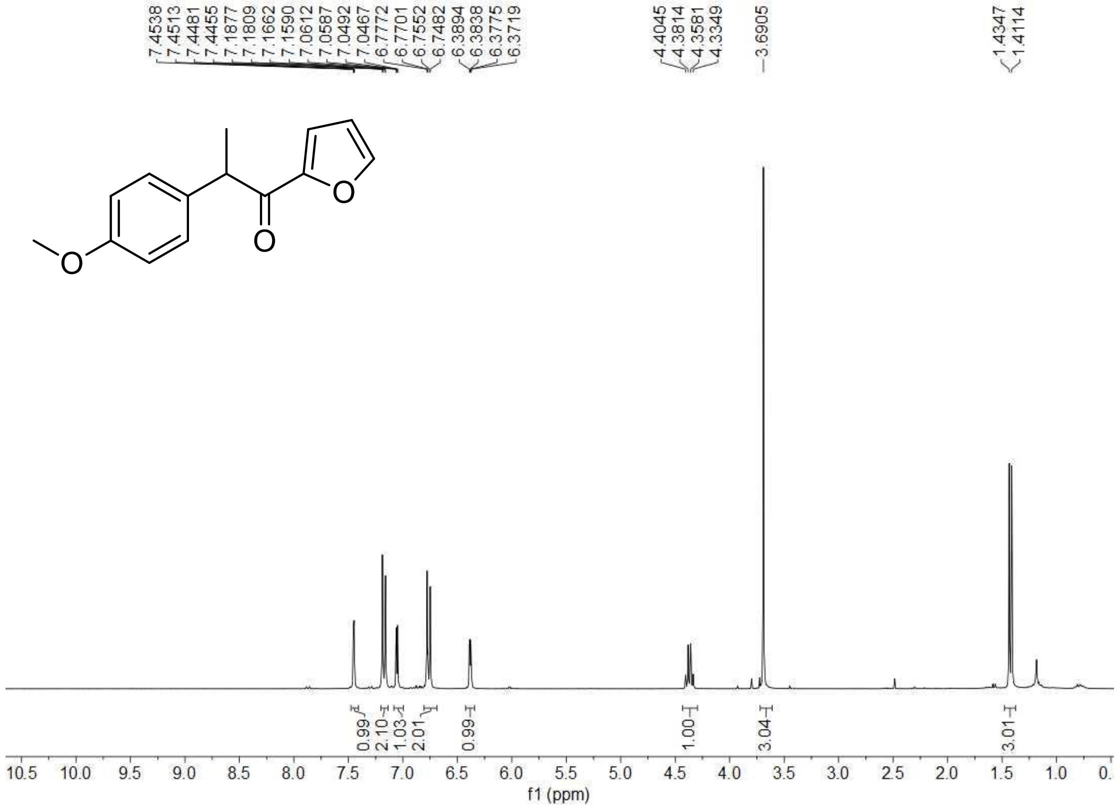

^{13}C NMR (75 MHz, $CDCl_3$, 300 K)

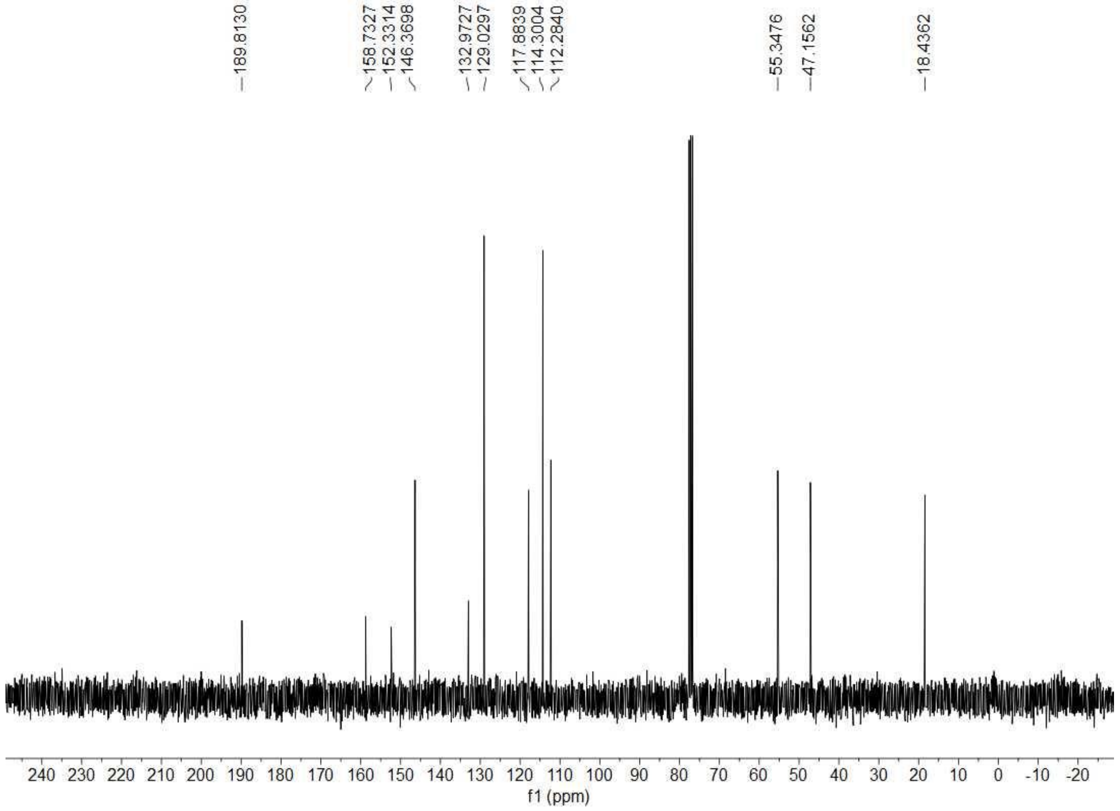

2-(4-Methoxyphenyl)-1-(thiophen-2-yl)propan-1-one (3q)

^{1}H NMR (300 MHz, $CDCl_3$, 300 K)

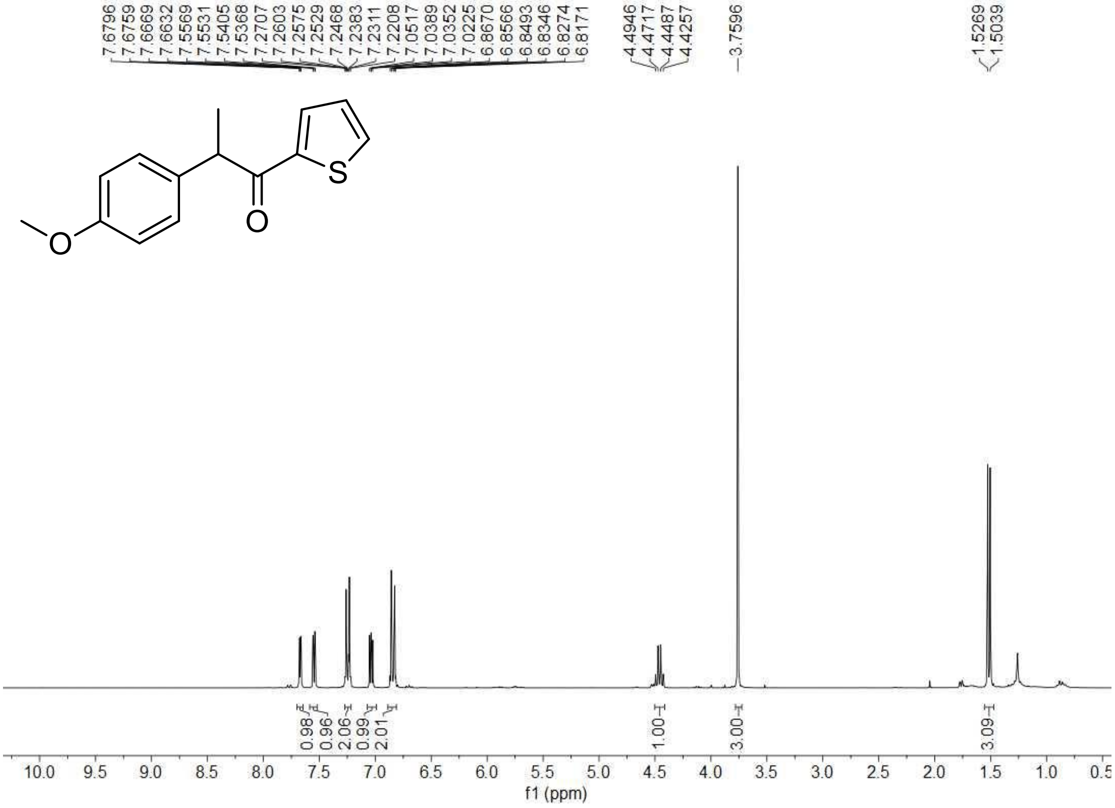

^{13}C NMR (75 MHz, $CDCl_3$, 300 K)

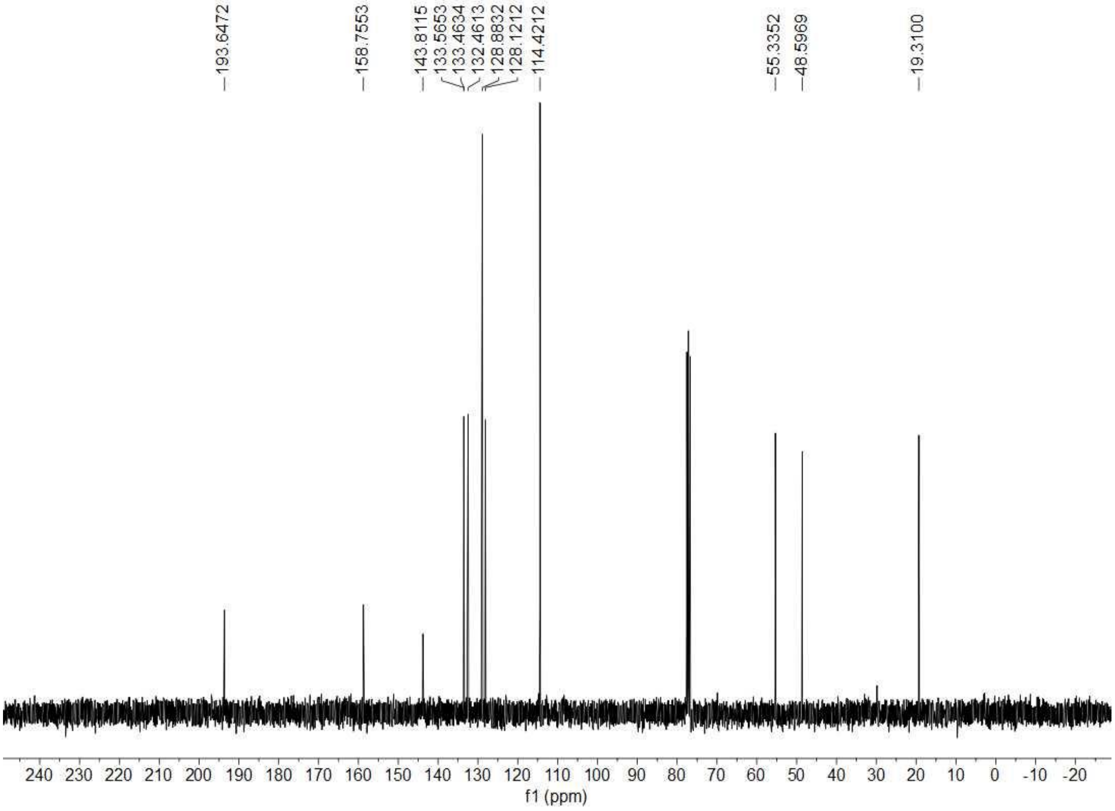

3-(4-Methoxyphenyl)-4-oxo-4-phenylbutyl acetate (3r)

^{1}H NMR (300 MHz, $CDCl_3$, 300 K)

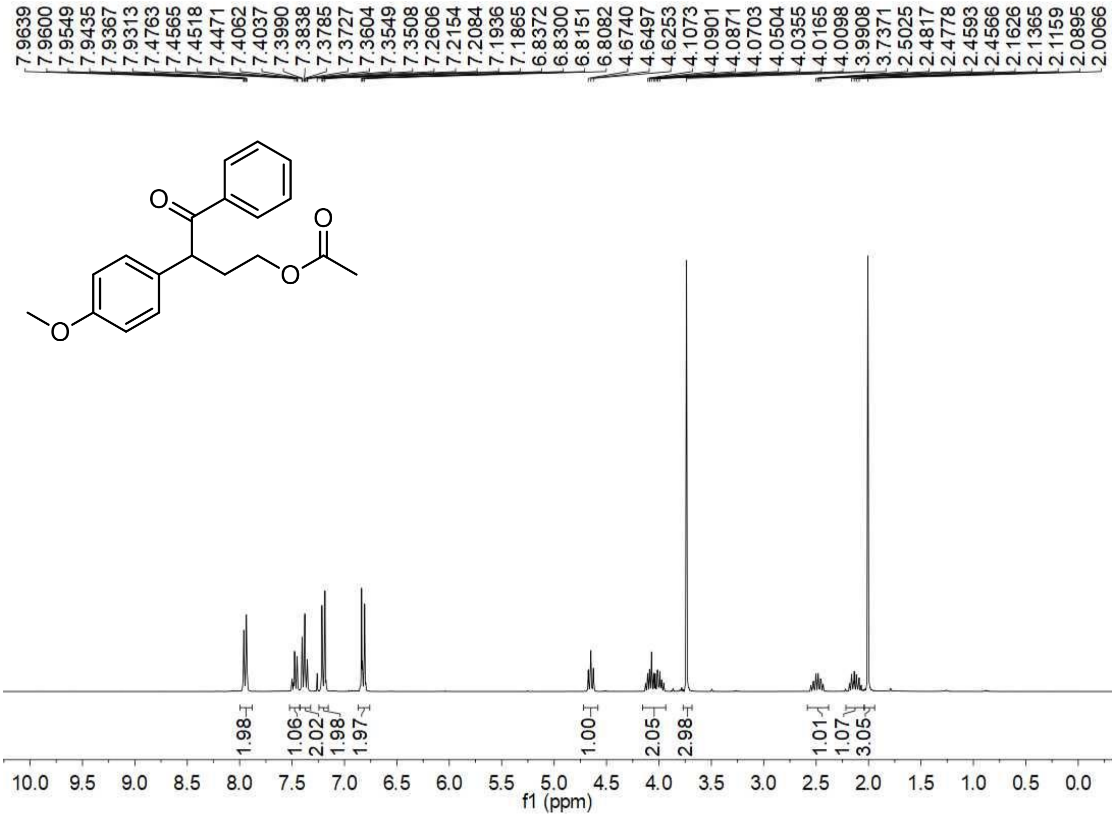

^{13}C NMR (75 MHz, $CDCl_3$, 300 K)

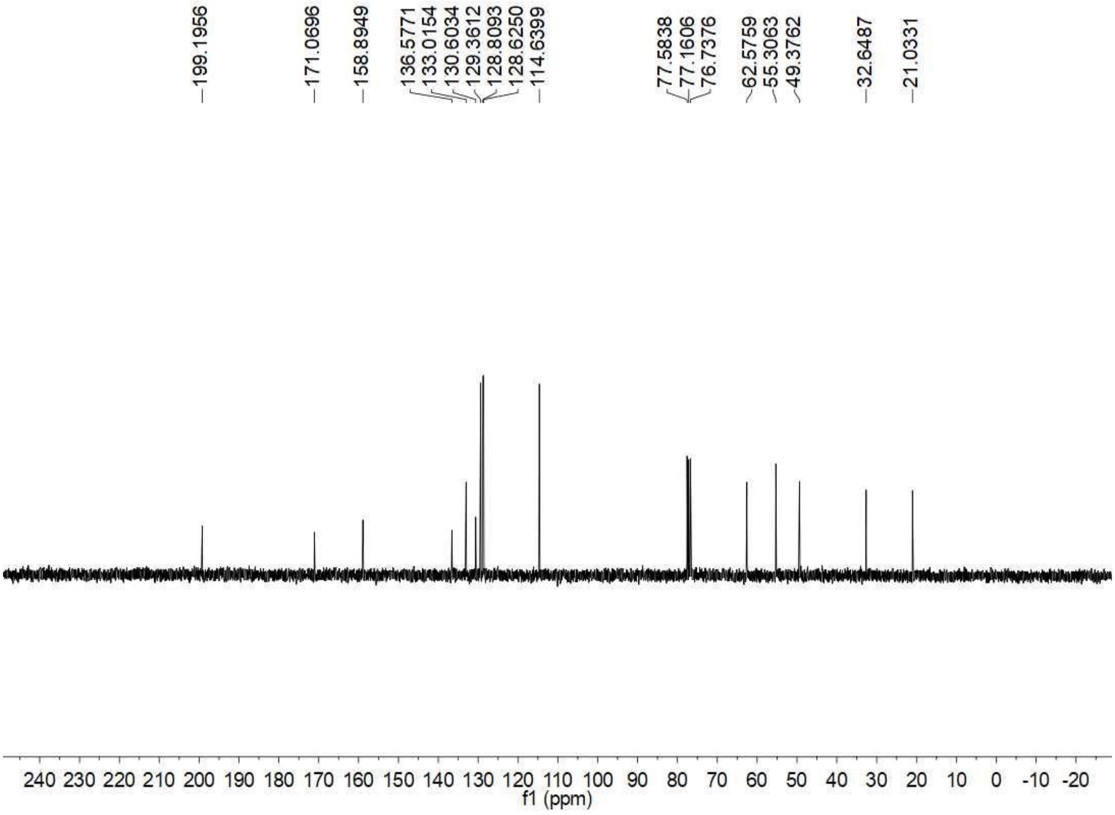

2-(4-Methoxyphenyl)-1-phenyl-5-(pyrrolidin-1-yl)pentane-1,5-dione (3s)

^{1}H NMR (300 MHz, $CDCl_3$, 300 K)

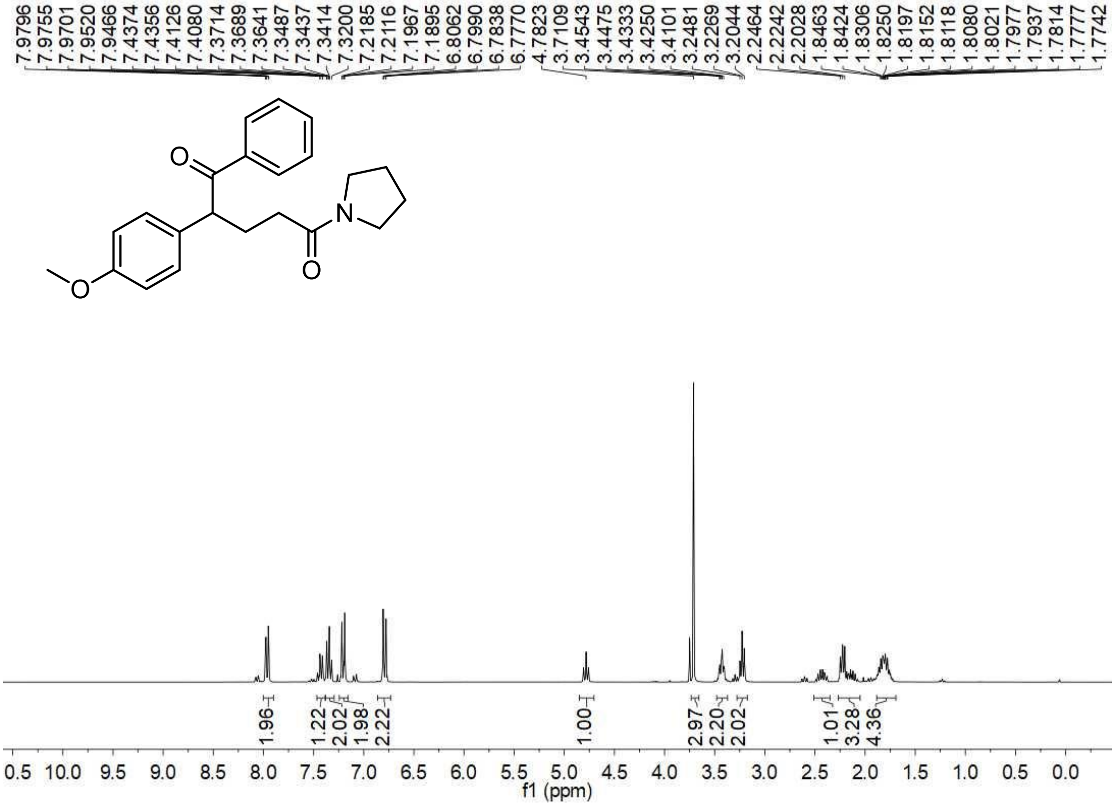

^{13}C NMR (75 MHz, $CDCl_3$, 300 K)

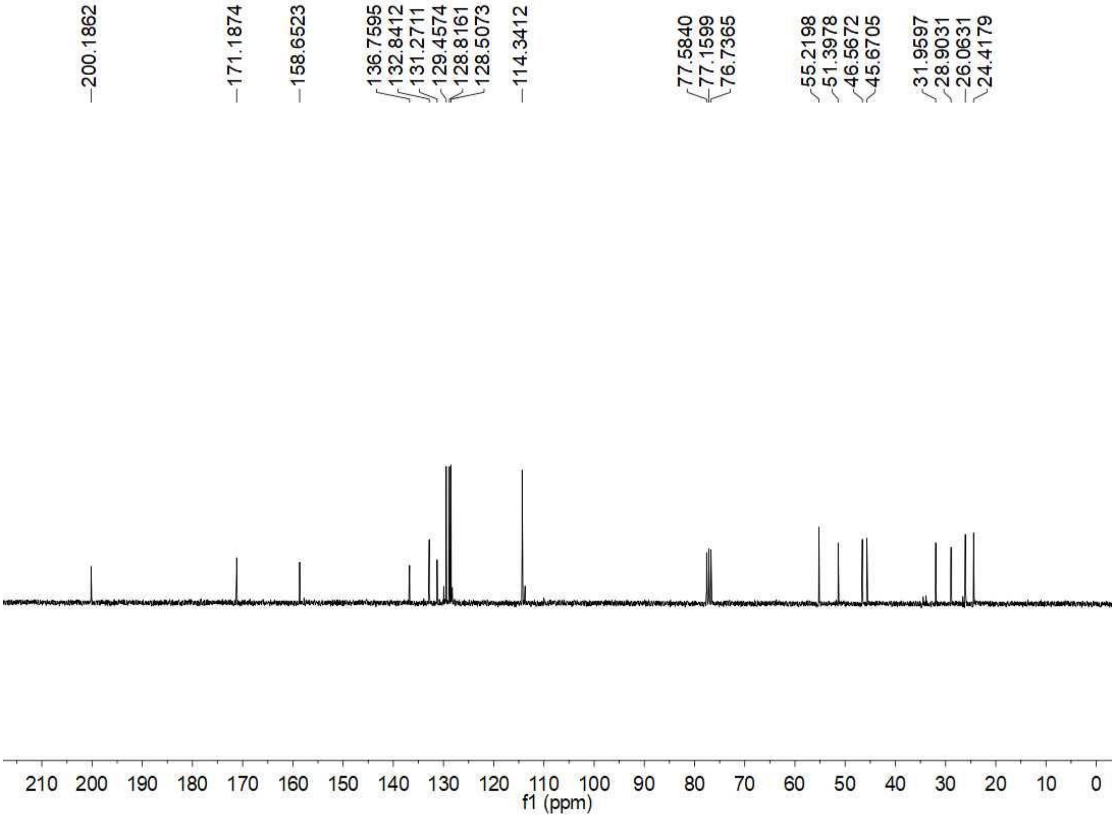

4-Azido-2-(4-methoxyphenyl)-1-phenylbutan-1-one (3t)

^{1}H NMR (300 MHz, $CDCl_3$, 300 K)

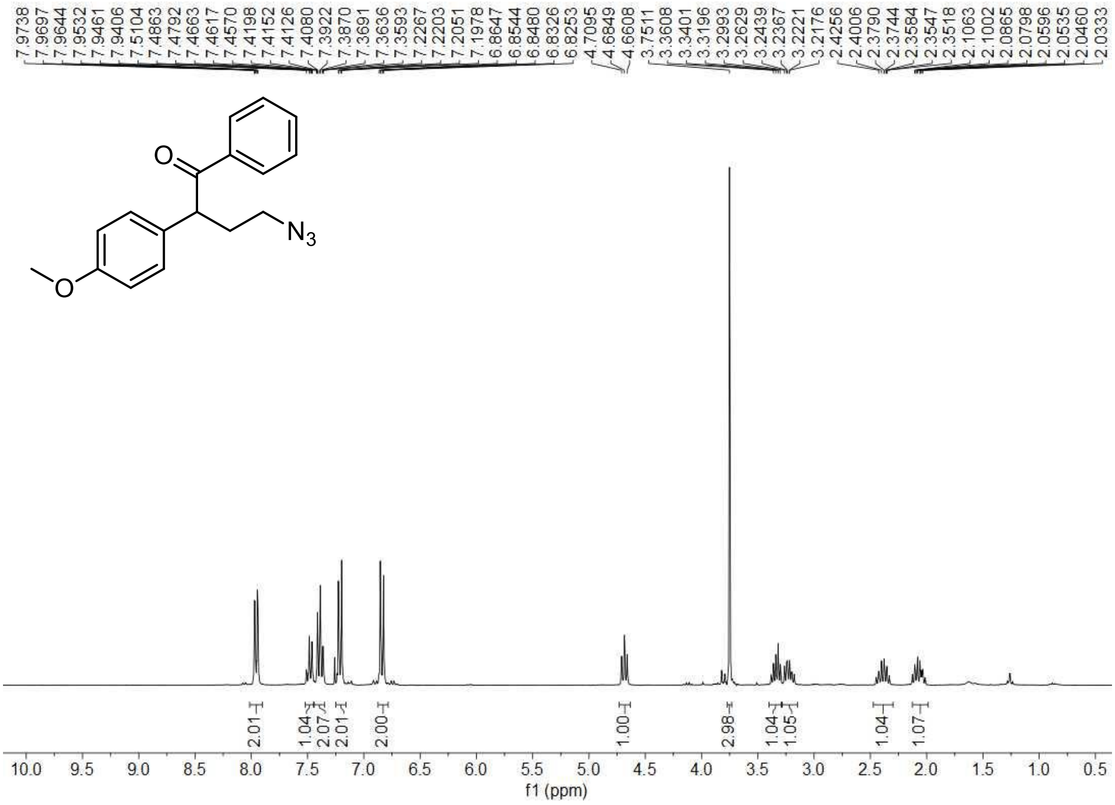

^{13}C NMR (75 MHz, $CDCl_3$, 300 K)

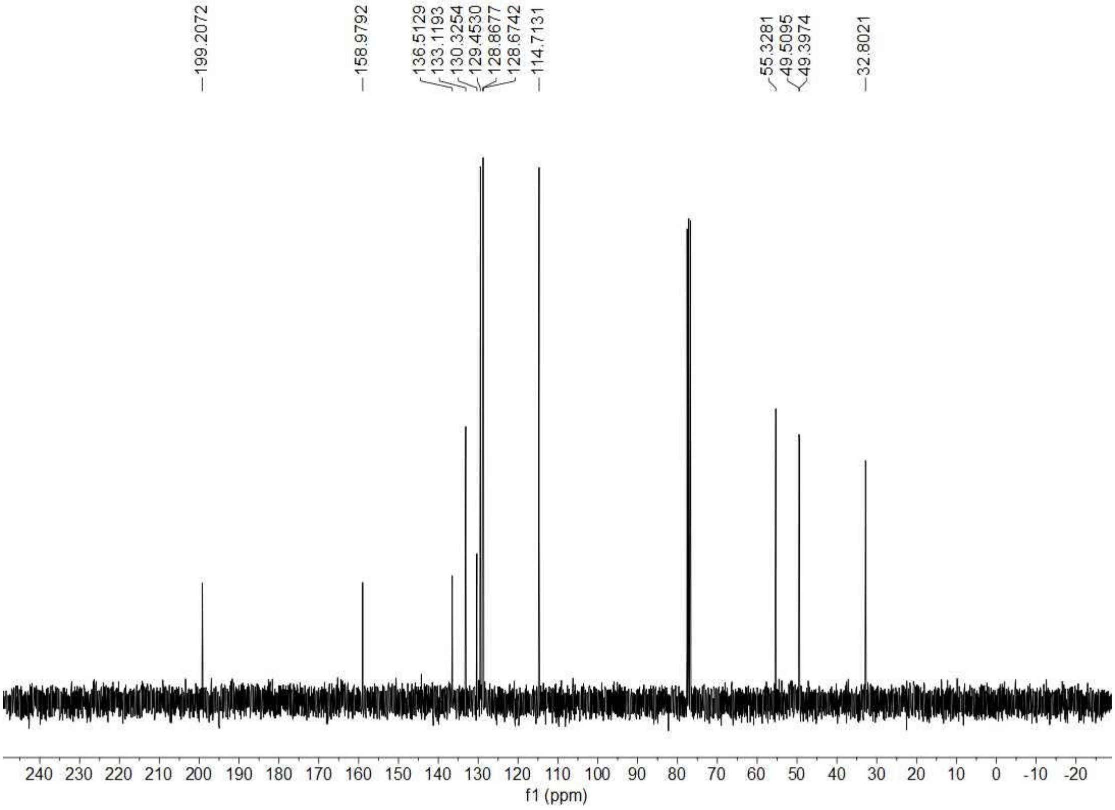

2-(4-Methoxyphenyl)-1-phenylpentane-1,4-dione (3u)

^{1}H NMR (300 MHz, $CDCl_3$, 300 K)

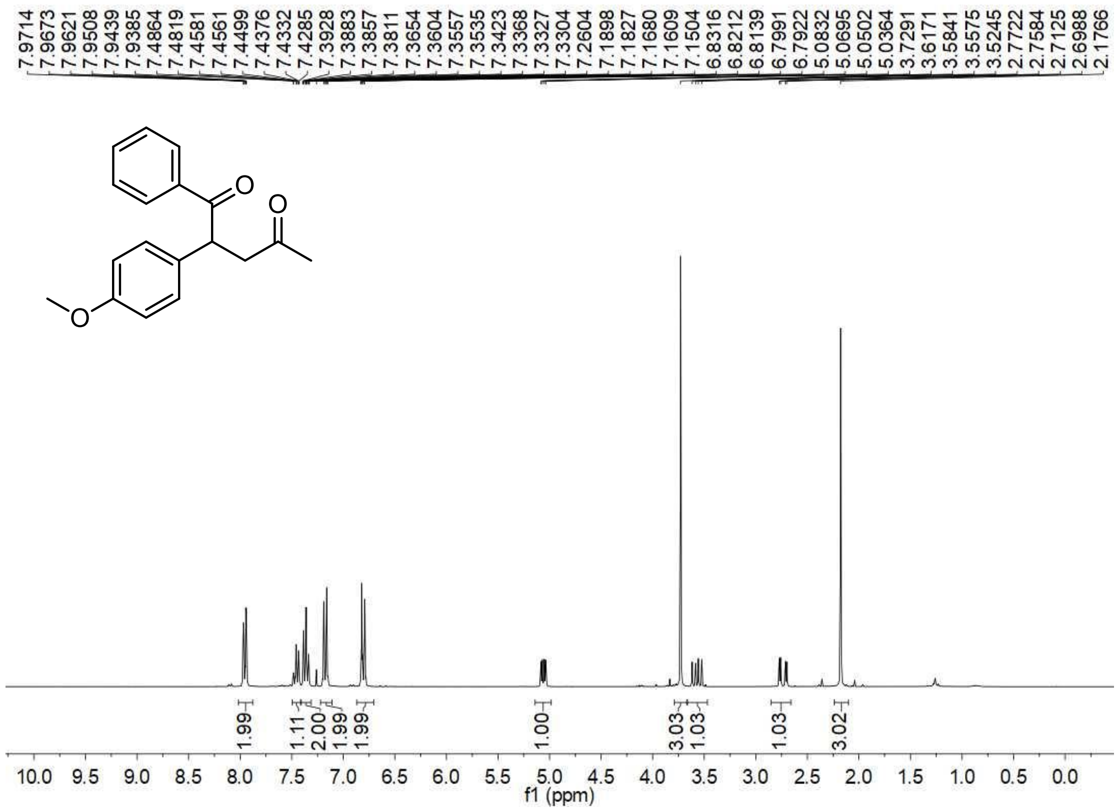

^{13}C NMR (75 MHz, $CDCl_3$, 300 K)

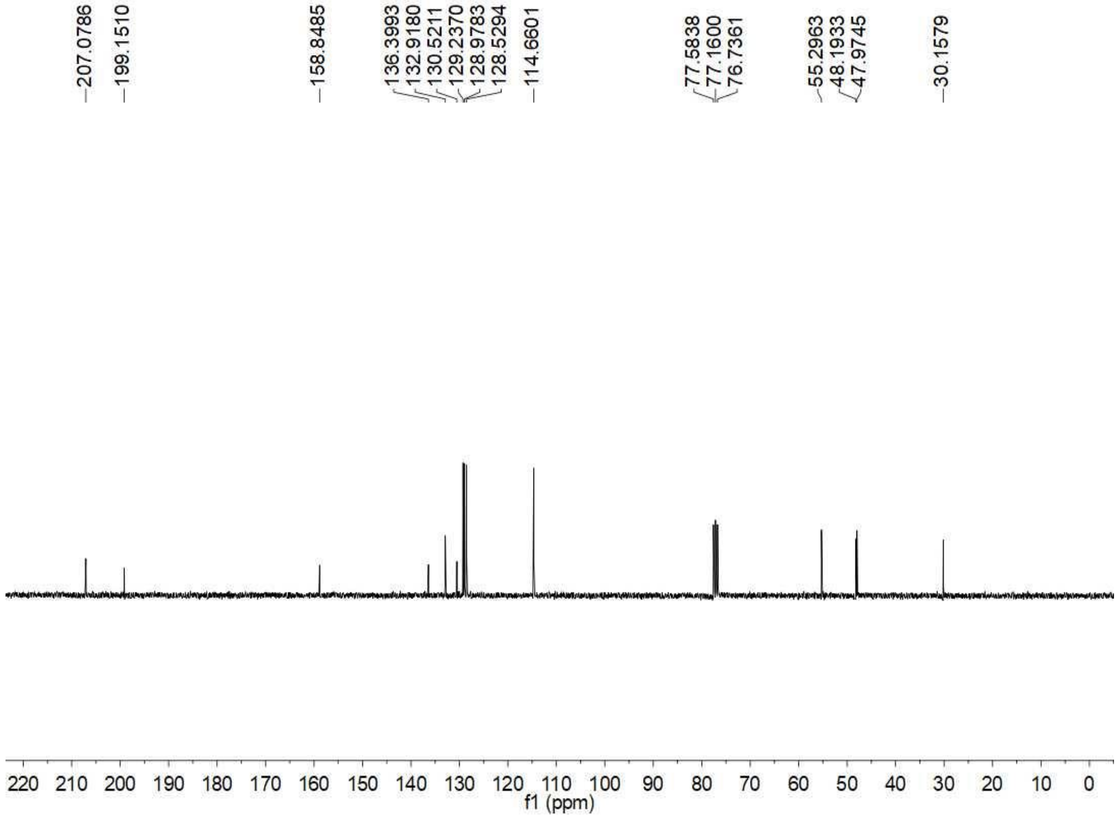

3-Methoxy-2-(4-methoxyphenyl)-1-phenylpropan-1-one (3v)

^{1}H NMR (300 MHz, $CDCl_3$, 300 K)

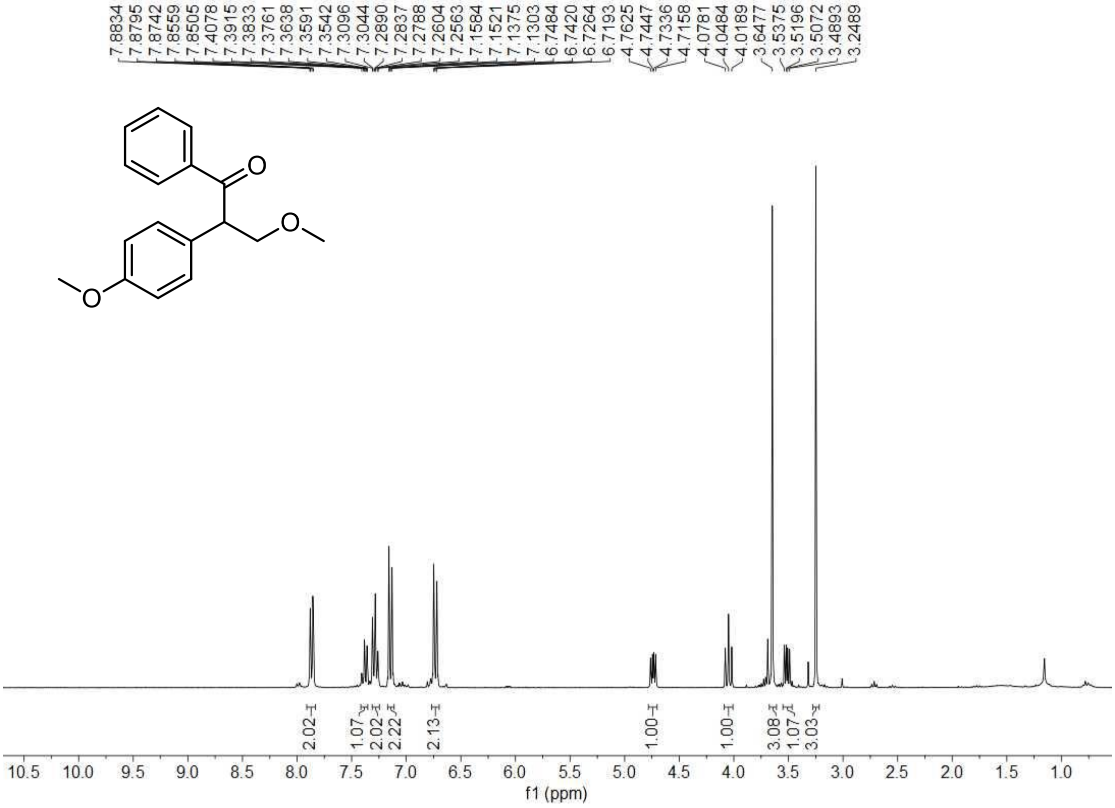

^{13}C NMR (75 MHz, $CDCl_3$, 300 K)

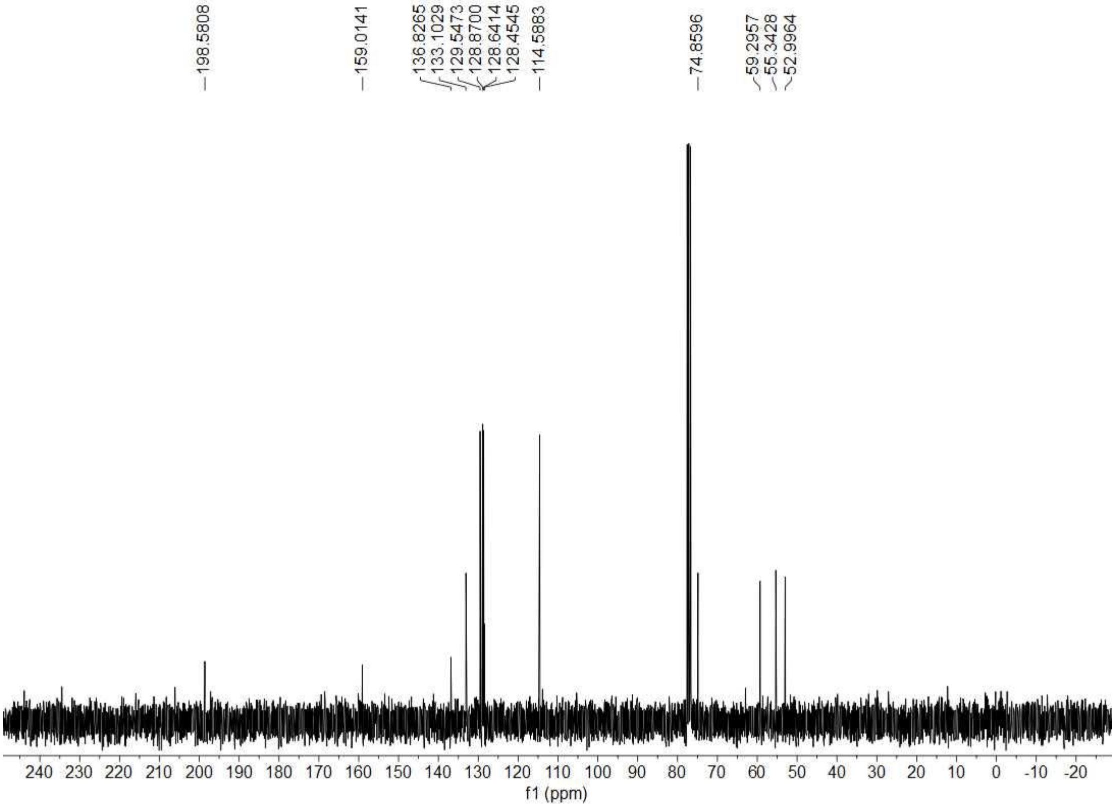

Methyl 2-(4-methoxyphenyl)-3-oxo-3-phenylpropanoate (3w)

^{1}H NMR (300 MHz, $CDCl_3$, 300 K)

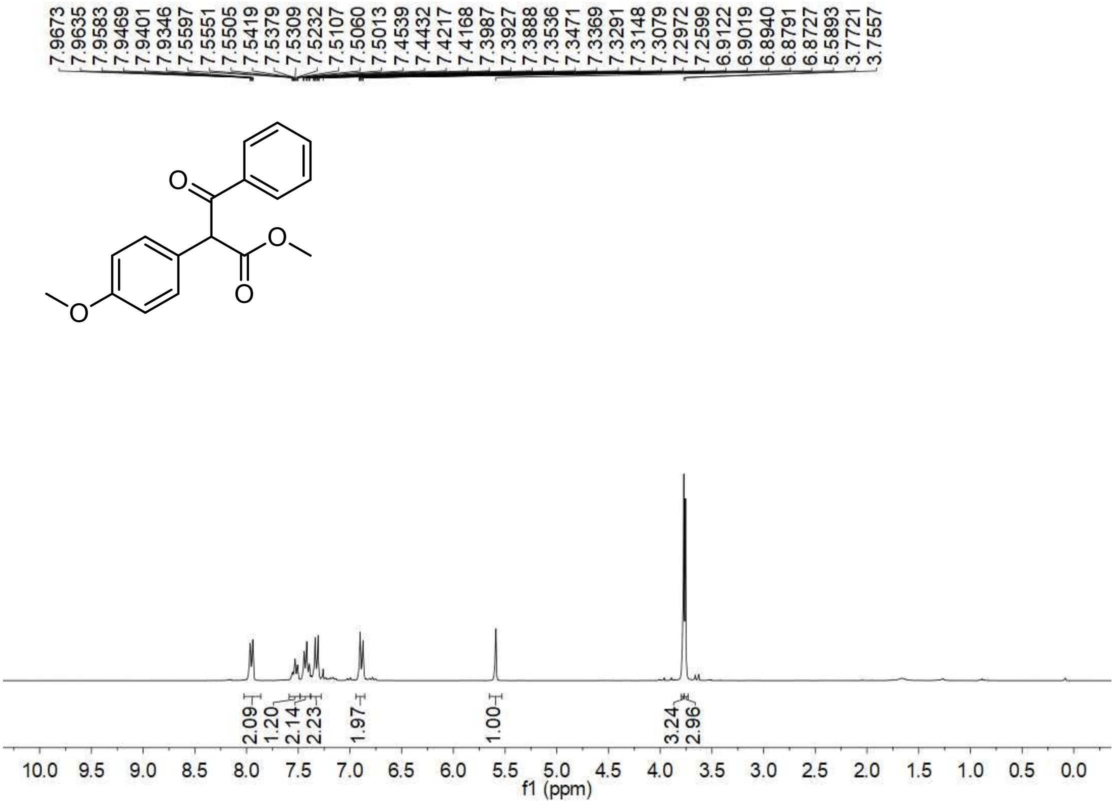

^{13}C NMR (75 MHz, $CDCl_3$, 300 K)

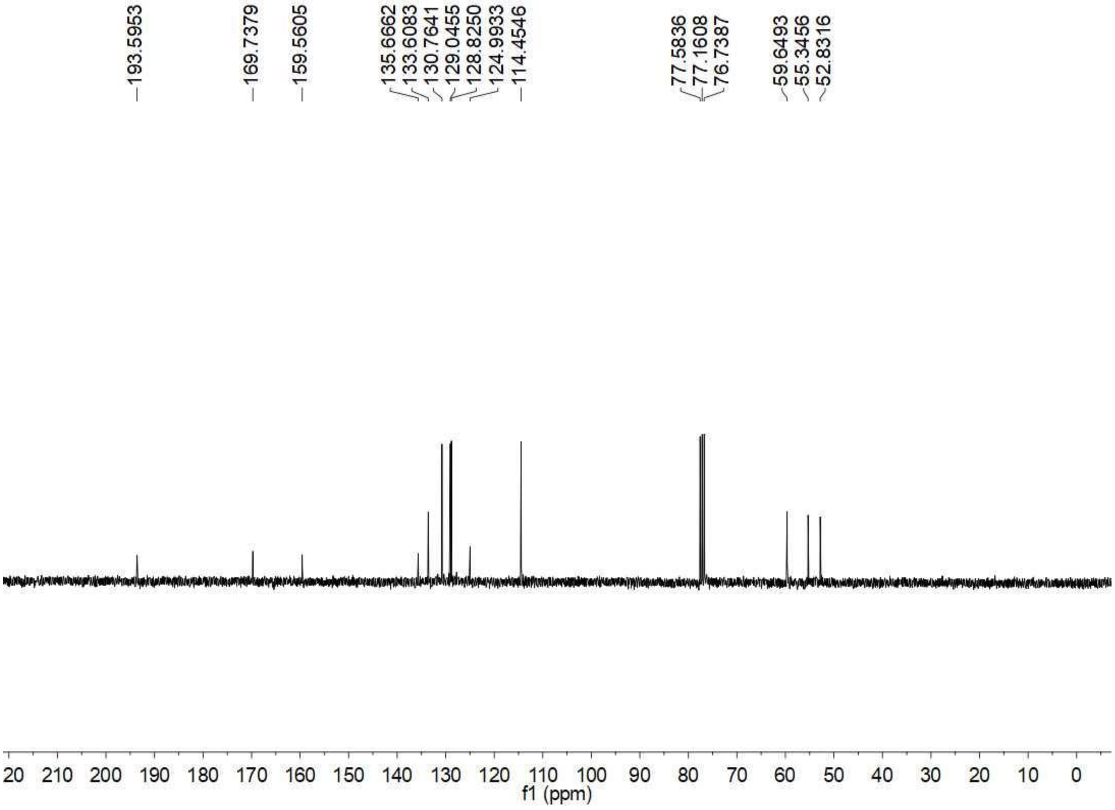

(2,3-Dihydrobenzofuran-3-yl)(phenyl)methanone (3x)

^{1}H NMR (300 MHz, $CDCl_3$, 300 K)

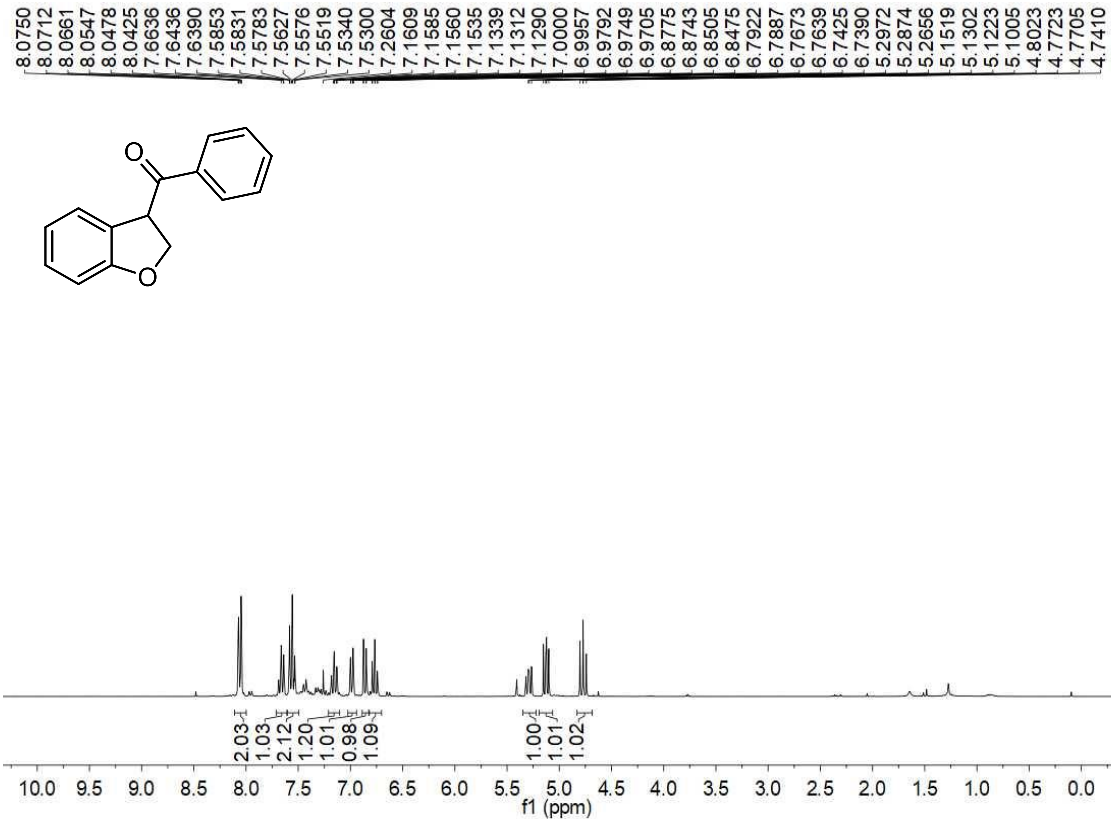

^{13}C NMR (75 MHz, $CDCl_3$, 300 K)

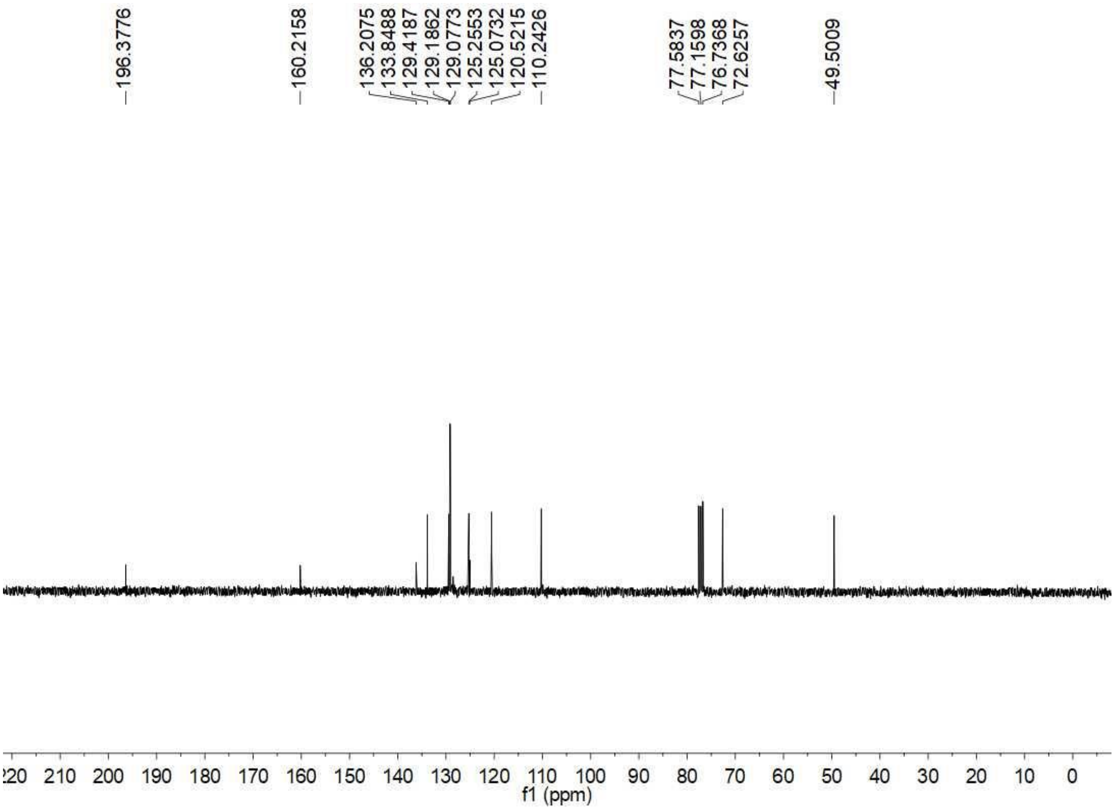

Chroman-4-yl(phenyl)methanone (3y)

^{1}H NMR (300 MHz, $CDCl_3$, 300 K)

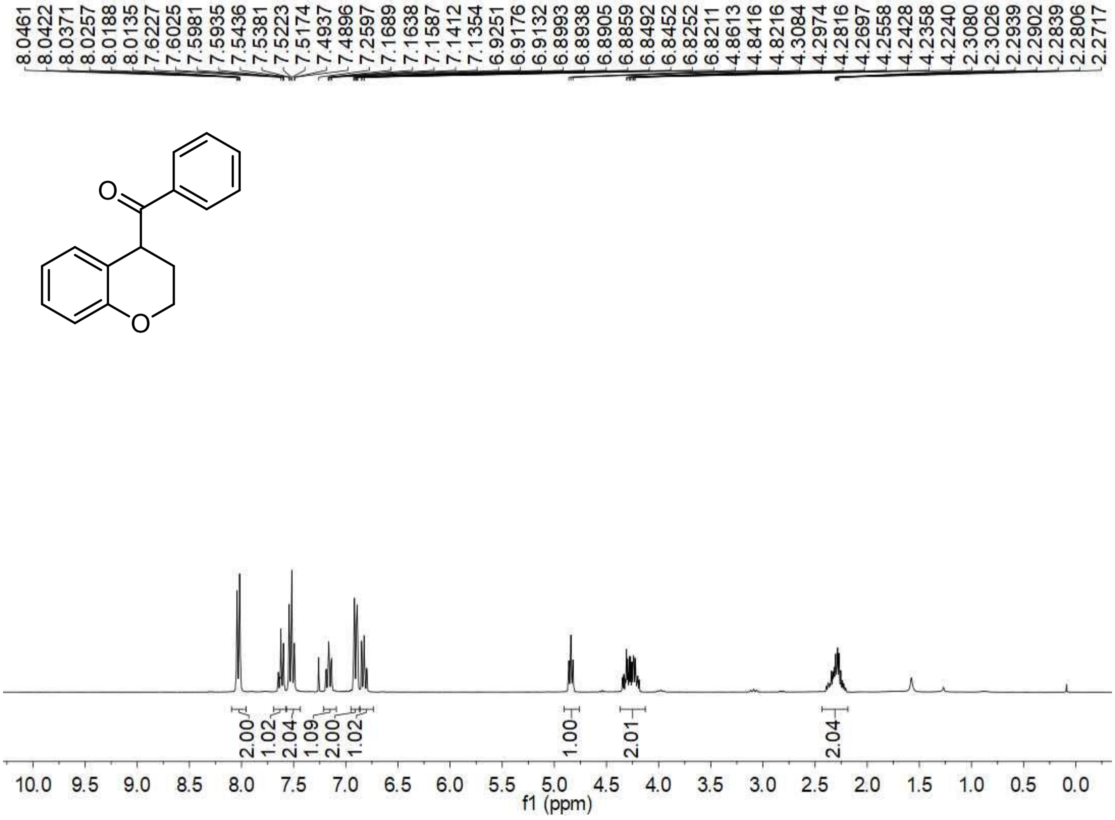

^{13}C NMR (75 MHz, $CDCl_3$, 300 K)

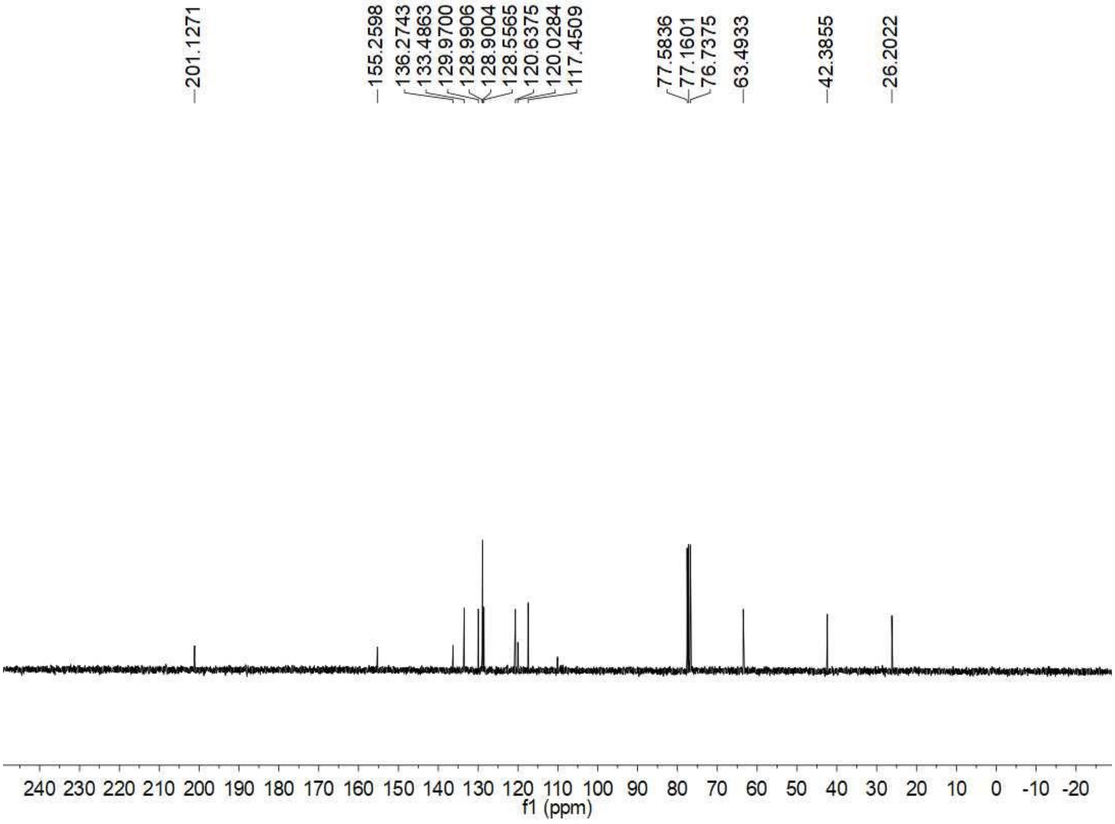

2-(4-Phenoxyphenyl)-1-phenylpropan-1-one (3z)

^{1}H NMR (300 MHz, $CDCl_3$, 300 K)

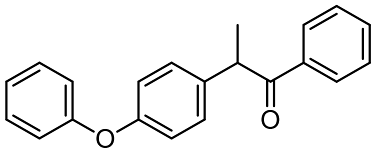

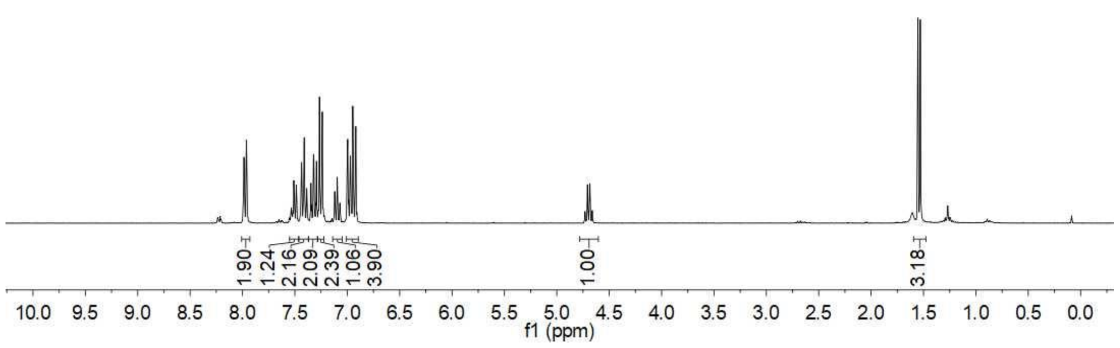

^{13}C NMR (75 MHz, $CDCl_3$, 300 K)

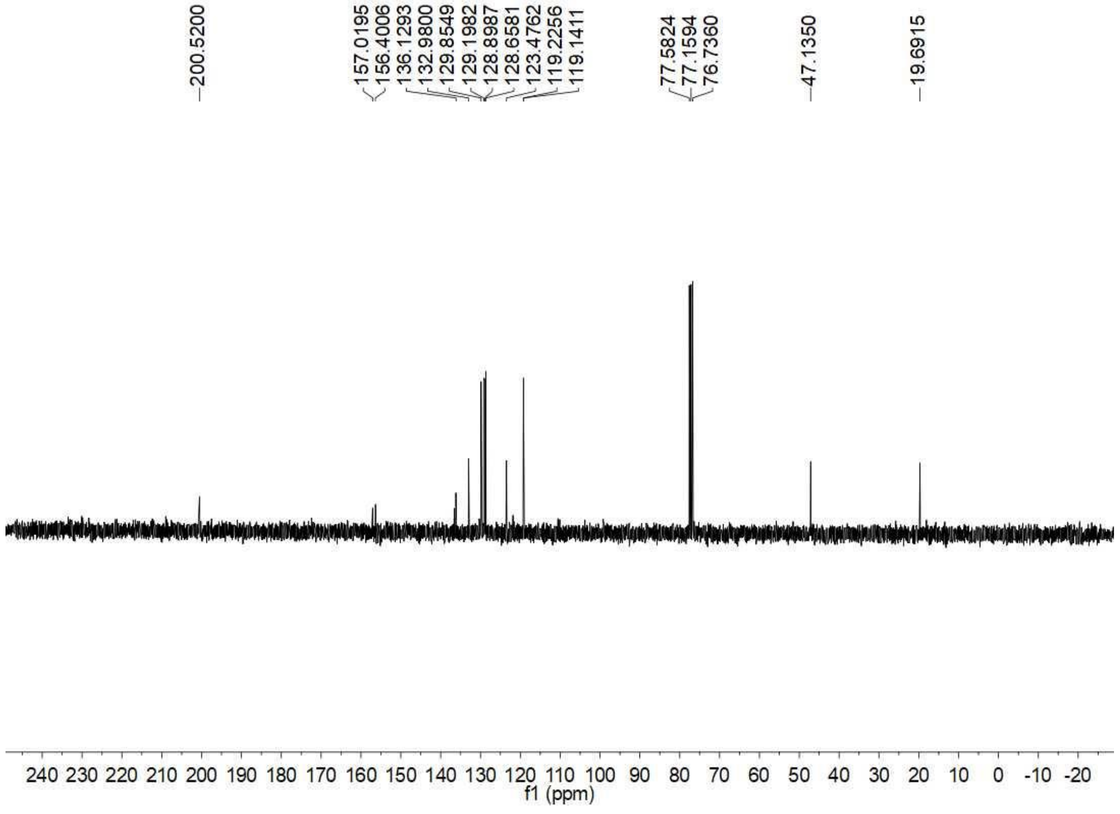

2-(4-Methoxyphenyl)-1-phenylethanone (3aa)

^{1}H NMR (300 MHz, $CDCl_3$, 300 K)

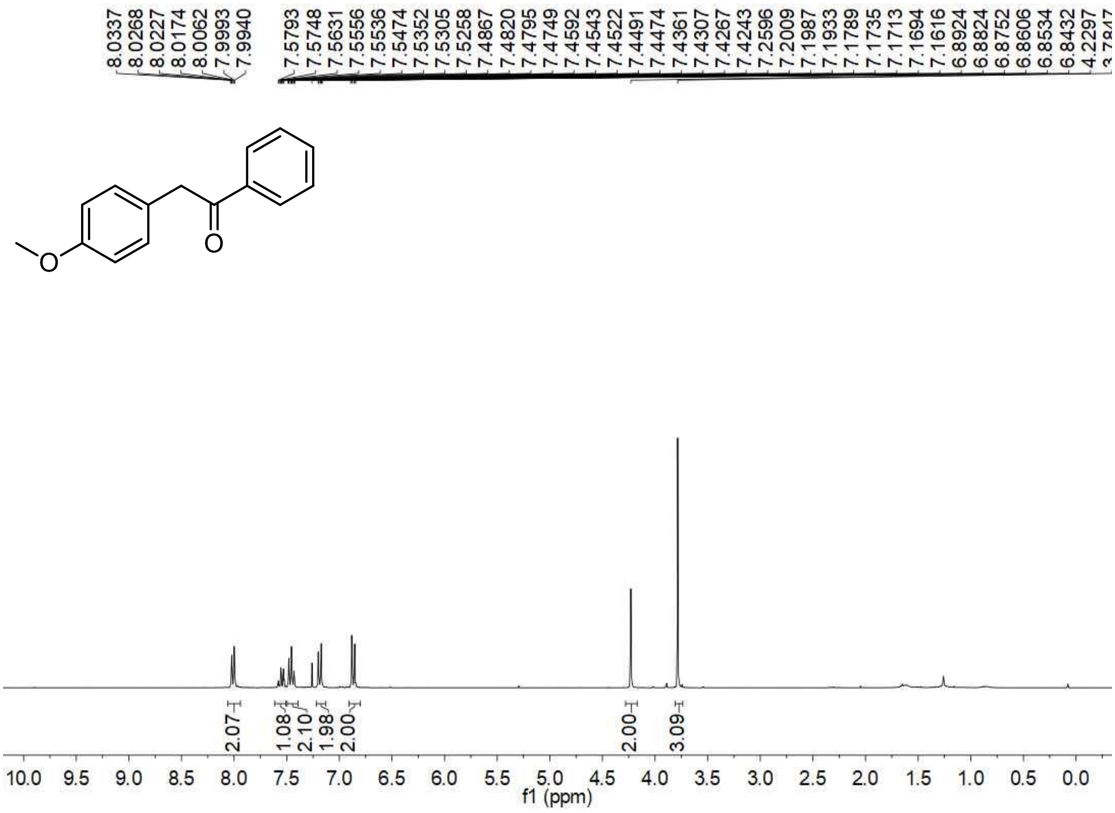

^{13}C NMR (75 MHz, $CDCl_3$, 300 K)

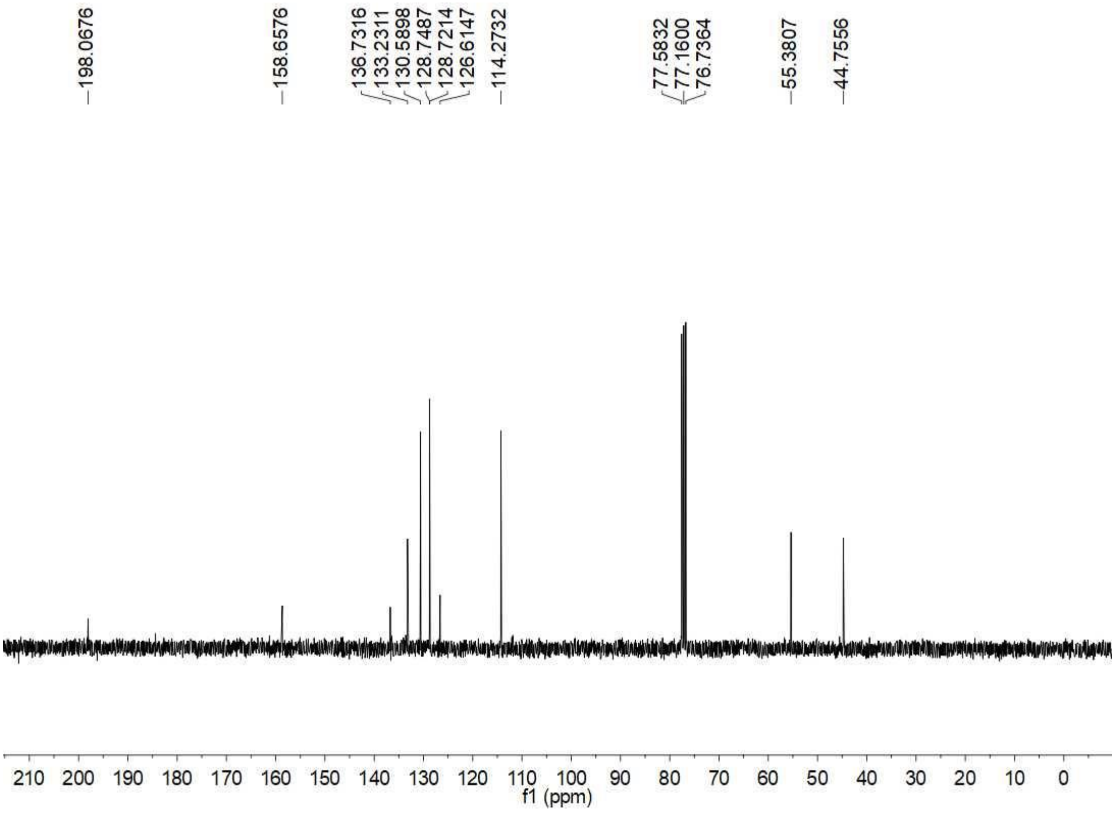

2-(4-Methoxyphenyl)-2-methyl-1-phenylpropan-1-one (3ab)

^{1}H NMR (400 MHz, $CDCl_3$, 300 K)

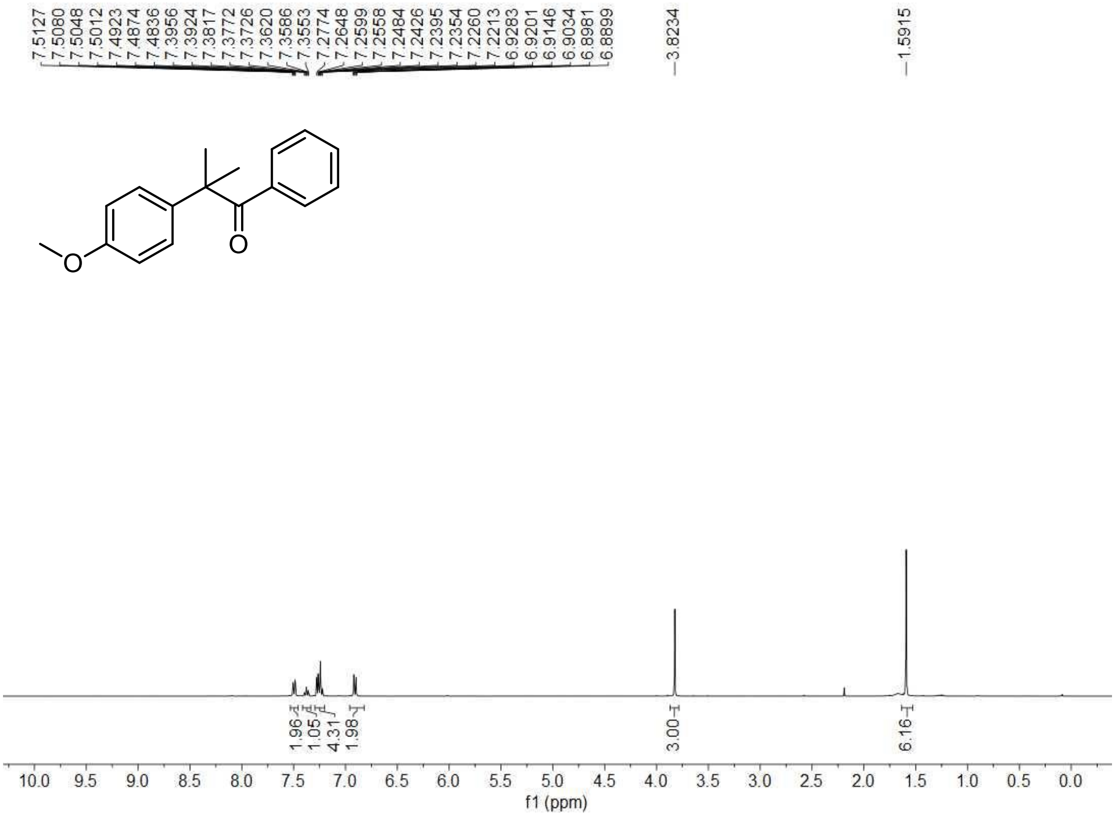

^{13}C NMR (100 MHz, $CDCl_3$, 300 K)

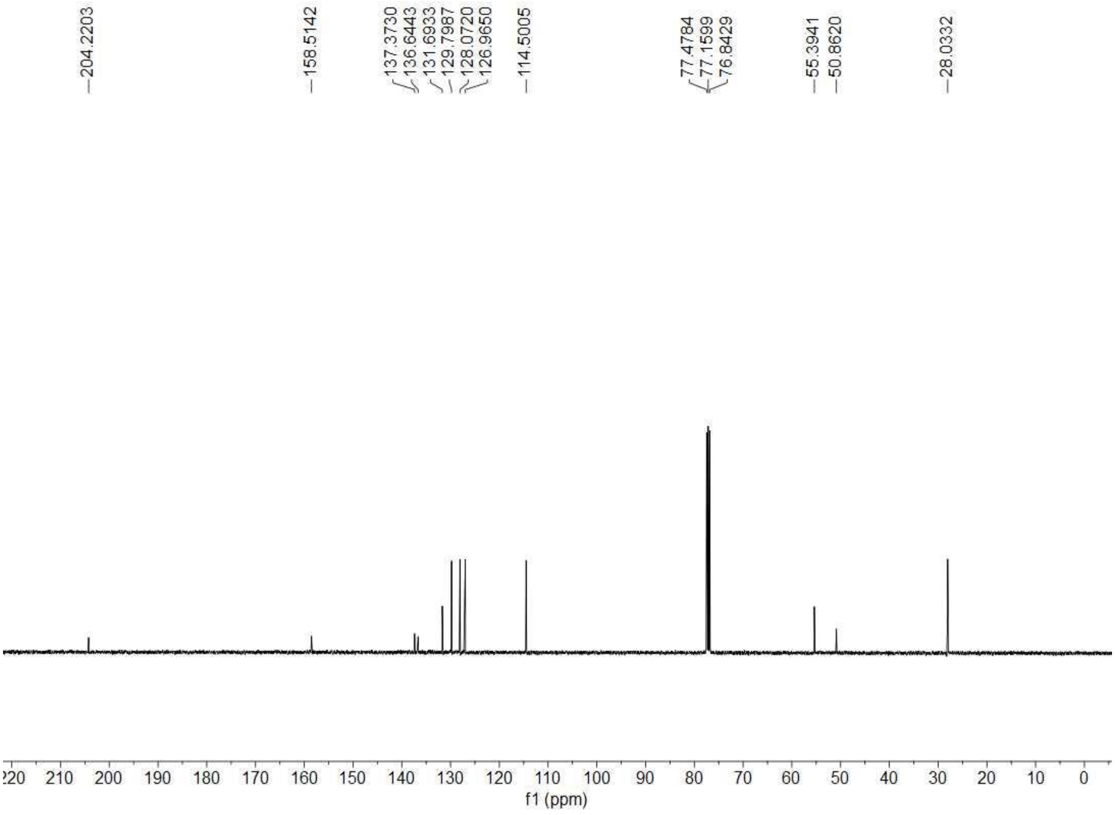

5-Bromo-1,2-diphenylpentan-1-one (3ac)

^{1}H NMR (300 MHz, $CDCl_3$, 300 K)

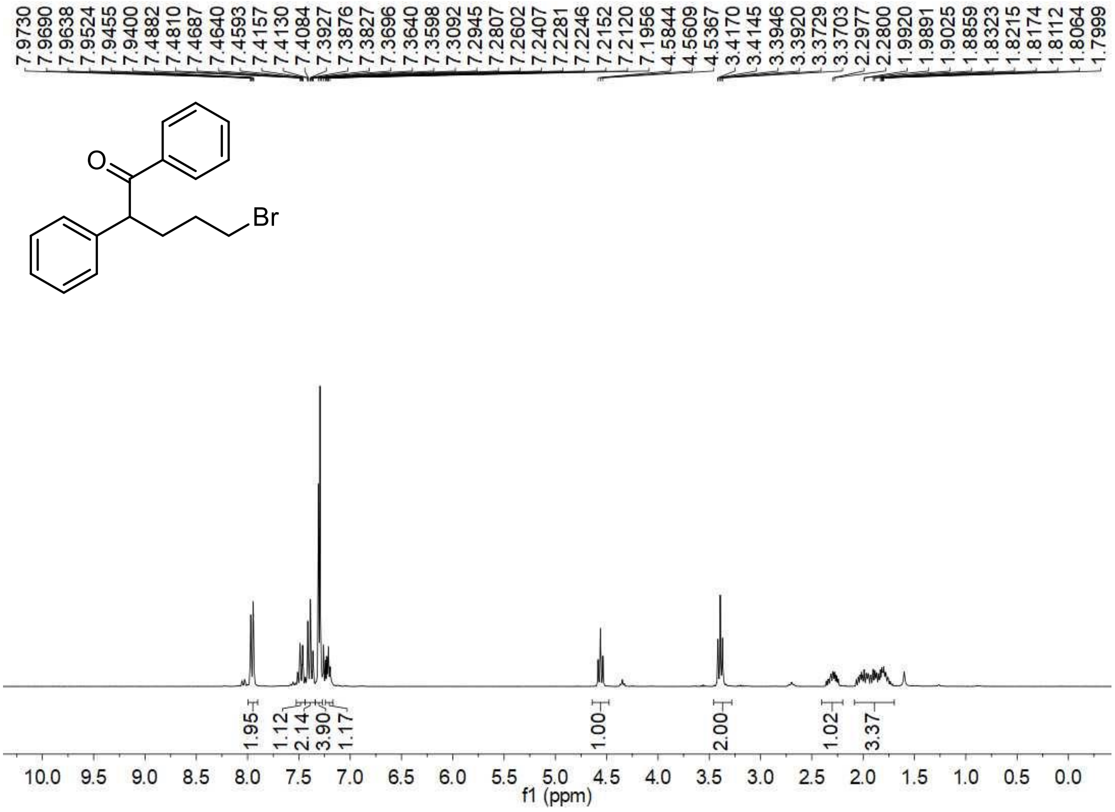

^{13}C NMR (75 MHz, $CDCl_3$, 300 K)

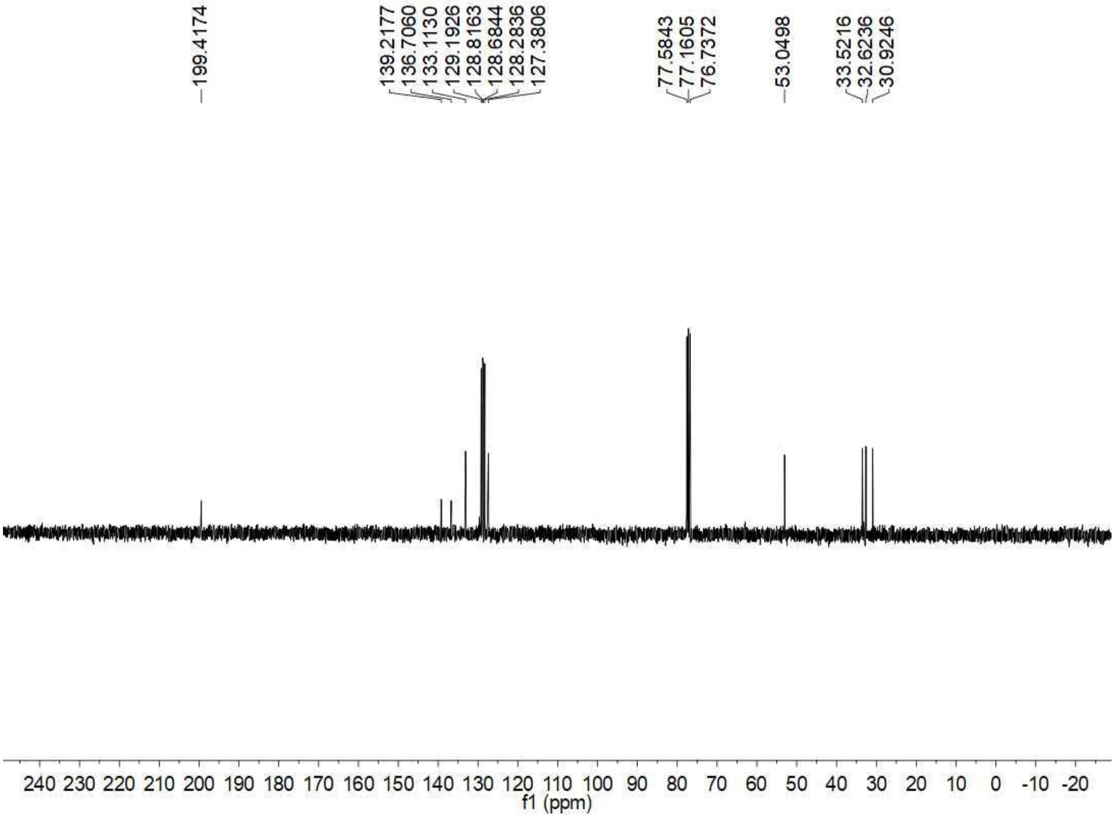

2-(4-(Benzyloxy)phenyl)-1-phenylpropan-1-one (3ad)

^{1}H NMR (300 MHz, $CDCl_3$, 300 K)

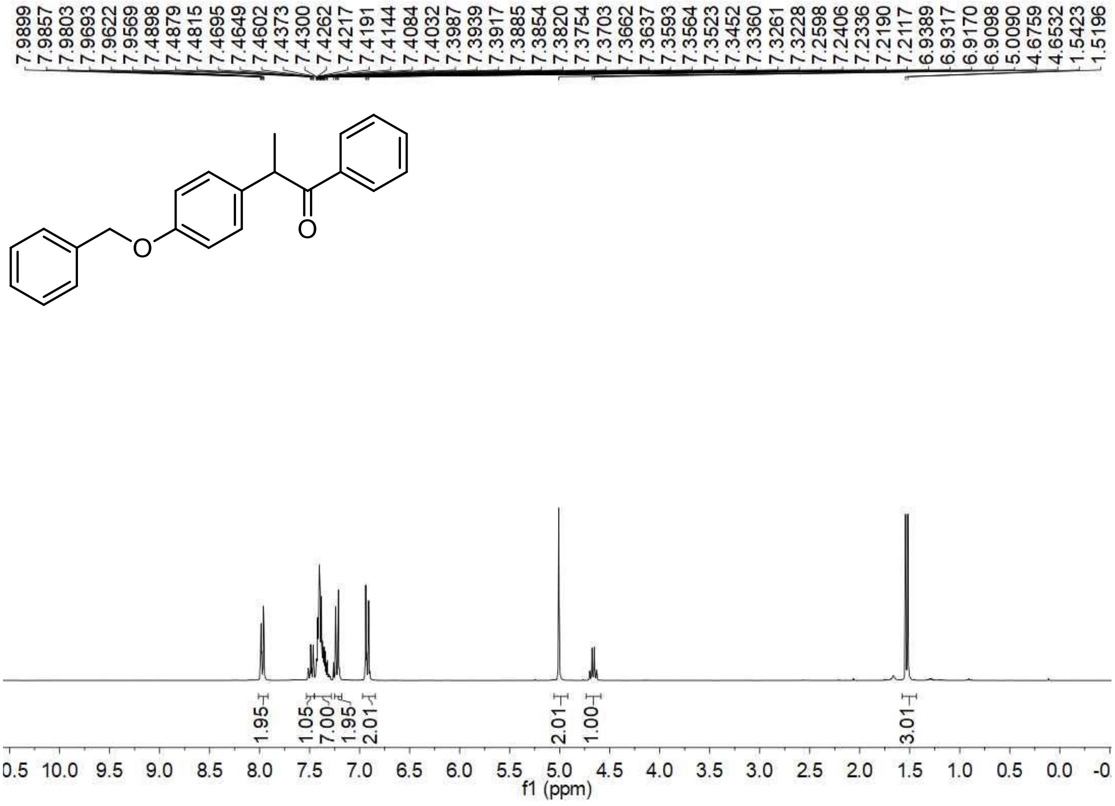

^{13}C NMR (75 MHz, $CDCl_3$, 300 K)

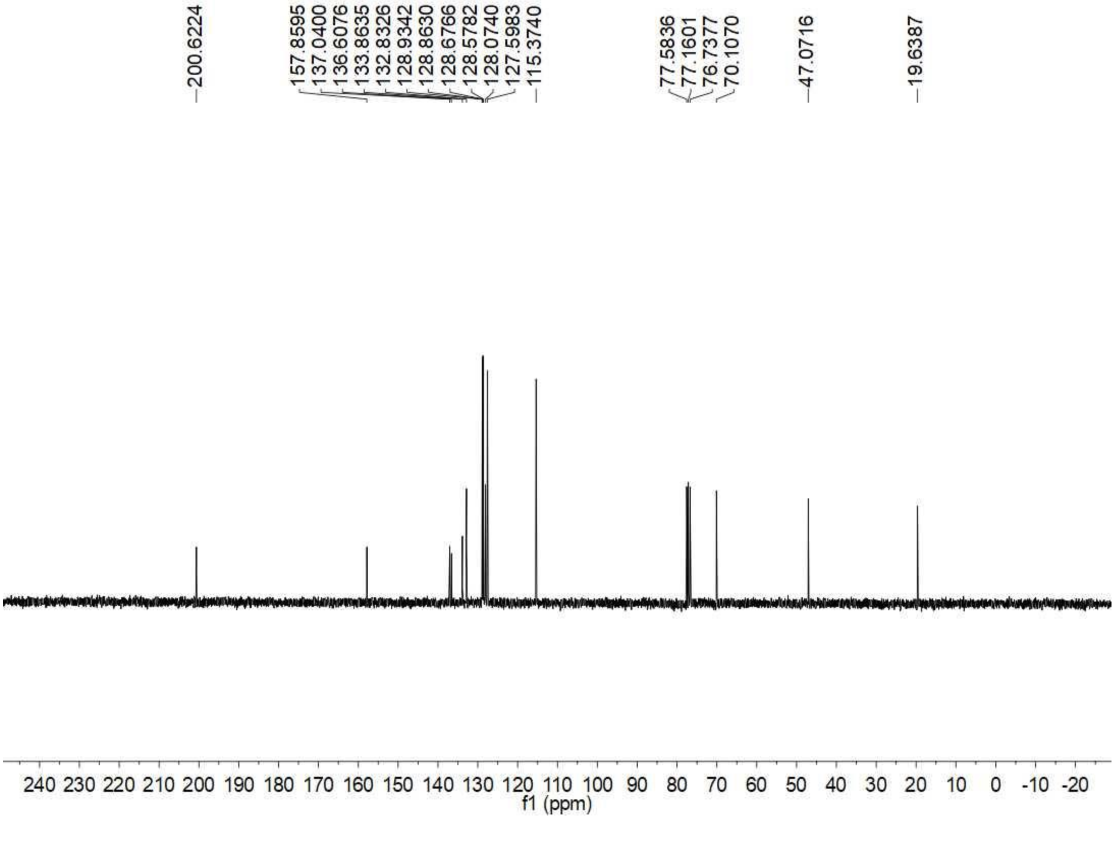

(5-Methoxy-2,3-dihydro-1H-inden-1-yl)(phenyl)methanone (3ae)

^{1}H NMR (300 MHz, $CDCl_3$, 300 K)

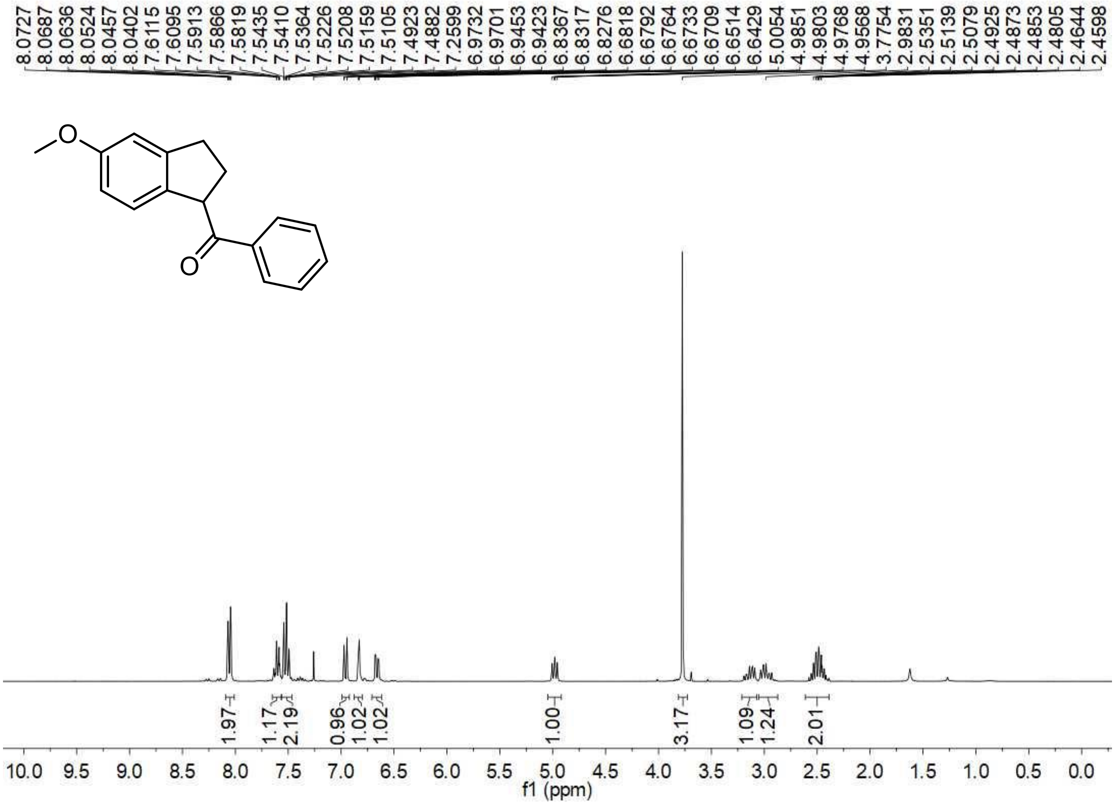

^{13}C NMR (75 MHz, $CDCl_3$, 300 K)

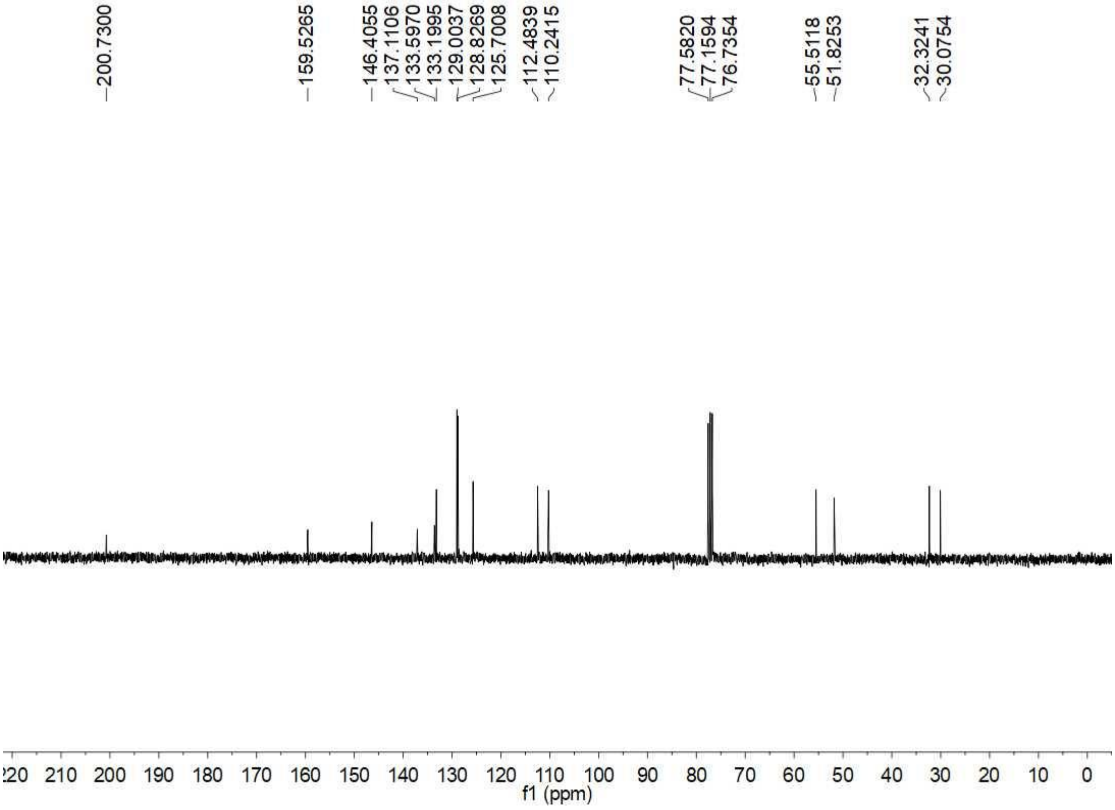

(6-Methoxy-1,2,3,4-tetrahydronaphthalen-1-yl)(phenyl)methanone (3af)

^{1}H NMR (300 MHz, $CDCl_3$, 300 K)

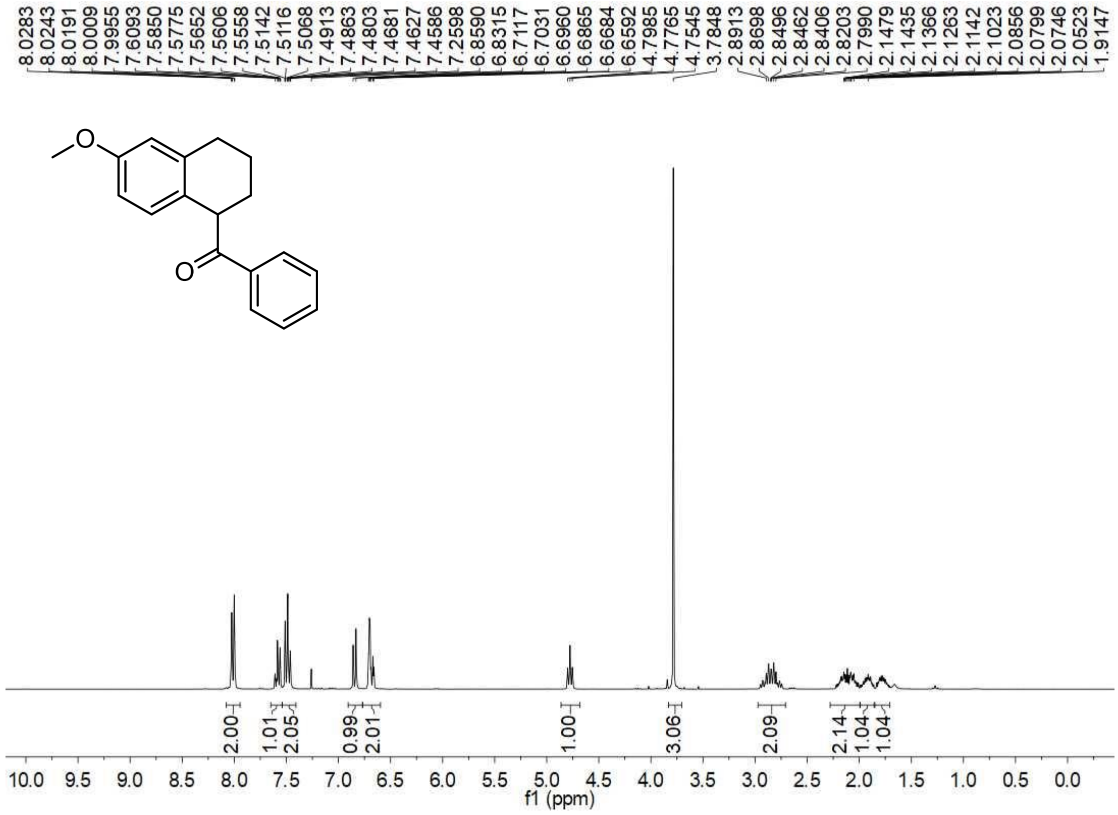

^{13}C NMR (75 MHz, $CDCl_3$, 300 K)

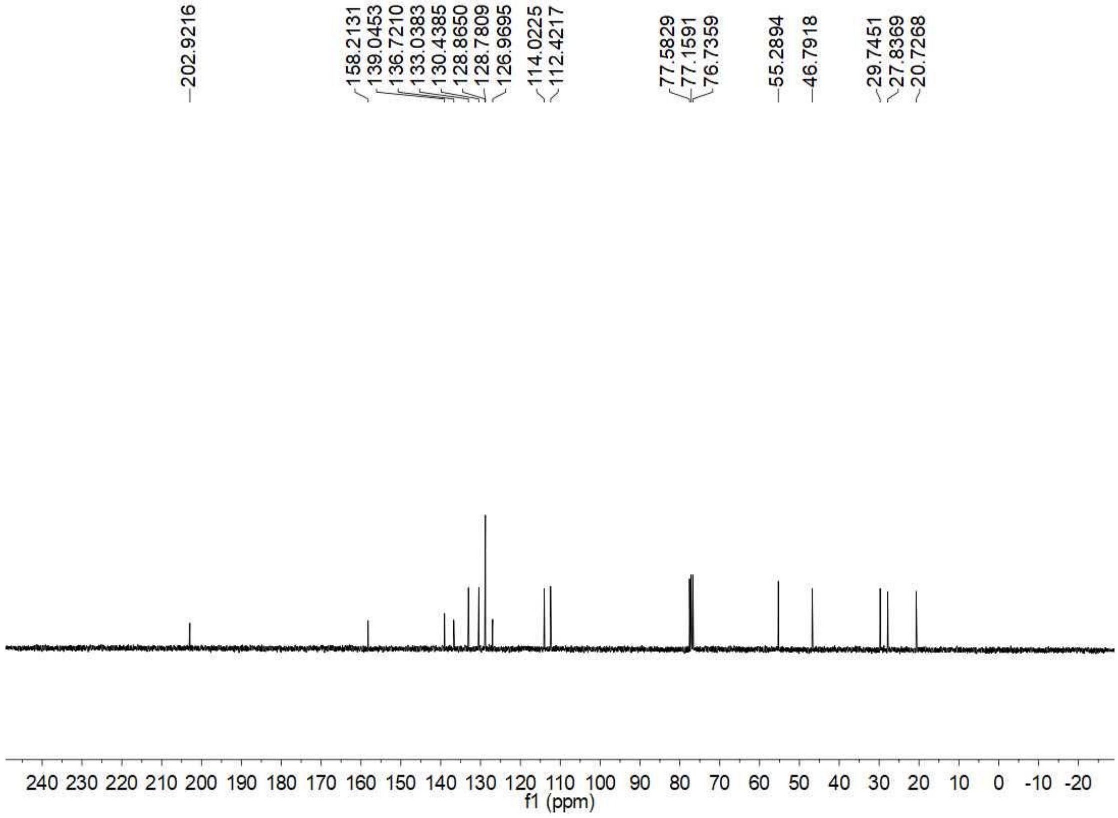

(8-Methoxy-1,2,3,4-tetrahydronaphthalen-1-yl)(phenyl)methanone (3ag)

^{1}H NMR (300 MHz, $CDCl_3$, 300 K)

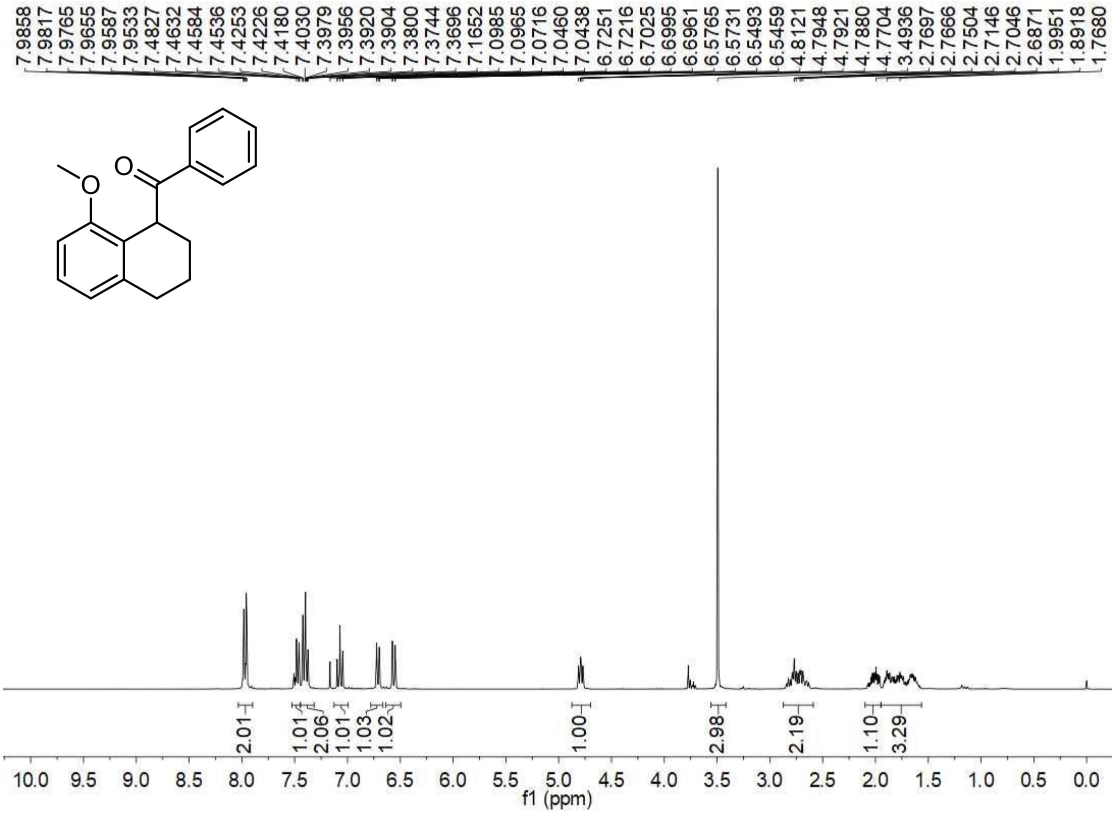

^{13}C NMR (75 MHz, $CDCl_3$, 300 K)

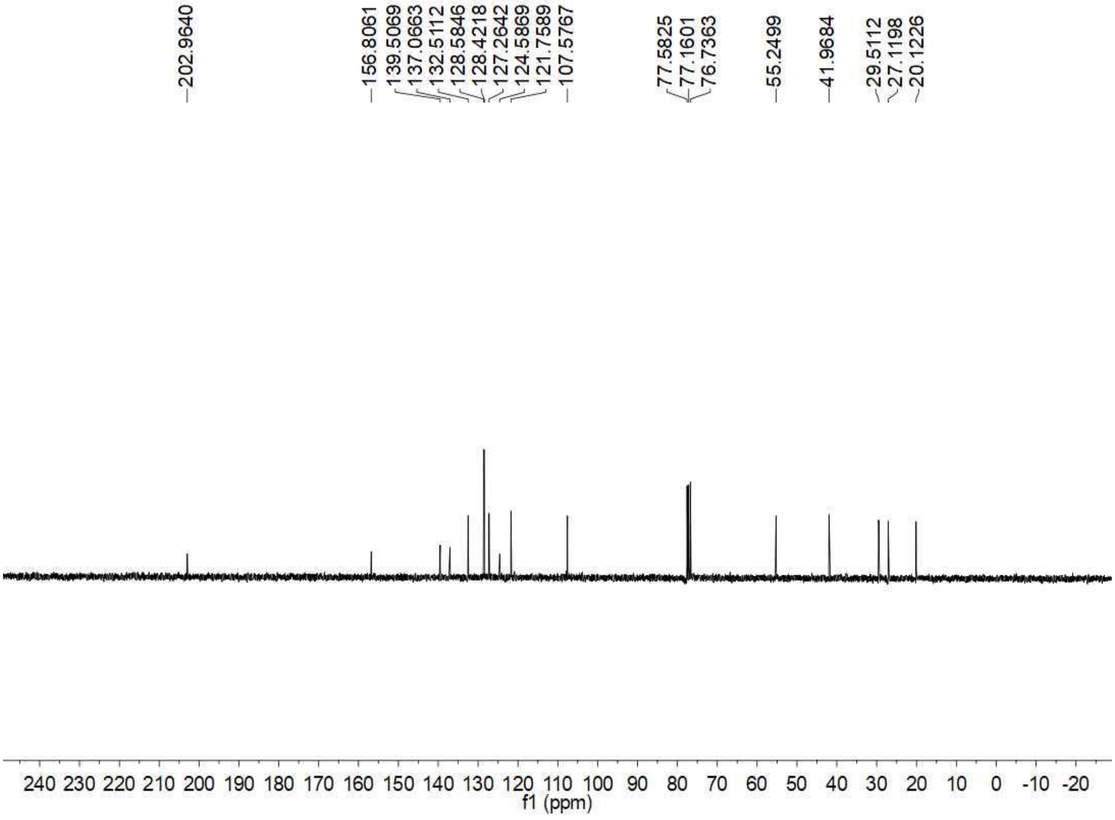

(5,6-Dimethoxy-2,3-dihydro-1H-inden-1-yl)(phenyl)methanone (3ah)

^{1}H NMR (300 MHz, $CDCl_3$, 300 K)

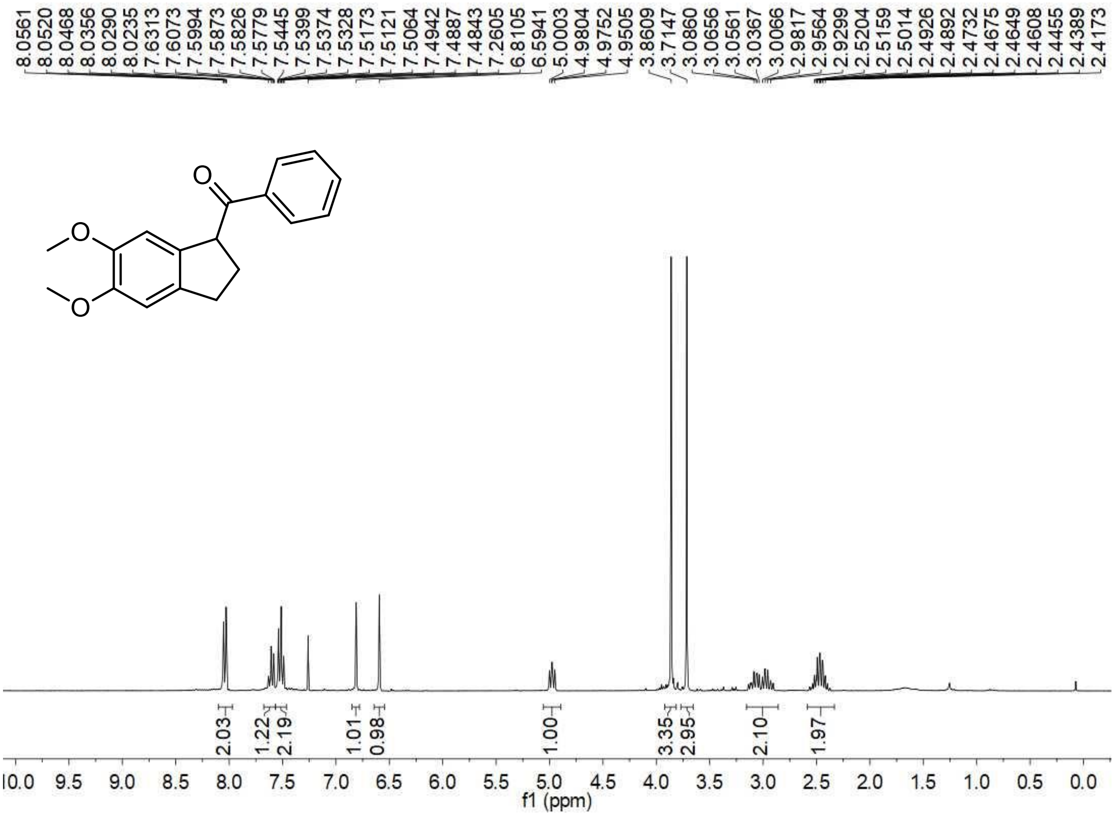

^{13}C NMR (75 MHz, $CDCl_3$, 300 K)

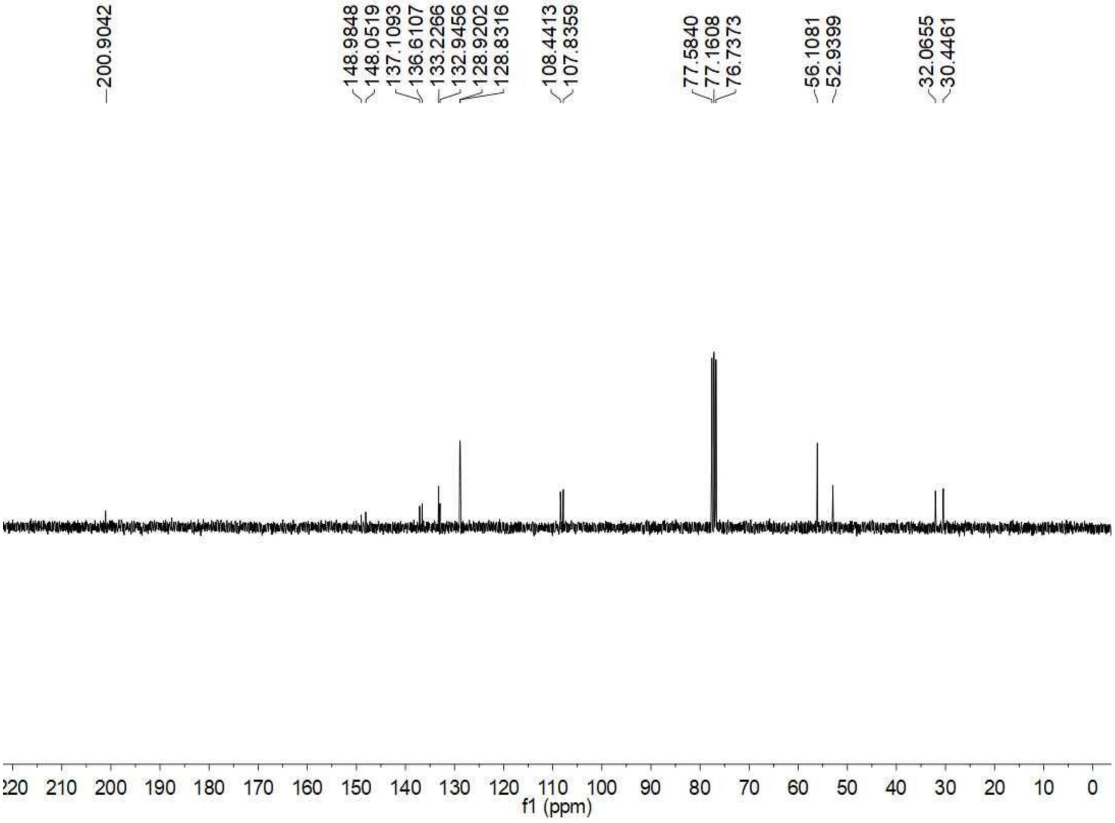

trans-(2-Methyl-2,3-dihydrobenzofuran-3-yl)(phenyl)methanone (3ai)

^{1}H NMR (300 MHz, $CDCl_3$, 300 K)

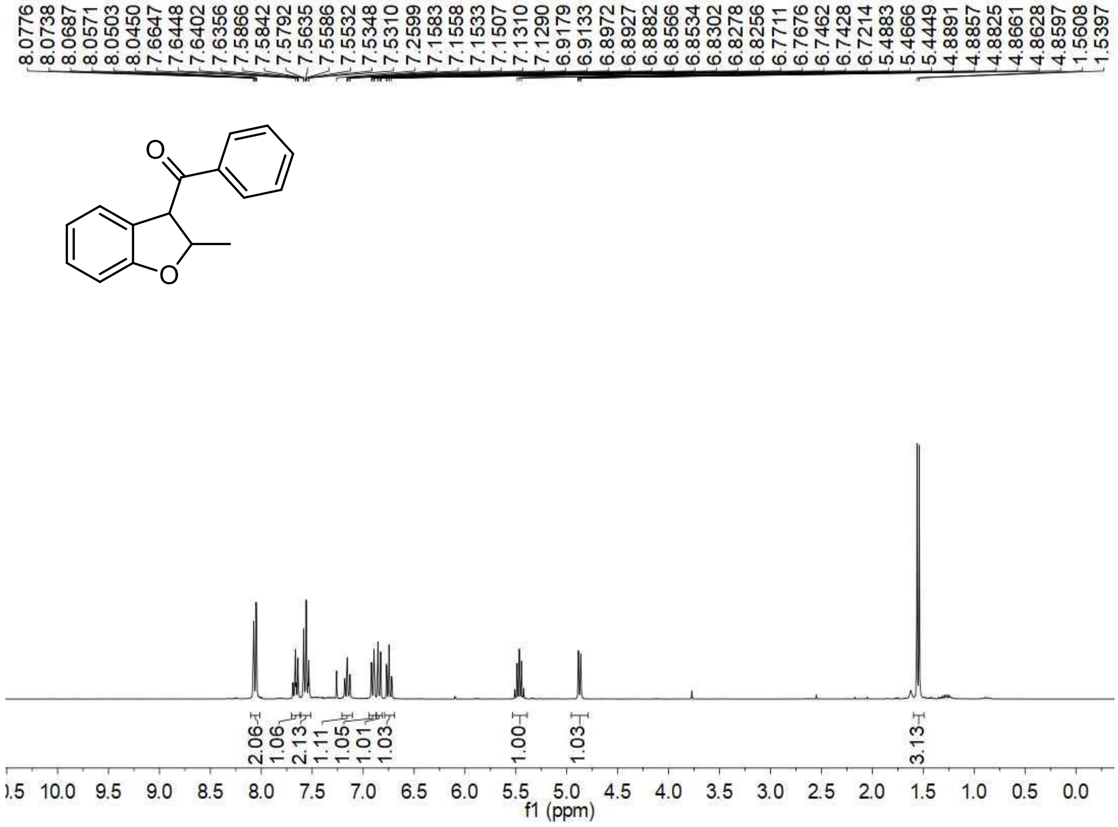

^{13}C NMR (75 MHz, $CDCl_3$, 300 K)

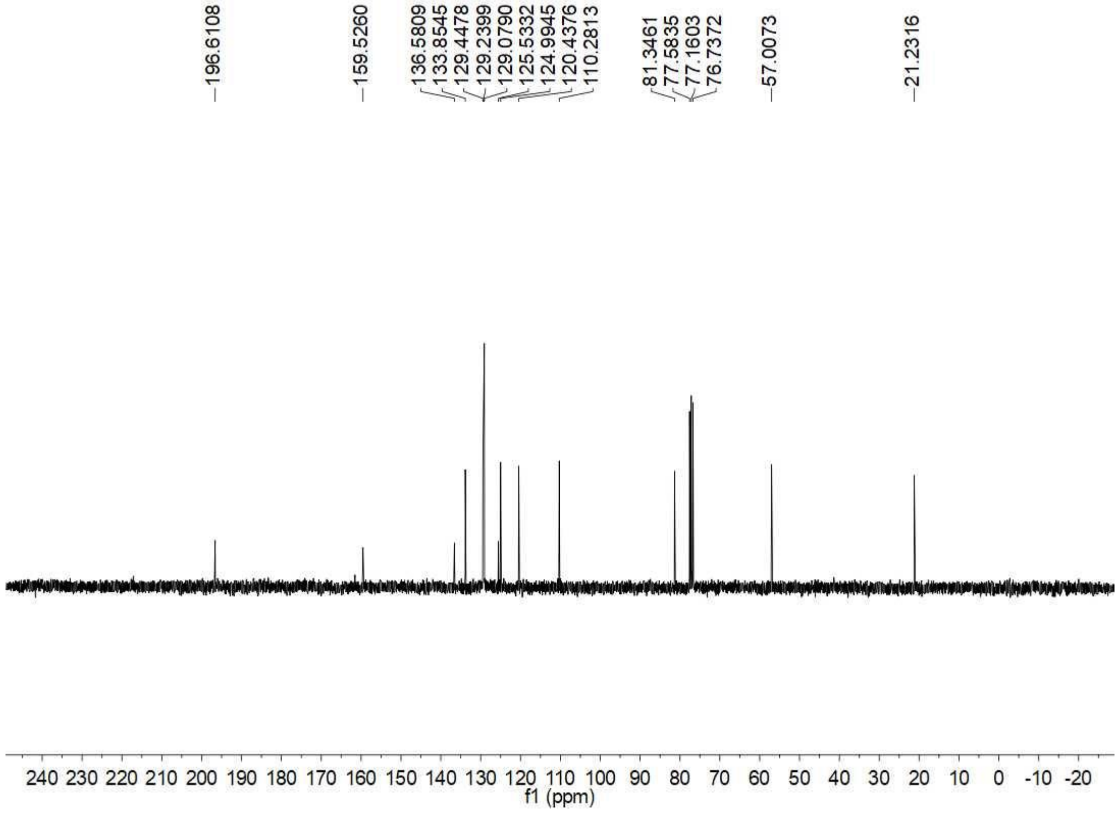

trans-(3-Methylchroman-4-yl)(phenyl)methanone (3aj)

^{1}H NMR (300 MHz, $CDCl_3$, 300 K)

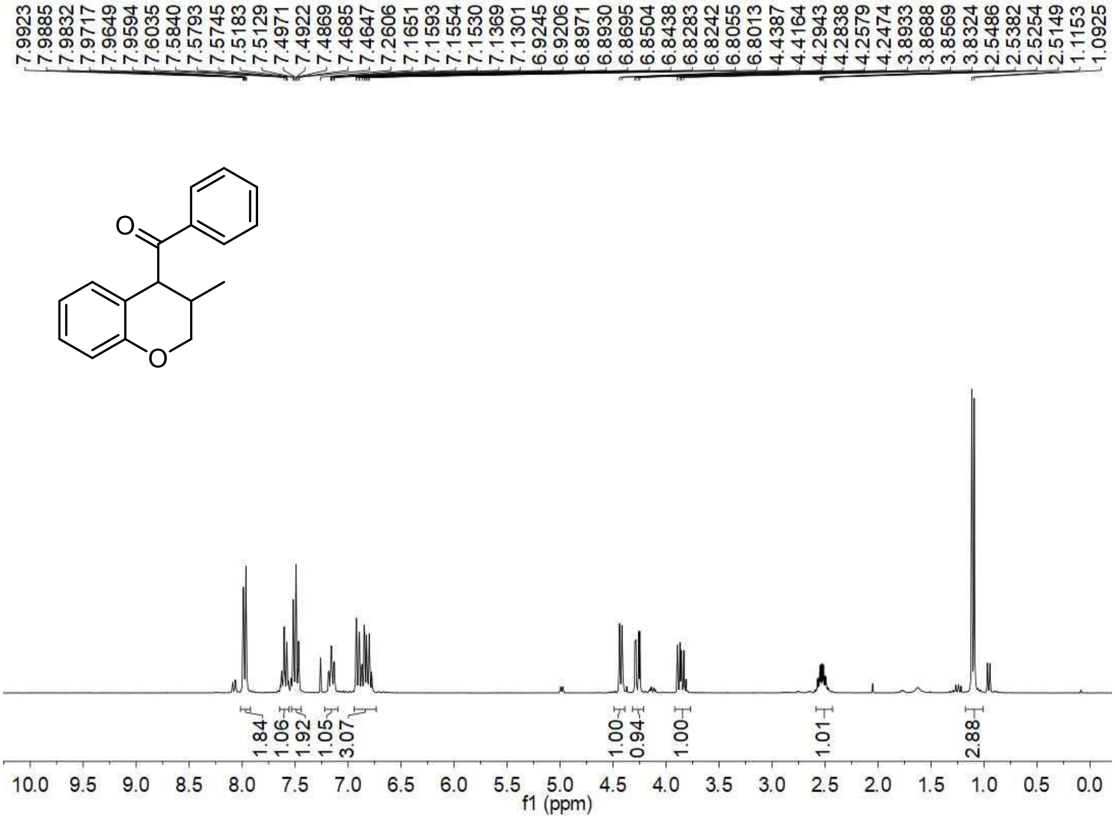

^{13}C NMR (75 MHz, $CDCl_3$, 300 K)

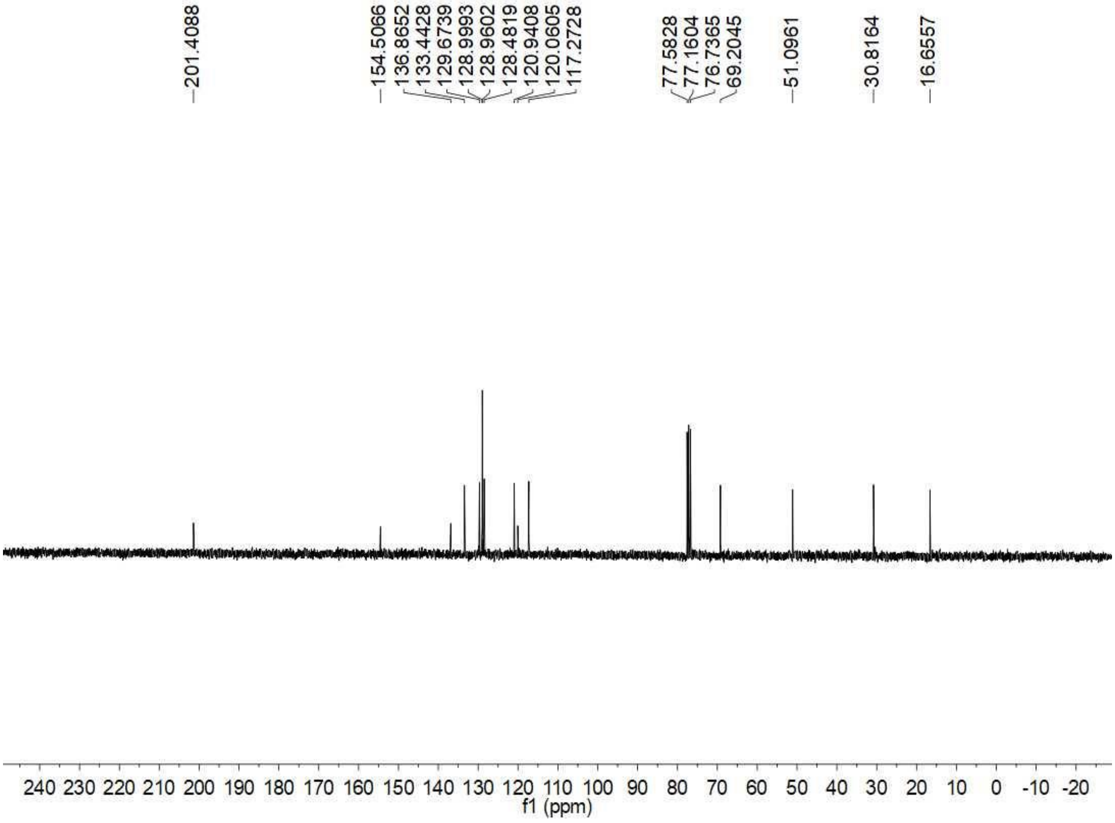

Benzoylation of δ-tocopherol methyl ether (3ak)

^{1}H NMR (400 MHz, $CDCl_3$, 300 K)

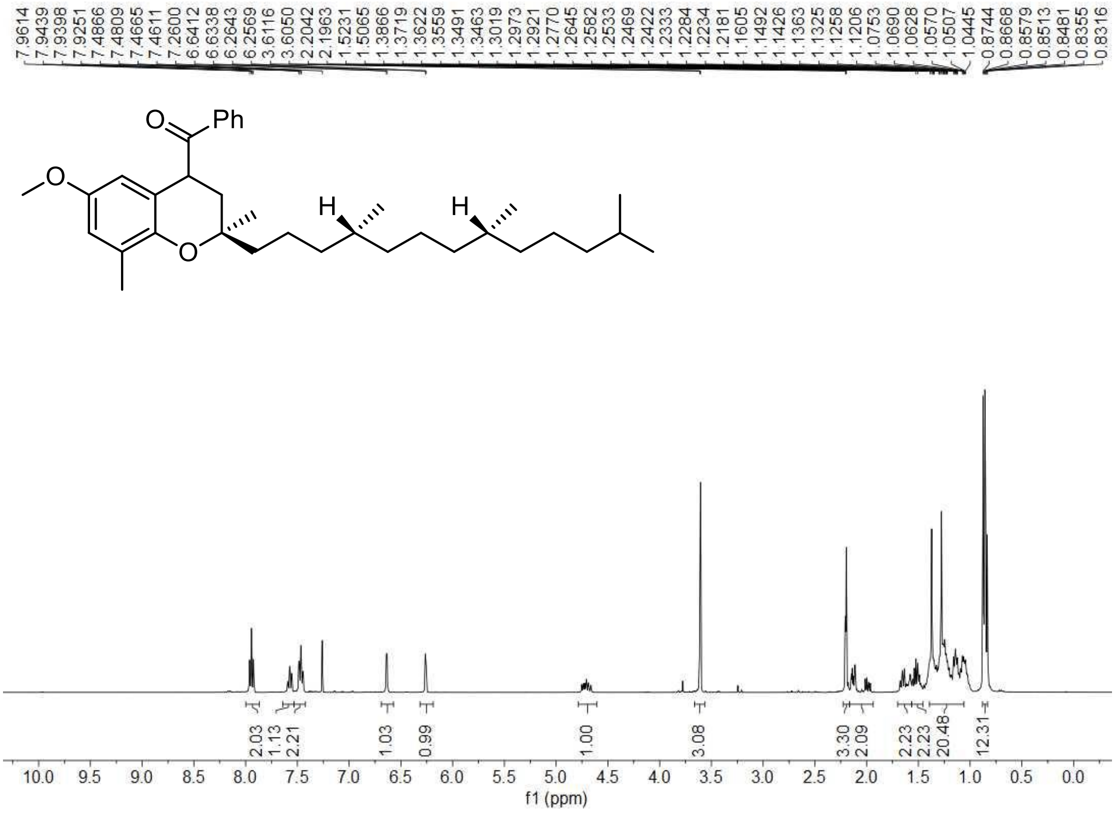

^{13}C NMR (100 MHz, $CDCl_3$, 300 K)

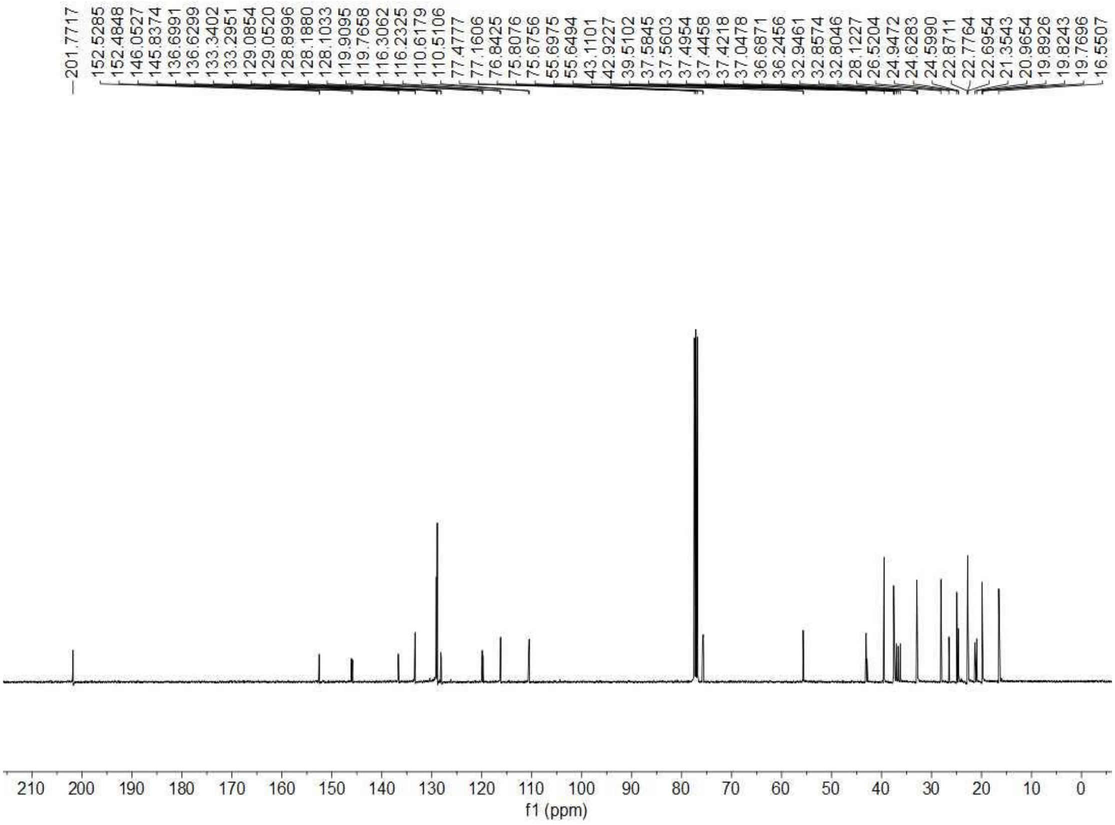

(3R,5S,8R,9S,10S,13S,14S)-10,13-Dimethyl-3-(4-(1-oxo-1-phenylpropan-2-yl)phenoxy)tetradecahydro-1H-cyclopenta[a]phenanthren-17(2H)-one (3al)

^{1}H NMR (300 MHz, $CDCl_3$, 300 K)

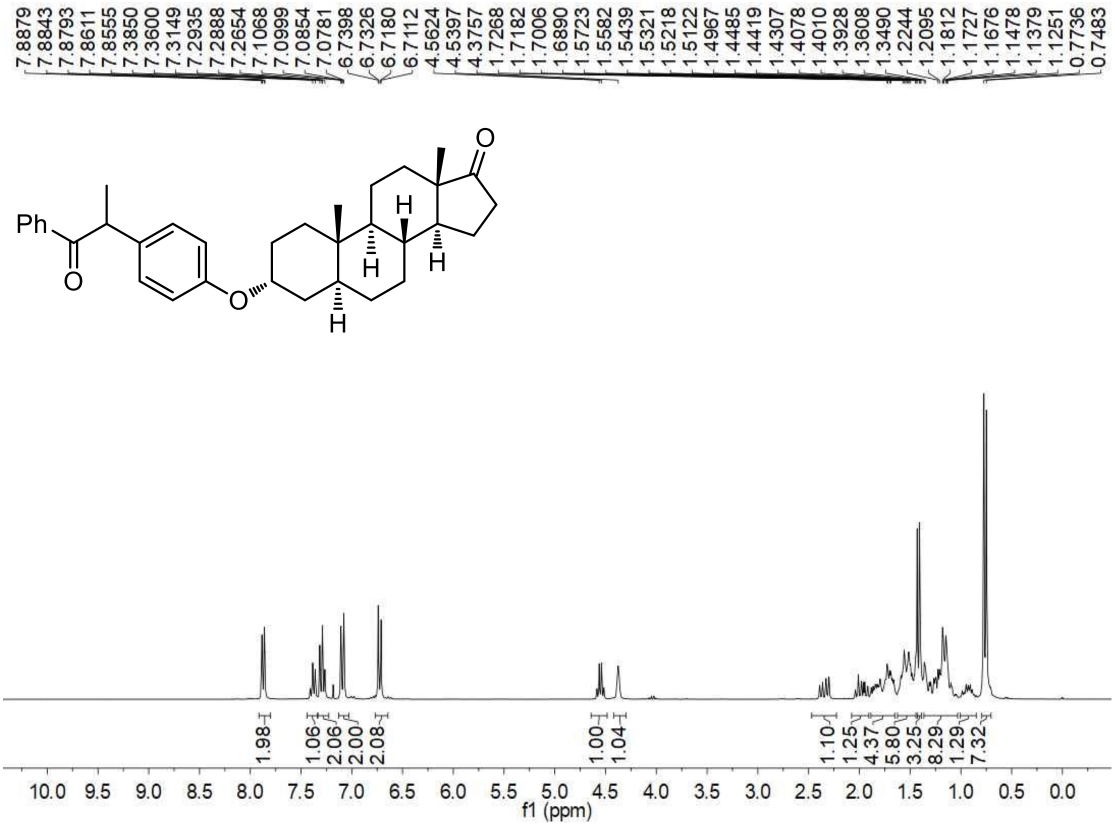

^{13}C NMR (75 MHz, $CDCl_3$, 300 K)

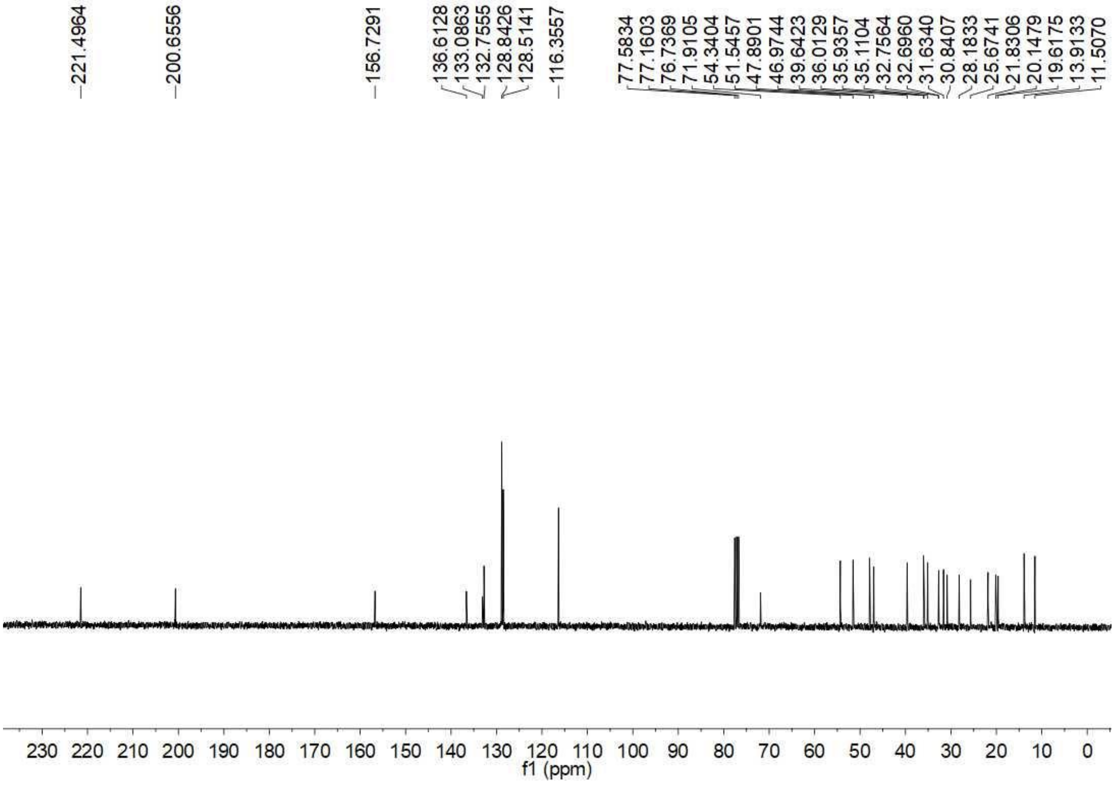

tert-Butyl (2-(3,4-dimethoxyphenyl)-3-oxo-3-phenylpropyl)carbamate (3am)

^{1}H NMR (400 MHz, $CDCl_3$, 300 K)

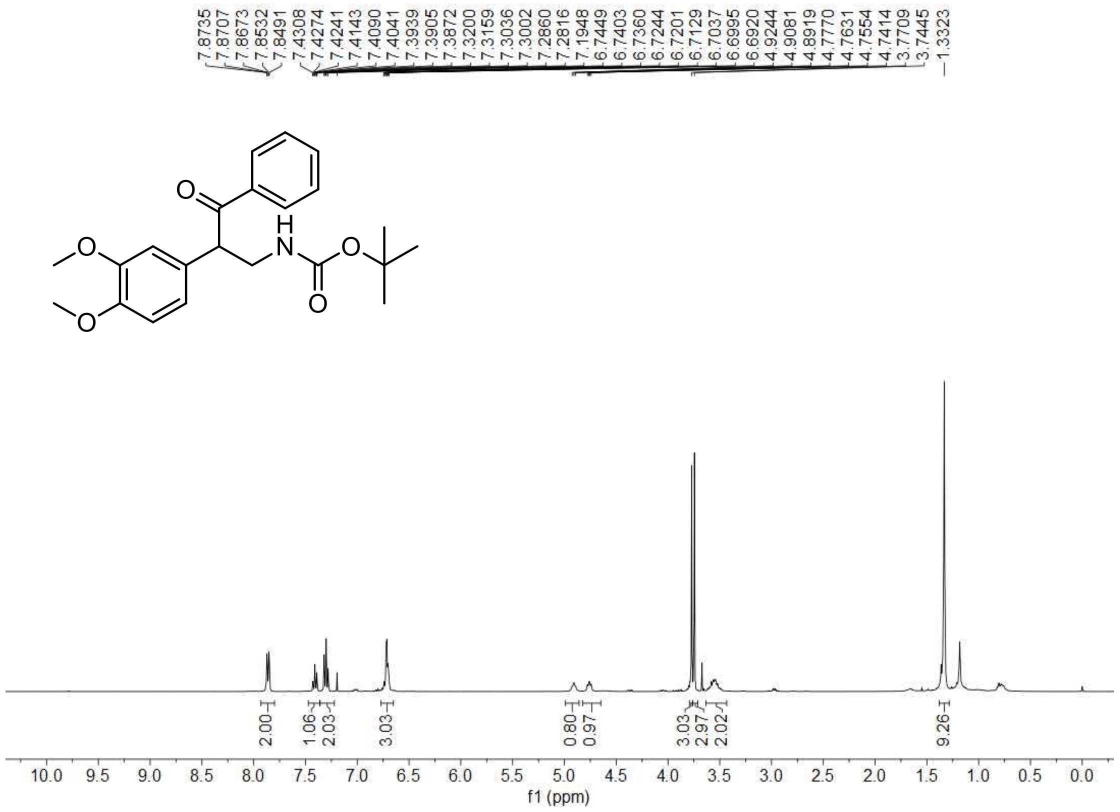

^{13}C NMR (100 MHz, $CDCl_3$, 300 K)

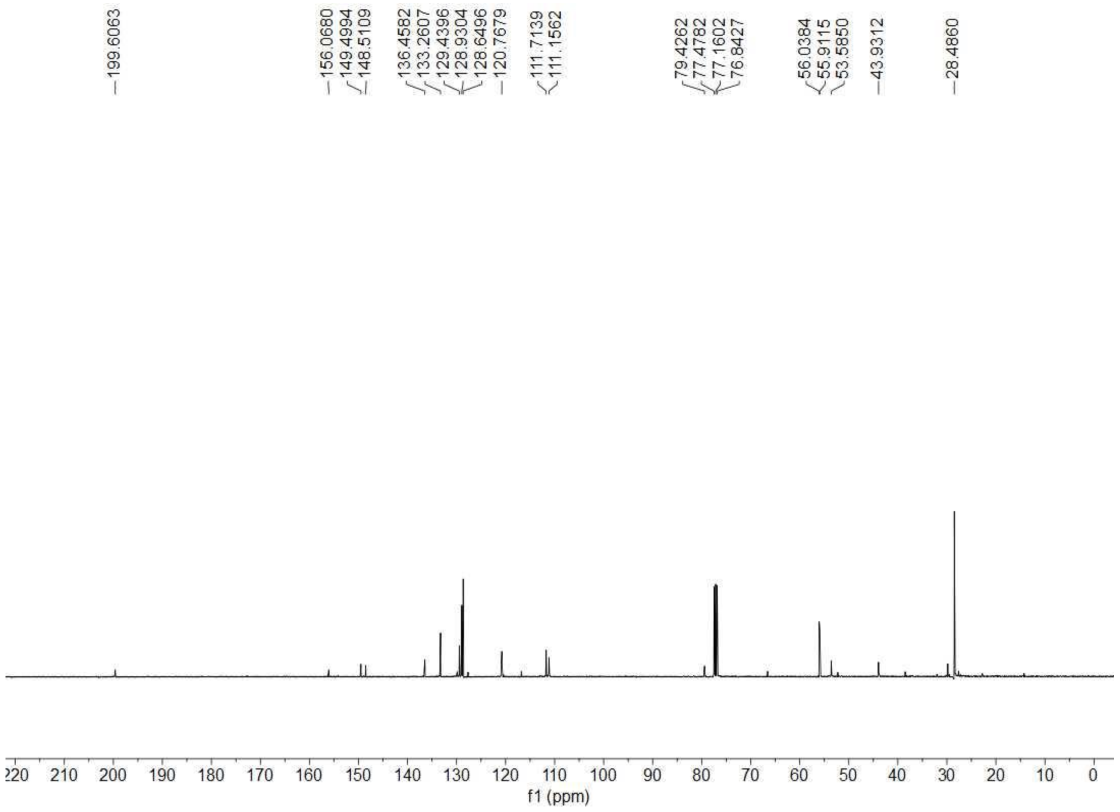

(4-d-Chroman-4-yl)(phenyl)methanone (3y-d_1)

^{1}H NMR (400 MHz, $CDCl_3$, 300 K)

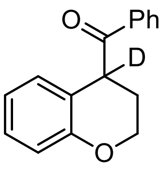

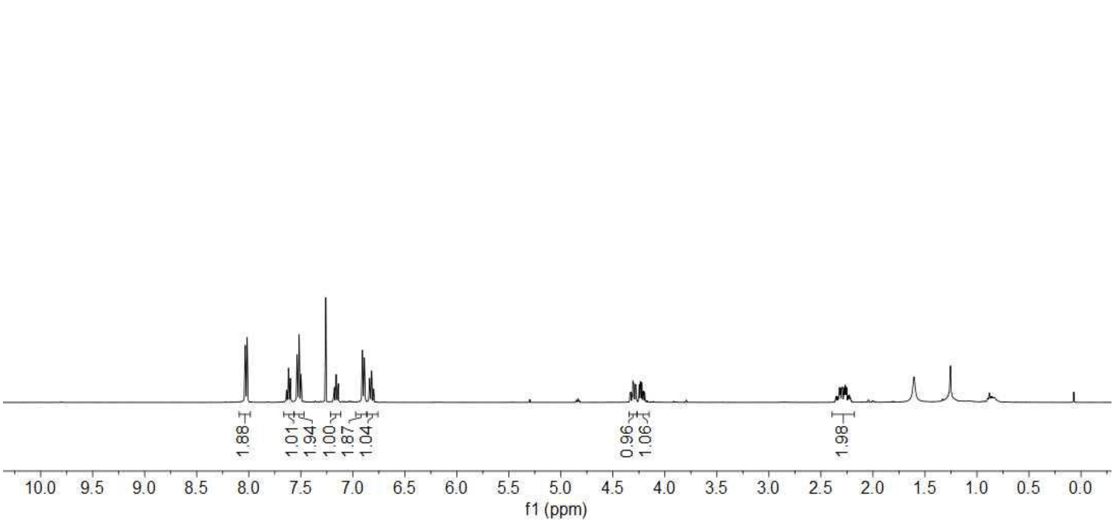

^{13}C NMR (100 MHz, $CDCl_3$, 300 K)

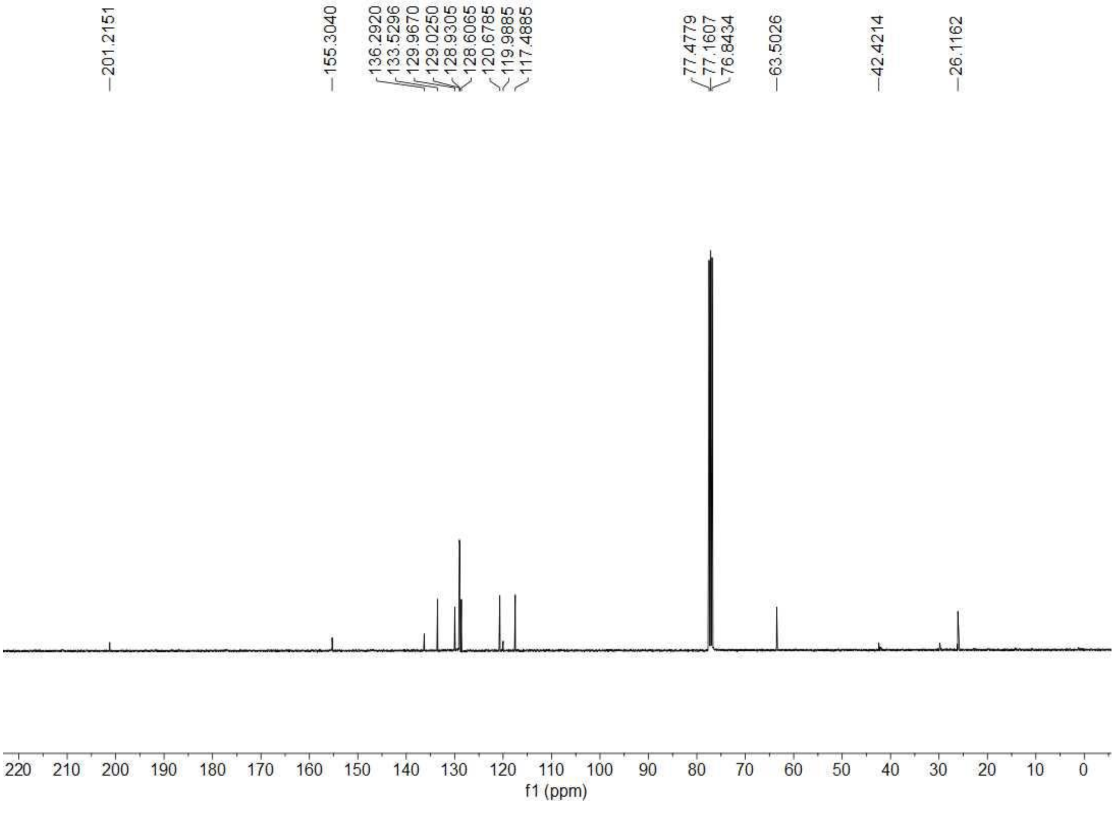

(5-Ethyl-2-methoxyphenyl)(phenyl)methanone

^{1}H NMR (300 MHz, $CDCl_3$, 300 K)

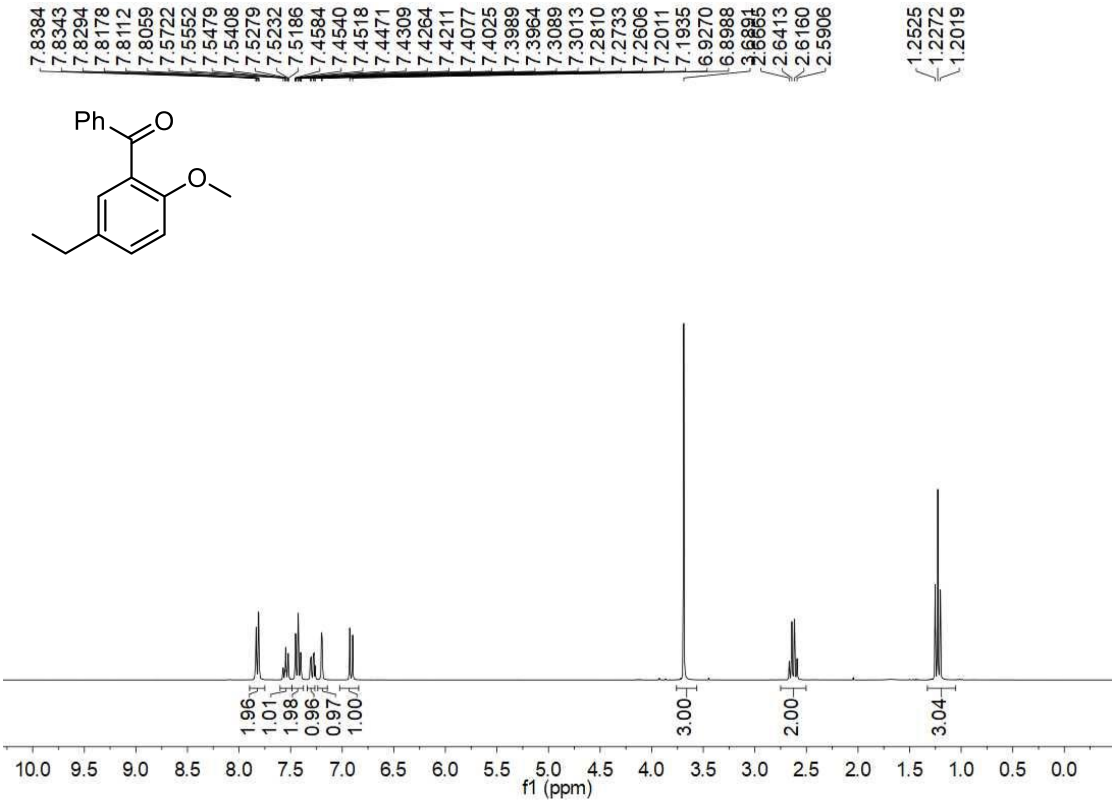

^{13}C NMR (75 MHz, $CDCl_3$, 300 K)

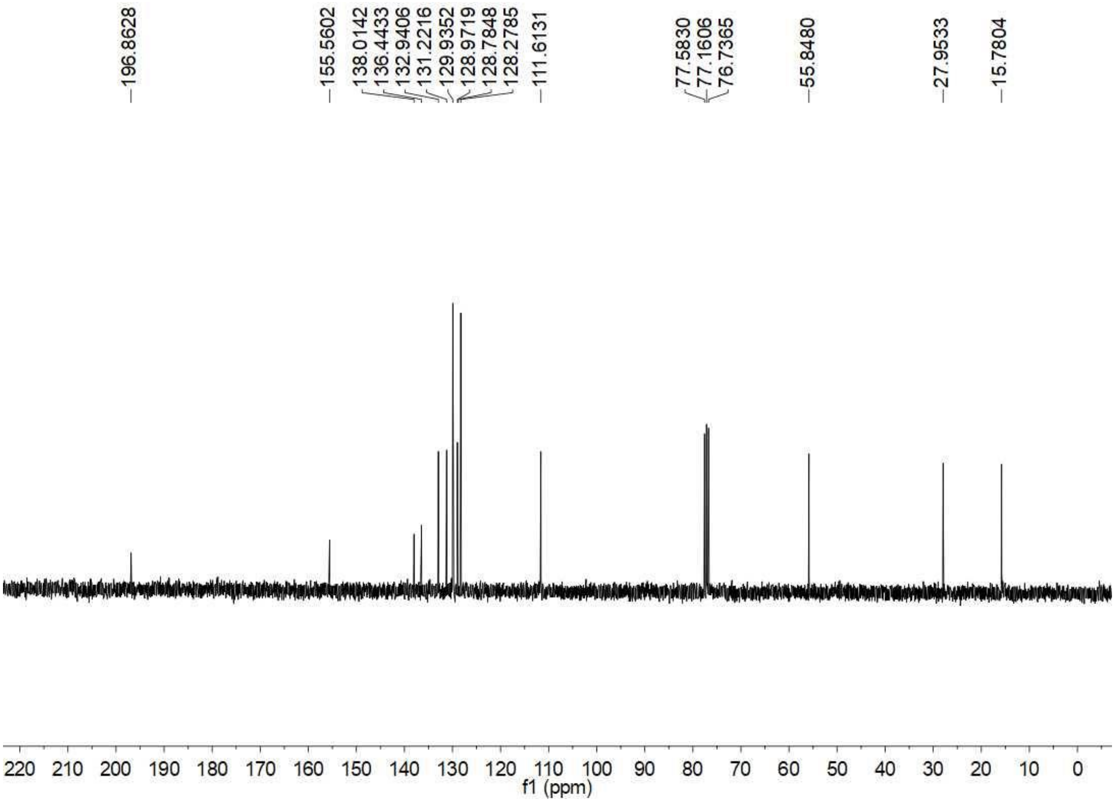

Supplementary references

1. Kerr M. S., Read de Alaniz J. & Rovis T. An efficient synthesis of achiral and chiral 1,2,4-triazolium salts: Bench stable precursors for n-heterocyclic carbenes. *J. Org. Chem.* **70**, 5725-5728 (2005).

2. Ling K. B. & Smith A. D. A-aroyloxyaldehydes: Scope and limitations as alternatives to α-haloaldehydes for NHC-catalysed redox transformations. *Chem. Commun.* **47**, 373-375 (2011).

3. Luo J. & Zhang J. Donor–acceptor fluorophores for visible-light-promoted organic synthesis: Photoredox/Ni dual catalytic C(sp^3)–C(sp^2) cross-coupling. *ACS Catal.* **6**, 873-877 (2016).

4. Lowry M. S., *et al.* Single-layer electroluminescent devices and photoinduced hydrogen production from an ionic iridium(III) complex. *Chem. Mater.* **17**, 5712-5719 (2005).

5. Meng Q.-Y., Döben N. & Studer A. Cooperative NHC and photoredox catalysis for the synthesis of β-trifluoromethylated alkyl aryl ketones. *Angew. Chem. Int. Ed.* **59**, 19956-19960 (2020).

6. Wang Z., Wang X. & Nishihara Y. Nickel-catalysed decarbonylative borylation of aroyl fluorides. *Chem. Commun.* **54**, 13969-13972 (2018).

7. Ueda T., Konishi H. & Manabe K. Palladium-catalyzed fluorocarbonylation using N-formylsaccharin as CO source: General access to carboxylic acid derivatives. *Org. Lett.* **15**, 5370-5373 (2013).

8. Clark J. R., Feng K., Sookezian A. & White M. C. Manganese-catalysed benzylic C(sp^3)–H amination for late-stage functionalization. *Nat. Chem.* **10**, 583-591 (2018).

9. Jouffroy M., Primer D. N. & Molander G. A. Base-free photoredox/nickel dual-catalytic cross-coupling of ammonium alkylsilicates. *J. Am. Chem. Soc.* **138**, 475-478 (2016).

10. Gonçalves C. R., *et al.* Unified approach to the chemoselective α-functionalization of amides with heteroatom nucleophiles. *J. Am. Chem. Soc.* **141**, 18437-18443 (2019).

11. Semmelhack M. F., *et al.* Reaction of aryl and vinyl halides with zerovalent nickel-preparative aspects and the synthesis of alnusone. *J. Am. Chem. Soc.* **103**, 6460-6471 (1981).

12. Hirsh A. J., *et al.* Design, synthesis, and structure-activity relationships of novel 2-substituted pyrazinoylguanidine epithelial sodium channel blockers: Drugs for cystic fibrosis and chronic bronchitis. *J. Med. Chem.* **49**, 4098-4115 (2006).

[13. Chih-Yu Chen A., Laliberte S., Larouche G., Han Y. & McKay D. Spirocyclic piperidinecarboxamide derivatives as renin inhibitors and their preparation and use for the treatment of diseases. Application: WO 2011020193 (2011)

14. Vechorkin O., Proust V. & Hu X. Functional group tolerant Kumada-Corriu-Tamao coupling of nonactivated alkyl halides with aryl and heteroaryl nucleophiles: Catalysis by a nickel pincer complex permits the coupling of functionalized Grignard reagents. *J. Am. Chem. Soc.* **131**, 9756-9766 (2009).

15. Volkov A., Gustafson K. P. J., Tai C.-W., Verho O., Bäckvall J.-E. & Adolfsson H. Mild deoxygenation of aromatic ketones and aldehydes over Pd/C using polymethylhydrosiloxane as the reducing agent. *Angew. Chem. Int. Ed.* **54**, 5122-5126 (2015).

16. Meng Q.-Y., Schirmer T. E., Berger A. L., Donabauer K. & König B. Photocarboxylation of benzylic C–H bonds. *J. Am. Chem. Soc.* **141**, 11393-11397 (2019).

17. Tran B. L., Fulton J. L., Linehan J. C., Lercher J. A. & Bullock R. M. Rh(caac)-catalyzed arene hydrogenation: Evidence for nanocatalysis and sterically controlled site-selective hydrogenation. *ACS Catal.* **8**, 8441-8449 (2018).

18. Lee B. J., DeGlopper K. S. & Yoon T. P. Site-selective alkoxylation of benzylic C−H bonds by photoredox catalysis. *Angew. Chem. Int. Ed.* **59**, 197-202 (2020).

19. Zhang D., Iwai T. & Sawamura M. Iridium-catalyzed alkene-selective transfer hydrogenation with 1,4-dioxane as hydrogen donor. *Org. Lett.* **21**, 5867-5872 (2019).

20. Aujard I., Röme D., Arzel E., Johansson M., de Vos D. & Sterner O. Tridemethylisovelleral, a potent cytotoxic agent. *Bioorgan. Med. Chem.* **13**, 6145-6150 (2005).

21. Zhao G., Yuan L.-Z., Alami M. & Provot O. Chlorotrimethylsilane and sodium iodide: A remarkable metal-free association for the desulfurization of benzylic dithioketals under mild conditions. *Adv. Synth. Catal.* **360**, 2522-2536 (2018).

22. Alesso E. N., Tombari D. G., Moltrasio Iglesias G. Y. & Aguirre J. M. Reactions of some N-acyl-1-alkylamines with polyphosphoric ester ppe: Nuclear magnetic resonance and stereochemistry of reaction products. *Can. J. Chem.* **65**, 2568-2574 (1987).

23. Takeda T. & Terada M. Development of a chiral bis(guanidino)iminophosphorane as an uncharged organosuperbase for the enantioselective amination of ketones. *J. Am. Chem. Soc.* **135**, 15306-15309 (2013).

24. Xia J., Nie Y., Yang G., Liu Y. & Zhang W. Iridium-catalyzed asymmetric hydrogenation of 2H-chromenes: A highly enantioselective approach to isoflavan derivatives. *Org. Lett.* **19**, 4884-4887 (2017).

25. Karimov R. R., Sharma A. & Hartwig J. F. Late stage azidation of complex molecules. *ACS Cent. Sci.* **2**, 715-724 (2016).

26. He Z., Song F., Sun H. & Huang Y. Transition-metal-free Suzuki-type cross-coupling reaction of benzyl halides and boronic acids via 1,2-metalate shift. *J. Am. Chem. Soc.* **140**, 2693-2699 (2018).

27. Shaikh R. S., Ghosh I. & König B. Direct C−H phosphonylation of electron-rich arenes and heteroarenes by visible-light photoredox catalysis. *Chem. Eur. J.* **23**, 12120-12124 (2017).

28. Zhang W., *et al.* Enantioselective cyanation of benzylic C–H bonds via copper-catalyzed radical relay. *Science* **353**, 1014-1018 (2016).

29. Marelli E., Corpet M., Davies S. R. & Nolan S. P. Palladium-catalyzed α-arylation of arylketones at low catalyst loadings. *Chem. Eur. J.* **20**, 17272-17276 (2014).

30. Liu F., Hu Y.-Y., Li D., Zhou Q. & Lu J.-M. N-heterocyclic carbene-palladacyclic complexes: Synthesis, characterization and their applications in the C–N coupling and α-arylation of ketones using aryl chlorides. *Tetrahedron* **74**, 5683-5690 (2018).

31. Wolters A. T., Hornillos V., Heijnen D., Giannerini M. & Feringa B. L. One-pot, modular approach to functionalized ketones via nucleophilic addition of alkyllithium reagents to benzamides and Pd-catalyzed α-arylation. *ACS Catal.* **6**, 2622-2625 (2016).

32. Blay G., Fernández I., Monje B., Muñoz M. C., Pedro J. R. & Vila C. Enantioselective synthesis of 2-substituted-1,4-diketones from (s)-mandelic acid enolate and α,β-enones. *Tetrahedron* **62**, 9174-9182 (2006).

33. Zhang Z., Liu Y., Gong M., Zhao X., Zhang Y. & Wang J. Palladium-catalyzed carbonylation/acyl migratory insertion sequence. *Angew. Chem. Int. Ed.* **49**, 1139-1142 (2010).

34. Li J. & Wang Z.-X. Nickel-catalyzed transformation of aryl 2-pyridyl ethers via cleavage of the carbon–oxygen bond: Synthesis of mono-α-arylated ketones. *Synthesis* **50**, 3217-3223 (2018).

35. Chen X., Chen Z. & So C. M. Exploration of aryl phosphates in palladium-catalyzed mono-α-arylation of aryl and heteroaryl ketones. *J. Org. Chem.* **84**, 6337-6346 (2019).

36. Fox J. M., Huang X., Chieffi A. & Buchwald S. L. Highly active and selective catalysts for the formation of α-aryl ketones. *J. Am. Chem. Soc.* **122**, 1360-1370 (2000).

37. Ashikari Y., Nokami T. & Yoshida J.-i. Integrated electrochemical–chemical oxidation mediated by alkoxysulfonium ions. *J. Am. Chem. Soc.* **133**, 11840-11843 (2011).
